# Identification of 24-*O*-β-d-Glycosides and 7-Deoxy-Analogues of Okadaic Acid and Dinophysistoxin-1 and -2 in Extracts from *Dinophysis* Blooms, *Dinophysis* and *Prorocentrum* Cultures, and Shellfish in Europe, North America and Australasia

**DOI:** 10.3390/toxins13080510

**Published:** 2021-07-21

**Authors:** Alistair L. Wilkins, Thomas Rundberget, Morten Sandvik, Frode Rise, Brent K. Knudsen, Jane Kilcoyne, Beatriz Reguera, Pilar Rial, Elliott J. Wright, Sabrina D. Giddings, Michael J. Boundy, Cheryl Rafuse, Christopher O. Miles

**Affiliations:** 1 Norwegian Veterinary Institute, P.O. Box 64, NO-1431 Ås, Norway; wilkinsalw@hotmail.com (A.L.W.); thomas.rundberget@niva.no (T.R.); morten.sandvik@vetinst.no (M.S.); 2 School of Science and Engineering, University of Waikato, Private Bag 3105, Hamilton 3240, New Zealand; brent.knudsen@ixom.com; 3 Norwegian Institute for Water Research, Gaustadalléen 21, NO-0349 Oslo, Norway; 4 Department of Chemistry, University of Oslo, P.O. Box 1033, Blindern, NO-0315 Oslo, Norway; frode.rise@kjemi.uio.no; 5 Marine Institute, Rinville, Oranmore, County Galway H91 R673, Ireland; jane.kilcoyne@marine.ie; 6 Centro Oceanográfico de Vigo (IEO, CSIC), Subida a Radio Faro 50, 36390 Vigo, Spain; beatriz.reguera@ieo.es (B.R.); pilar.rial@ieo.es (P.R.); 7 Biotoxin Metrology, National Research Council, 1411 Oxford St., Halifax, NS B3H 3Z1, Canada; elliott.wright@nrc.ca (E.J.W.); sabrina.giddings@nrc.ca (S.D.G.); cheryl.rafuse@nrc.ca (C.R.); 8 Cawthron Institute, 98 Halifax Street East, Nelson 7010, New Zealand; michael.boundy@cawthron.org.nz

**Keywords:** *Dinophysis*, *Prorocentrum*, okadaic acid, dinophysistoxin, glucoside, 7-deoxyokadaic acid, 7-deoxydinophysistoxin, LC–HRMS, NMR

## Abstract

Two high-mass polar compounds were observed in aqueous side-fractions from the purification of okadaic acid (**1**) and dinophysistoxin-2 (**2**) from *Dinophysis* blooms in Spain and Norway. These were isolated and shown to be 24-*O*-β-d-glucosides of **1** and **2** (**4** and **5**, respectively) by nuclear magnetic resonance (NMR) spectroscopy, mass spectrometry, and enzymatic hydrolysis. These, together with standards of **1**, **2**, dinophysistoxin-1 (**3**), and a synthetic specimen of 7-deoxy-**1** (**7**), combined with an understanding of their mass spectrometric fragmentation patterns, were then used to identify **1**–**5**, the 24-*O*-β-d-glucoside of dinophysistoxin-1 (**6**), **7**, 7-deoxy-**2** (**8**), and 7-deoxy-**3** (**9**) in a range of extracts from *Dinophysis* blooms, *Dinophysis* cultures, and contaminated shellfish from Spain, Norway, Ireland, Canada, and New Zealand. A range of *Prorocentrum* *lima* cultures was also examined by liquid chromatography–high resolution tandem mass spectrometry (LC–HRMS/MS) and was found to contain **1**, **3**, **7**, and **9**. However, although **4**–**6** were not detected in these cultures, low levels of putative glycosides with the same exact masses as **4** and **6** were present. The potential implications of these findings for the toxicology, metabolism, and biosynthesis of the okadaic acid group of marine biotoxins are briefly discussed.

## 1. Introduction

**Figure 1:**
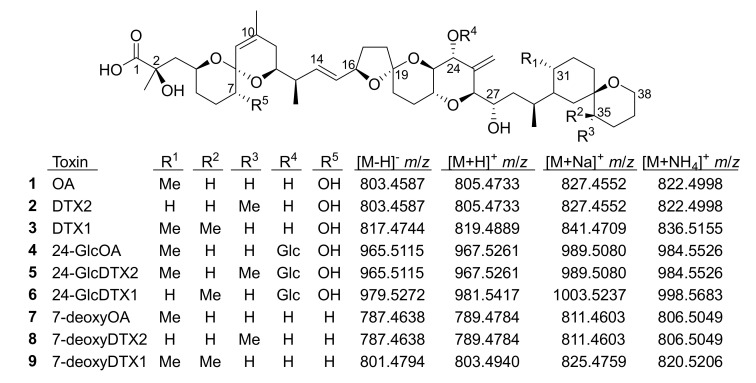
Structures of OA analogues discussed in the text (Glc, 1′-*O*-β-d-glucoside). The *m*/*z* values are the exact masses, and matched the observed accurate mass values to better than ±5 ppm in all cases.

Okadaic acid and dinophysistoxins (OA/DTXs) (e.g., **1**–**3**, Figure 1) are marine polyether toxins associated with diarrhetic shellfish poisoning of human shellfish consumers worldwide. OA/DTXs originate from dinoflagellates of the genera *Prorocentrum* and *Dinophysis* [1,2], and can accumulate in some shellfish species when concentrations of the dinoflagellates are sufficiently high [3]. Consequently, regulatory limits are in place in many countries for **1**–**3** and their esterified forms in shellfish harvested for human consumption [4,5,6]. A range of other OA/DTX derivatives has been identified in dinoflagellates, including esters of **1**–**3** at C-1 [7,8,9], 7-deoxyOA (**7**) [10,11], and 19-epimers of OA and DTX2 [12,13], although the latter appear to be artefacts of extraction and isolation [14]. The metabolism of OA/DTXs in shellfish leads to extensive conversion to 7-*O*-acyl fatty acid esters [15] which, although appearing to be of somewhat reduced toxicity [16], are believed to be largely converted back to the free toxins during digestion [15] and are therefore included in the regulatory framework by including a base-hydrolysis step during sample preparation [4,6].

OA/DTXs are potent inhibitors of protein phosphatases (PPs), especially of PP1 and PP2A, and this is thought to be their primary mode of action [17], although this is not universally accepted [18]. The binding affinity of PPs towards OA/DTXs is modulated by the location and orientation of substituents attached to the cyclic polyether skeleton as well as by modifications of its stereochemistry. For example, esterification at C-1 greatly reduces the binding affinity of PPs, whereas epimerization at C-19 or changes in methylation or stereochemistry at C-31 and C-35 lead to smaller changes in binding affinity [10,13,19]. It should be noted that PP binding affinity is not the only factor that is important for determining the relative toxicity of OA analogues to human consumers, and toxic equivalency factors (TEFs) derived from animal models are typically used to estimate the toxicity of shellfish containing mixtures of OA/DTXs [17,20,21].

Here we report the isolation of 24-*O*-β-d-glucosides of OA and DTX2 (**4** and **5**) from a fraction obtained during the isolation of OA/DTXs and PTXs from *Dinophysis* blooms harvested in Spain and Norway, and their identification by liquid chromatography–high-resolution tandem mass spectrometry (LC–HRMS/MS), nuclear magnetic resonance (NMR) spectroscopy, and enzymatic hydrolysis. We then used these compounds, and a synthetic sample of 7-deoxyOA (**7**) [22], to facilitate identification by LC–HRMS/MS of these and related compounds in cultures of three *Dinophysis* spp., *Prorocentrum lima* cultures, and a selection of Canadian, Irish, Norwegian, and New Zealand shellfish naturally contaminated with OA-group toxins, and in two certified reference materials (NRC CRM-FDMT1 and CRM-DSP-Mus) containing OA-group toxins.

## 2. Results and Discussion

**Figure 2:**
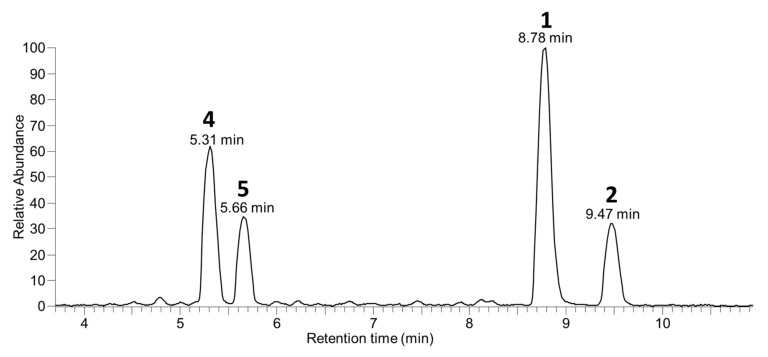
LC–MS^n^ (method A) negative full scan chromatogram of the aqueous fraction remaining after extraction (with diethyl ether and dichloromethane for purification) of the bulk of the **1** and **2** from algae harvested in Spain by Rundberget et al. [24].

**Figure 3:**
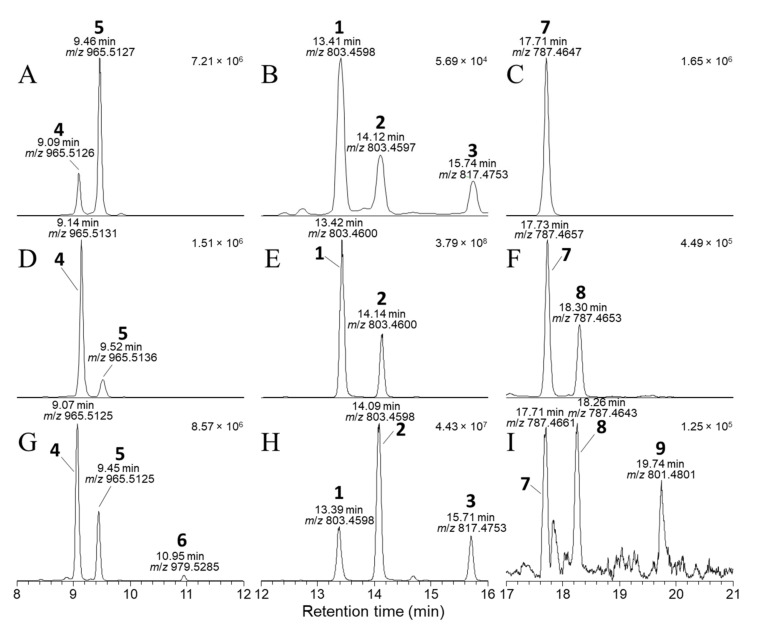
Negative-ionization mode extracted-ion (±5 ppm) SIM chromatograms from LC–HRMS analysis (method B) of: the mixed standard of **4**, **5**, and **7** containing **1**–**3** as minor contaminants (**A**–**C**); an extract of a *D. acuta* culture VGO1065 (**D**–**F**); and the SPE concentrate from CRM-FDMT1 (**G**–**I**). The chromatograms in panels (**A**,**D**,**G**) were extracted at *m*/*z* 965.5115 and 979.5272 (*m*/*z* of [M−H]^−^ for **4**–**6**); those in panels (**B**,**E**,**H**) at *m*/*z* 803.4587 and 817.4744 (*m*/*z* of [M−H]^−^ for **1**–**3**); and in panels (**C**,**F**,**I**) at *m*/*z* 787.4638 and 801.4794 (*m*/*z* of [M−H]^−^ for **7**–**9**). Peaks are labelled with the compound numbers (Figure 1), observed retention times, and *m*/*z* values, and the number in the top right-hand corner of each panel indicates the intensity (counts) of the highest peak. A corresponding set of peaks was also observed in positive-ionization chromatograms (e.g., Figure S2).

It has for several years been the practice in our laboratory to retain residual aqueous extracts following the recovery of known target algal toxins such as OA (**1**), DTX-2 (**2**), DTX-1 (**3**), pectenotoxins, yessotoxins, etc., and at a later date to examine them using LC–MS methods to determine the extent to which target algal toxins had been recovered and whether other, possibly new, algal toxins were present in the retained aqueous fractions at levels sufficient for further investigation. During the course of one such examination of some retained aqueous fractions, we detected appreciable levels of two compounds (**4** and **5**) by LC–MS^n^ (Figure 2) in retained Spanish extracts that exhibited MS^n^ fragmentation patterns reminiscent of **1** and **2**. Initial studies were performed with LC–MS^n^ (method A) at unit resolution, but subsequent studies with LC–HRMS/MS were in all cases consistent (within ±5 ppm) with the initial MS^n^ results and are therefore used in the discussion here. In particular, the [M−H]^−^ ions of the two compounds, which eluted earlier than **1** and **2**, had *m*/*z* 965.5115 (C_50_H_77_O_18_^−^) (Table S1), whereas those of **1** and **2** occurred at *m*/*z* 803.4587 (C_44_H_67_O_13_^−^) (Figure 3). The 162.0528 Da (C_6_H_10_O_5_) difference between the respective pairs of [M−H]^−^ ions, combined with the earlier elution time of the two peaks, suggested that the two earlier-eluting peaks might be glycopyranosyl or glycofuranosyl analogues of OA and DTX-2, since the attachment of a glycoside residue such as D-glucose or D-galactose to a substrate, accompanied by the loss of a water molecule to afford a glycosidic linkage, diagnostically results in a 162.0528 Da increase. Consistent with this, we observed that brief treatment with sodium metaperiodate, which rapidly cleaves *vic*-diols such as are present in many glycosides [23], resulted in the rapid disappearance of the LC–HRMS peaks for **4** and **5** but did not significantly affect the intensities of the peaks for **1**, **2**, **7**, and **8** in an extract of Irish mussel HP (Figure S1).

**Figure 4:**
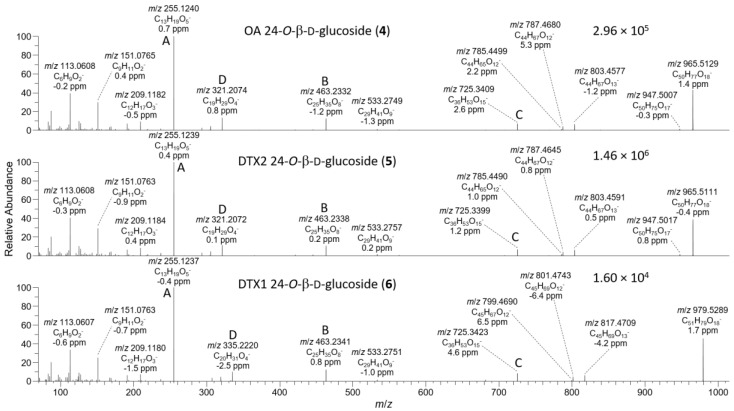
HRMS/MS spectra of [M−H]^−^ for **4**–**6** obtained by LC–HRMS/MS (method B) in negative ionization mode. Spectra of **4** and **5** were from the mixed standard, while the spectrum of **6** was from the Norwegian whole mussel extract. Spectra of **1**–**3** obtained under identical conditions are shown in Figures S3 and S4, and corresponding spectra of **7**–**9** are shown in Figure 7. Spectra of **4**–**6** in positive ionization mode are shown in Figure 5. Ions marked with letters refer to major structurally diagnostic ions listed in Table 1, with additional information in the Supplementary Materials.

**Figure 5:**
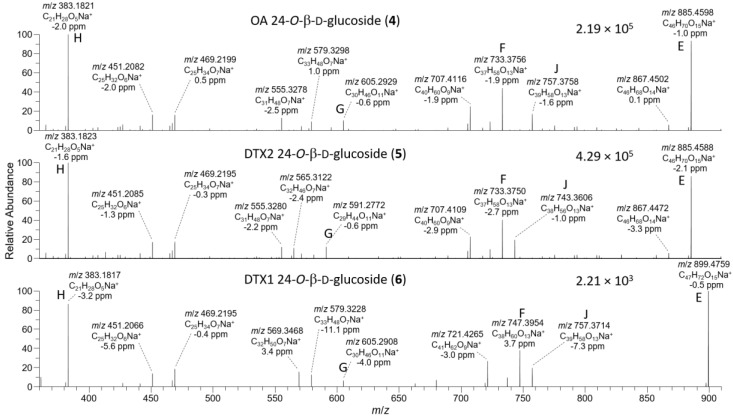
HRMS/MS spectra of [M+Na]^+^ for **4**–**6** obtained by LC–HRMS/MS (method B) in positive ionization mode. Spectra of **4** and **5** were from the mixed standard, while the spectrum of **6** was from the extract of mussels at Indian Pt., NS, Canada. Spectra of **1**–**3** obtained under identical conditions are shown in Figure S6, and corresponding spectra of **7**–**9** are shown in Figure 8. Spectra of **4**–**6** in negative ionization mode are shown in Figure 4. Ions marked with letters refer to major structurally diagnostic ions listed in Table 1, with additional information in the Supplementary Materials.

Flash chromatography of the aqueous fraction on a C18 column, followed by semi-preparative reverse-phase HPLC, afforded **4** and **5** in sufficient quantity and purity for structure determination by NMR spectroscopy at 600 MHz. Detailed analyses of one- and two-dimensional NMR spectra combined with enzymatic hydrolysis and LC–HRMS/MS data (Figure 3, Figure 4 and Figure 5) were then used to show these to be OA 24-*O*-β-d-glucoside (**4**) and DTX-2 24-*O*-β-d-glucoside (**5**) (Figure 1).

### 2.1. Mass Spectrometric Analysis of Glucosides ***4*** and ***5***

The negative ion MS^2^ and HRMS/MS spectra of purified **4** and **5** were characterized by distinctive *m*/*z* 947.5010 (C_50_H_75_O_17_^−^) and 725.3390 (ion C, C_36_H_53_O_15_^−^) ions (Table 1, Figure 4) attributable to the loss of water and cleavage across the C-26–C-27 bond (Table 1), respectively. These ions are the glycosylated analogues (+162.0528 Da, C_6_H_10_O_5_) of the distinctive MS^2^ *m*/*z* 785.4482 (C_44_H_65_O_12_^−^) and 563.2862 (ion C, C_30_H_43_O_10_^−^) ions (Table 1, Figure S3), respectively, seen in the negative MS^2^ and HRMS/MS spectra of **1** and **2**. A series of product ions was also present at *m*/*z* 803.4587, 787.4638, and 785.4490, corresponding to the overall loss of dehydroglucose, gluconolactone, and glucose, respectively, from [M−H]^−^ (Figure S4). The glycosylated analogue of the *m*/*z* 255 retro-Diels–Alder ions (ion A, Table 1) seen in the negative ion MS^2^ spectra of **1** and **2** (Figures S3 and S5) was not observed in the negative ion MS^2^ spectra of **4** and **5**, but a prominent product ion at *m*/*z* 255.1238 (C_13_H_19_O_5_^−^) was present in the LC–HRMS/MS spectra (Figure 4). MS^3^ fragmentation of the *m*/*z* 787 product ions of **4** and **5** afforded *m*/*z* 463.2337 (C_25_H_35_O_7_^−^) ions for which a structure is tentatively proposed (Table 1 and Figure S5). In each case, MS^4^ fragmentation of this ion afforded *m*/*z* 255 ions (Figure S5), indicating the presence of C-1–C-12 of the OA structure (rings A and B). The foregoing data (Table 1, Figure 4) therefore exclude glycosylation of the hydroxy groups at C-1, C-2, C-7, and C-27, suggesting that glycosylation was probably at C-24.

The MS^2^ spectra of [M+NH_4_]^+^ of **4** and **5** predominantly showed a series of [M−NH_3_−(H_2_O)_n_]^+^ and [M−NH_3_−C_6_H_10_O_5_−(H_2_O)_n_]^+^ ions (Figure S7). These ions, the more intense of which appeared at *m*/*z* 949, 931, 913, 805, 787, 769, and 751, did not offer any insight into the point of attachment of their glycosyl residues.

The [M+Na]^+^ ions of **1** and **2** occurred at *m*/*z* 827.4552, while those of their 24-*O*-β-d-glucosides (**4** and **5**) occurred at *m*/*z* 989.5080 (Table S2) and, in each case, their product ion spectra showed distinctive water losses at *m*/*z* 809.4446 and 971.4975. Product ions attributable to neutral loss the of the 4-carbon side-chain attached at C-4 as 2-hydroxyisobutyric acid (C_4_H_8_O_3_, 104.0473 Da) (ion E, Table 1, Figure 5, Figure S5, and S8) were also observed at *m*/*z* 723.4079 (C_40_H_60_O_10_Na^+^) (for **1** and **2**) and 885.4607 (C_46_H_70_O_15_Na^+^) (for **4** and **5**). Loss of this fragment from **4** and **5** excludes C-1 or C-2 glycosylation but does not differentiate between C-7, C-24, or C-27 glycosylation. Ions observed in the [M+Na]^+^ MS^3^, MS^4^, and HRMS/MS spectra of **4** and **5**, including a glycosylated *m*/*z* 733.3770 (ion F, C_37_H_58_O_13_Na^+^) ion believed to arise from the *m*/*z* 885 ion in MS^n^ via a retro-Diels–Alder cleavage across ring-B of this ion (ion E; Table 1, Figure 5 and Figure S8), suggested 24- or 27-*O*-glycosylation, rather than 7-*O*-glycosylation. MS^3^ fragmentation of the *m*/*z* 885 product ions of **4** and **5** afforded product ions at *m*/*z* 757.3770 (C_39_H_58_O_13_Na^+^) and 743.3613 (C_38_H_56_O_13_Na^+^), respectively, attributable to cleavage across the C-31–C-32 and C-30–30-*O*-ether bonds of **4** and **5**, as depicted in Table 1 and Table S2, and Figure S8. This cleavage, with loss of either C_7_H_12_O_2_ (128.0837 Da) or C_8_H_14_O_2_ (142.0994 Da), served to distinguish OA 24-*O*-glucoside (**4**) from DTX2 24-*O*-glucoside (**5**). A full account of positive and negative ion MS^2^ and MS^3^ fragmentation of **1** and **2** and the MS^2^, MS^3^, and MS^4^, as well as the HRMS/MS fragmentation of their glucosides (**4** and **5**, respectively), including plots of MS^n^ and HRMS/MS spectra, is included in the Supplementary Materials (Figures S3–S8, Tables S1 and S2). Taken together, the positive and negative ion MS^n^ and HRMS/MS data in Table 1 strongly supported the 24-OH group as the site of glycosylation.

Other minor compounds were detected by LC–MS^n^ (method A) in the preparative LC fractions, including a pair of compounds which exhibited [M−H]^−^ ions at *m*/*z* 1127.6, with [M+NH_4_]^+^ ions at *m*/*z* 1146.6 (Figure S9). This suggests the presence of diglycosylated (addition of 2 × 162 Da) analogues of **4** and **5**. The [M+NH_4_]^+^ MS^2^ spectra of the pair of diglycosylated analogues showed two sequential 162 Da losses attributable to the loss of dehydrated glycosyl residues, consistent with the presence of two glycosyl residues in these compounds (Figure S9). Unfortunately, the putative diglucosides were not detectable in the samples available for LC–HRMS/MS analysis, and we were not able to establish the structures for these based on the available MS data alone.

### 2.2. NMR Structure Elucidation of Glucosides ***4*** and ***5***

Although some impurities were present in the NMR spectra of **4** and **5**, due to the small amounts of the target compounds (~40 µg) and the risk of significant losses associated with further purification, the decision was made to undertake structure elucidation using the available material with the aid of extensive 2D and selective 1D techniques (Figures S10–S32). ^1^H and ^13^C NMR shifts were determined in CD_3_OD. ^1^H–^1^H connectivities were established in 2D-COSY and -TOCSY experiments performed with mixing times of 80 and 160 ms. ^1^H and ^13^C chemical shifts were correlated in ^1^H-detected HSQC and HMBC experiments. Because the quantity of the isolated glucosides **4** and **5** was insufficient to obtain their ^13^C and DEPT135 spectra directly, ^13^C chemical shifts were established indirectly from the HSQC and HMBC experiments.

A comparison of signal assignments established from detailed analyses of this data for **4** and **5** with the NMR signal assignments determined previously [19] for **1** and **2** revealed that, while the assignments of the C-1 to C-21 signals and the associated proton resonances of **1** and **2** were nearly identical to those established for **4** and **5**, there were marked differences in the resonances of some of the protons and carbons in the vicinity of C-24 (Table 2). For example, the H-24 and C-24 resonances of **1** occurred at 4.07 ppm and 72.1 ppm, respectively, compared to 4.44 ppm and 75.9 ppm, respectively, in **4**. Furthermore, the chemical shifts of the H-22 (3.63 ppm), H-23 (3.42 ppm), and 25 =CH_2_ methylene protons (5.04 and 5.38 ppm) and C-23 (78.3 ppm), C-25 (147.1 ppm), and 25 =*C*H_2_ (112.6 ppm) of **1** differed appreciably from those observed for the H-22 (3.72 ppm), H-23 (3.59 ppm), 25 =CH_2_ methylene protons (5.11 and 5.67 ppm), C-23 (76.9 ppm), C-25 (143.9 ppm), and 25 =*C*H_2_ (114.6 ppm) of **4**. A similar series of variations was observed for corresponding signals of **2** and **5** (Table 2). The ^13^C and ^1^H NMR chemical shifts associated with C-26 and C-28–C-38 of **1** and **4** were essentially identical, as were the corresponding resonances of **2** and **5**, with relatively minor changes in the chemical shifts of H-27 being attributable to glycosylation at the nearby 24-position. These data are consistent with glycosylation at C-24, rather than conjugation being at C-1 and/or C-7, as has hitherto been found for a variety of fatty acid and diol esters of **1** and **2** [7,9,25,26].

Minor differences between the H-3, H-12 and H-14, C-1, C-3, C-4, C-9, and C-10 chemical shifts of **2** and **5**, but not of **1** and **4** (Table 2), can be attributed to the acquisition of the NMR data from the carboxylate salts of **1**, **4**, and **5** compared to the free acid form of **2**, since formic acid treatment was used in the final purification step during our prior isolation of the specimen of **2** [19] but not of **1**, which was purchased as the Na salt. Molecular modeling [19] and X-ray crystallographic data [27,28] were consistent with the folding of **1** and **2** such that the CO_2_H or CO_2_^−^ groups of **1** and its analogues are orientated towards H-12 and H-14.

The presence of glucopyranosyl residues in **4** and **5** were established in a series of 1D-selective TOCSY NMR experiments performed with mixing times of 15, 30, 50, 80, and 160 ms, which sequentially elucidated the chemical shifts and couplings constants of the H-1′ to H-6′ protons (Table 3 and Figures S13 and S25). In particular, it was apparent from the large ^3^*J* couplings (*J*_1′–2′_ = 7.8 ± 0.1 Hz, *J*_2′–3′_ = *J*_3′–4′_ = *J*_4′–5′_ = 9.0 ± 0.3 Hz) that, assuming the presence of a D-glucopyranosyl residue rather than an L-glucopyranosyl residue, H-1′ was β-orientated and that each of the H-1′, H-2′, H-3′, and H-4′ protons was trans-diaxially coupled to neighboring protons. The identity of the saccharide moiety as β-d-glucose was supported by enzymatic hydrolysis of **4** with a commercially available plant β-glucosidase. Although enzymatic hydrolysis only proceeded slowly (39% in 8 h), complete conversion of **4** into **1** was observed by LC–MS^n^ (method A) by 48 h.

**Figure 6:**
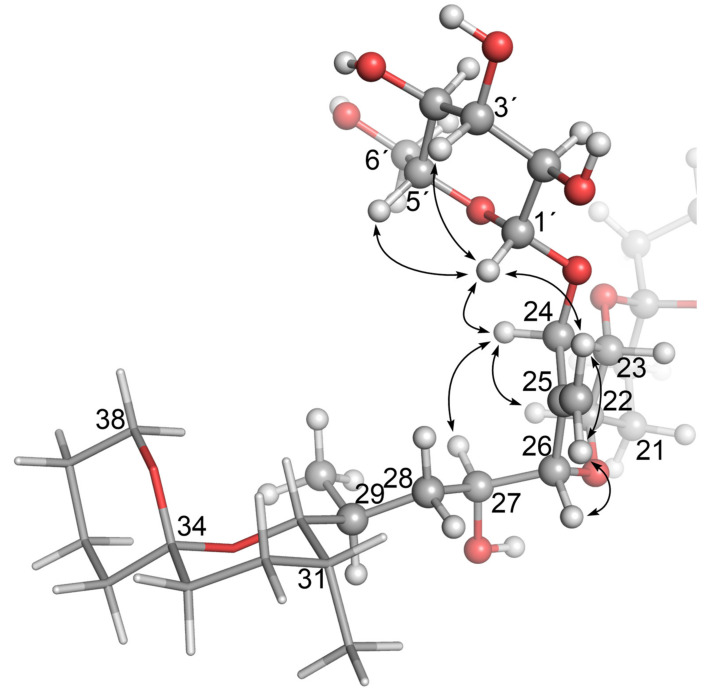
Molecular model of OA 24-*O*-β-d-glucoside (**4**) based on the modelled structure of **1** that Larsen et al. [19] derived from the X-ray crystal structure of Tachibana et al. [28], showing the glucoside-containing region. The arrows show structurally diagnostic through-space correlations relating to the glucosyl moiety observed in SELROESY NMR experiments. Atom numbering is as per Figure 1, with NMR assignments in Table 2 and Table 3. Rings F and G are shown with sticks, and C-1–C-17 are omitted for clarity.

Confirmation that the H-3′ and H-5′ glycosidic protons were axially oriented with respect to H-1′, and that **4** and **5** were glucosylated at C-24, was obtained in 1D-selective ROESY experiments. In the case of **4**, ROESY correlations (Figures S17–S20) were observed between H-1′ (4.39 ppm) and H-3′ (3.35 ppm), H-5′ (3.20 ppm) and H-24 (4.44 ppm), respectively, and to one of the 25 =CH_2_ protons (H*_Z_*, 5.67 ppm). These and other structurally significant ROESY correlations exhibited by H-24, 25 =CH_2_, H-26, and H-27 are shown in Figure 6. An equivalent series of ROESY correlations was observed for **5** (Figures S28–S32). The ROESY correlations observed for **4** also unambiguously established the position of the glycosidic linkage to be the 24-OH of **1**. In particular, **4** showed ROESY correlations between H-1′ and both the H-24 and the *Z*-proton of the olefinic methylene at C-25, while the *E*-proton of the olefinic methylene correlated with H-26 (Figure 6 and Figures S16–S20). Thus, the structures of **4** and **5** are confirmed by NMR spectroscopy and are in complete agreement with the findings from the LC–MS^n^ and LC–HRMS/MS studies of these compounds.

### 2.3. LC–MS^n^ and LC–HRMS/MS Detection of DTX-1 24-O-β-d-Glucoside (***6***)

The detection of **4** and **5** in the Spanish extracts prompted a search for the corresponding glucosylated DTX-1 analogue (**6**) in retained aqueous extracts of samples that we previously had found to contain significant levels of DTX1 (**3**). LC–MS^n^ analyses showed that a compound with the expected characteristics was present at a low level, along with a higher level of **3**, in the corresponding aqueous fraction obtained after pumping a *Dinophysis* bloom at Sogndal, Norway, in November 2005. The positive and negative ion MS^2^ and MS^3^ fragmentation of **6** was similar to those of **4** and **5**, other than a 14 Da increase in the mass of some of the product ions due to the presence of methyl groups at both C-31 and C-35 in **3**, compared to one methyl group at either C-31 or C-35 in **4** and **5** (Figure 1). The negative ion MS^2^ [M−H]^−^ spectrum of **6** included a glycosylated (presumably glucosylated) ion at *m*/*z* 725, whereas the equivalent non-glycosylated ion of **3** occurred at *m*/*z* 563. MS^3^ fragmentation of the *m*/*z* 463 product ion from *m*/*z* 979 MS^2^ ([M−H]^−^) in the spectrum of **6** afforded an *m*/*z* 255 ion, as was also observed in the *m*/*z* 817 MS^2^ ([M−H]^−^) spectrum of **3** and in the *m*/*z* 965–›463 MS^3^ [M−H]^−^ spectra of **4** and **5**. The positive [M+Na]^+^ ion MS^n^ spectra of **3** and **6** included product ions at *m*/*z* 737 and 899, respectively, attributable to the loss of their C-4 side chains as 2-hydroxyisobutyric acid.

Because LC–HRMS/MS was not available in our laboratories at the time that this extract was available, we sought to confirm the identification of **6** by LC–HRMS/MS analysis of extracts of shellfish naturally contaminated with **3** from *Dinophysis* blooms (Tables S1 and S2). CRM-FDMT1 is a freeze-dried certified reference material that contains Norwegian mussels as its sole source of **3** [29,30], and was recently reported to contain low levels of **4** and **5** and a compound with some of its mass spectral characteristics consistent with those expected for **6** [31]. We therefore undertook a detailed LC–HRMS/MS analysis of an SPE-concentrated extract of FDMT1 (Figure 3 and Figure S2), as well as of several extracts of shellfish naturally contaminated with **3** harvested in Atlantic Canada and Norway (Figure 4, Tables S1 and S2), in both positive and negative ionization modes, to determine whether the characteristics of this compound paralleled those of **4** and **5** and were fully consistent with **6**. We found that the negative ionization HRMS/MS spectrum of **6** ([M−H]^−^, *m*/*z* 979.5285, Δ 1.3 ppm for C_51_H_79_O_18_^−^) was essentially identical to those of **4** and **5** in terms of *m*/*z* and relative intensity, except that product ions containing the F- and G-rings (at *m*/*z* 817.4744, 801.4795, 799.4638, and 335.2228 in **6**) were heavier by 14.0157 (one methylene group) than the corresponding product ions from **4** and **5** (Table 1, Figure 4, Figures S3, and S4). Corresponding results were obtained for HRMS/MS spectra of the sodium adduct ion of **6** (Table 1, Figure 5 and Figures S6). The fragmentation patterns indicate glycosylation at 24-OH and the presence of one methyl group at C-31 and a second methyl group in the terminal G-ring (Table 1). These results, together with the fact that **6** usually co-occurred with **4** in naturally contaminated mussels (and in a similar ratio to that of **3**:**1** in the same samples) (Table 4), and the retention time of **6** relative to those of **4** and **5** (Figure 3), indicate that this compound is the 24-*O*-β-d-glucoside of DTX1 (**6**) (Figure 1).

### 2.4. Detection of OA/DTX Analogues in Dinophysis and P. lima Cultures

The finding of glucosides **4**–**6** in algal bloom samples prompted a search for the presence of these compounds by LC–HRMS/MS in unialgal cultures of dinoflagellates known to produce **1**–**3**. Furthermore, as an authentic standard of 7-deoxyOA (**7**) was available [22], this study was extended to evaluate the possible presence of **7**, 7-deoxyDTX2 (**8**) and 7-deoxyDTX1 (**9**) in these cultures. It should be noted that the concentrations of **1**–**9** are reported on the basis of weight in Table 4, Table 5 and Table 6, Tables S3 and S4. However, on a molar basis, the relative concentrations of **4**–**6** are 20% lower, and those of **7**–**9** are 2% higher, when compared to **1**–**3** in the same sample, due to molecular weight differences. The concentrations obtained for **4**–**9** should only be considered indicative because quantitative standards were not available, and significant variations in response factors and matrix effects for these analogues can be expected relative to **1**–**3**.

Six extracts obtained using HP-20 resin were available from previous studies of cultured strains of *D. acuta*, *D. acuminata* (2 strains), and *D. caudata* isolated from Galicia, Spain (Table 4 and Table S5, Figure S33). The *D. acuta* and *D. caudata* (trace amounts only) produced **1** and **2** in a ~3:1 ratio, while the *D. acuminata* strains produced **1** but no detectable **2**. The *D. acuta* extract also contained **4** and **5**, identical by retention time (Figure 3) and HRMS/MS to the standards isolated and identified by NMR spectroscopy, but the abundance of **4** and **5** was ~0.2% relative to **1** and **2**. Similarly, *D. acuminata* contained **4** but at about 0.3% of the abundance of **1**. The *D. caudata* extracts did not contain **4** or **5** when analysed directly, probably because they contained much lower concentrations of **1** and **2** (~100-fold lower than for the *D. acuta* and *D. acuminata* extracts); however, traces of **4** were detected when the extracts were concentrated 10-fold (Table 4, Figure S33).

**Figure 7:**
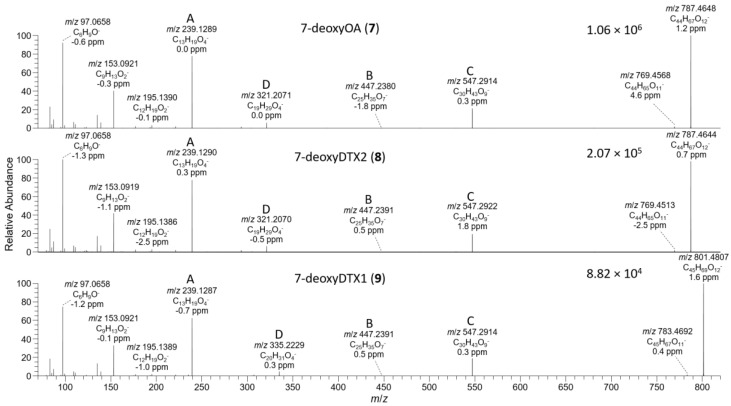
HRMS/MS spectra of [M−H]^−^ for **7**–**9** obtained by LC–HRMS/MS (method B) in negative ionization mode. The spectrum of **7** was from the mixed standard; the spectrum of **8** was from the extract of the *D. acuta* culture; and the spectrum of **9** was from an extract of *P. lima* JHPL2. Spectra of **1**–**3** obtained under identical conditions are shown in Figures S3 and S4, and corresponding spectra of **4**–**6** are shown in Figure 4. Spectra of **7**–**9** in positive ionization mode are shown in Figure 8. Ions marked with letters refer to major structurally diagnostic ions listed in Table 1, with additional information in the Supplementary Material.

**Figure 8:**
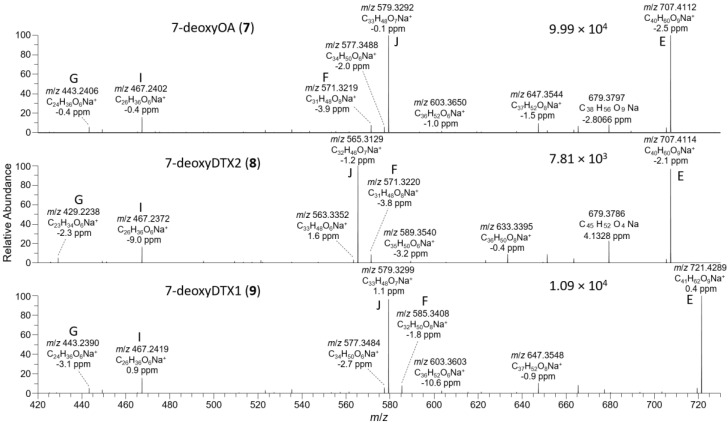
HRMS/MS spectra of [M+Na]^+^ for **7**–**9** obtained by LC–HRMS/MS (method B) in positive ionization mode. The spectrum of **7** was from the mixed standard; the spectrum of **8** was from the *D. acuta* culture; and the spectrum of **9** was from an extract of *P. lima* JHPL2. Spectra of **1**–**3** obtained under identical conditions are shown in Figure S6, and corresponding spectra of **4**–**6** are shown in Figure 5. Spectra of **7**–**9** in negative ionization mode are shown in Figure 7. Ions marked with letters refer to major structurally diagnostic ions listed in Table 1, with additional information in the Supplementary Material.

The *D. acuta* extract contained a later-eluting peak with identical retention time and HRMS and HRMS/MS spectra to the authentic specimen of 7-deoxyOA (**7**) (Table 1, Figure 3, Figure 7, and Figure 8). A characteristic of the product ion spectrum of **7** is that all fragments containing the A-ring are 15.9949 Da lower in mass than the corresponding product ions from **1** (Table 1, Tables S1 and S2, Figure S3), including a prominent negative product ion at *m*/*z* 239.1289 (ion A, Table 1) instead of *m*/*z* 255.1238 as in **1**–**6**. A second, later-eluting peak (**8**, Figure 3F) was also present with an identical negative HRMS/MS spectrum (Figure 7), with approximately one third the intensity of **7** (approximately the same as the ratio of **1**:**2** in this extract). Examination of the product ion spectrum of **8** obtained from [M+Na]^+^ (Figure 8) was also consistent with deoxygenation of an OA-type skeleton at C-7, but the ions at *m*/*z* 429.2248 and 565.3136 (ions G and J in Table 1) indicated the absence of a 31-Me group and the presence of a methyl group in the G-ring. These data are consistent with 7-deoxyDTX2 (**8**), which would also be expected based on biosynthetic considerations. The intensities of the peaks for **7** and **8** were a little over 0.1% of the intensities of the peaks for **1** and **2** in the same sample (Figure 3, Table 4). Similarly, **7** (but not **8**) was detected in the *D. acuminata* extracts, again at a little over 0.1% of the intensity of **1** (Table 4, Figure S33). However, no **7** or **8** was detected in the *D. caudata* extracts, but as with the absence of detectable **4** and **5** in *D. caudata*, this may have been due to the very low levels of **1** and **2** detected in these extracts.

In this study, no cultured strains of *Dinophysis* were available that produced significant amounts of DTX1 (**3**); however, *P. lima* is well known as a producer of **1** and/or **3**. We therefore examined eight strains of *P. lima* for the presence of **1**–**9** (Table 4). Although all of the *P. lima* strains examined produced **1** and/or **3** (but not **2**), 24-*O*-β-d-glucosides **4**–**6** were not detected by LC–HRMS in any of these strains. Early-eluting peaks with the same accurate mass as **4** and **6** were detected, but their retentions times and product ion spectra differed from those of **4** and **6**. For example, the putative DTX1 glycoside in *P. lima* displayed product ions A, B, D–G, and J (but not ions C or H) from Table 1, consistent with glycosylation at either the 24- or 27-hydroxy groups of **1**. Possibly a different hexose is involved, or glucose is conjugated at the 27-OH rather than at the 24-OH as in **4**–**6**, and work is now underway to identify the glycosides of **1** and **3** detected in *P. lima*.

*P. lima* strains JHPL2 (Figure S34) and JHPL3, which produced mainly **3** in addition to much lower amounts of **1**, contained a small amount of **7** along with a much larger peak in negative mode with *m*/*z* 801.4821 (Δ 3.3 ppm for C_45_H_69_O_12_^−^) that displayed product ions consistent with 7-deoxyDTX1 (**9**) (Figure 7, Table 1 and Table S1). In particular, product ions in negative and positive modes were indicative of deoxygenation at C-7 (ions A–J, Table 1) and the presence of a methyl group at C-31 as well as in ring-G (ions C, D, F, H, and I, Table 1). This, together with its retention time and intensity relative to **7**, was consistent with **9**. Compound **9** was detected in all *P. lima* strains that produced **3**, at levels ~0.5% those of **3** (Table 4). The retention time of **9** in the *P. lima* samples was identical to that of the putative **9** present in the CRM-FDMT1 SPE concentrate (Figure S34).

### 2.5. Detection of ***1***–***9*** in Blue Mussels from Canada, Ireland, Norway, and New Zealand

The identification of **4**–**9** in algal blooms, and cultures of *Dinophysis* or *P. lima*, from Canada, Norway, and Spain, suggested that these compounds might be more widely distributed, given the widespread international occurrence of **1**–**3**. This prompted an examination of shellfish samples known to contain **1**–**3**. A recent study [31] suggested that an SPE-concentrated extract of NRC CRM-FDMT1, a composite freeze-dried reference material containing **1**–**3** from mussels harvested in Ireland and Norway [30], also appeared to contain compounds with properties consistent with **4**–**9**. A comparison of retention times, accurate masses (Figure S35), and HRMS/MS spectra of these compounds in the sample, using LC–HRMS/MS (method B), with those obtained in the studies described above was entirely consistent with the presence of low levels of **4**–**9**, together with much higher levels of **1**–**3**. The content of **1**–**3** in CRM-FDMT1 is certified [32], and further confirmation of the identities of the other OA/DTXs in this study was obtained by analysis with LC–HRMS/MS (method C), and by spiking the CRM-FDMT1 SPE-concentrate with **4** and **5** purified from the *Dinophysis* bloom and with synthetic **7** (Figure S36). This showed identical retention times, *m*/*z* for [M−H]^−^, and HRMS/MS spectra for **4**, **5**, and **7** in CRM-FDMT1 and the authentic standards (Figures S37–S40). Furthermore, the elution order, relative intensities, *m*/*z* of [M−H]^−^, and HRMS/MS spectra for **1**–**8** obtained with LC–HRMS/MS (method C) for the CRM-FDMT SPE-concentrate were essentially identical to those that had been obtained with LC–HRMS/MS (method B). Compounds **4**–**7** were also detectable in NRC CRM-DSP-Mus, together with possible trace levels of **8** and **9** (Table 4, Figure S35).

Analysis with LC–HRMS SIM (method B) showed that the mussel tissues from Norway were dominated by **3**, with low levels of glucosides **4**–**6** detected. Similarly, two samples from Irish mussels contained **1** as the main OA/DTX analogue, but low levels of glucosides, and trace levels of **7**, were detected (Table 4). Three mussel samples from Atlantic Canada were dominated by either **1** or **3**, and were found to contain low levels of the corresponding glucosides **4** and/or **6**, and the DTX1-dominated samples also contained **9** (Table 4). Consistent with this, analysis of a concentrated extract from a SPATT passive sampler deployed at Ship Harbour, NS, Canada, was dominated by **3**, but contained a low level of **1** and trace amounts of the DTX1-analogues **6** and **9** (Table 4). All the samples were also analysed with LC–HRMS method C (Figures S41 and S42), which utilized a different HPLC column, mobile phase, and elution gradient. In all cases, the peaks for **1**–**9** displayed identical retention times across the samples in which they had been found using LC–HRMS (method B), strongly supporting the identifications in the samples.

These findings prompted examination of a range of shellfish species from a wider geographic region, which was made possible by the confirmed presence of **1**–**9** in NRC CRM-DSP-Mus and NRC CRM-FDMT1. Nineteen shellfish samples containing **1**–**3** were selected from the Irish biotoxin monitoring programme in the summer of 2020 (Table 5) for post-analysis investigation. The samples, together with an extract of CRM-FDMT1, had been analysed by LC–HRMS (method D) in MS^e^ full scan mode (*m*/*z* 100–1200), which allowed retrospective examination of the data for **1**–**9** to be performed. Glucosides **4** and **5** were detected in all the *Mytilus edulis* samples, but were not detected in any of the other species examined. The *M. edulis* samples had OA-group toxins in both the hydrolyzed and unhydrolyzed extracts, while the other species (scallops, oysters, and clams; five samples in total) only contained the esterified forms of **1** and **2**. The levels of glucosides **4** and **5** were significant in the Irish mussels, ranging from ~5 to 58% (relative to OA and DTX2) assuming 1:1 relative molar responses in the full-scan mode (Table 5, Figure S43). None of the nineteen samples contained detectable amounts of **3**, its glucoside (**6**), or 7-deoxy-OA/DTXs (**7**–**9**) with this method, although trace levels of **3** and **7** were detected in two of these mussel extracts when they were examined with LC–HRMS (method B) (Table 4), and the percentage of glucosides and the total concentration of OA/DTX analogues were very similar with both LC–HRMS methods B and D.

In a preliminary study [33], an SPE-concentrate of a net haul harvested from a *D. acuminata* bloom in Akaroa Harbour, New Zealand, that had been reported to contain **1** and **3** [34], was found to contain **4** by LC–MS^n^ (method A) in positive and negative ionization modes by direct comparison with an aliquot of the Spanish *Dinophysis* bloom extract used for the isolation of **4** and **5**. To confirm the occurrence of **4**, and investigate the possible occurrence of the related analogues **5**–**9**, 11 naturally-contaminated shellfish samples from the New Zealand non-commercial monitoring programme were analysed for **1**–**9** using LC–MS/MS (method E), with NRC CRM-FDMT1 (Figure S44) and the non-quantitative mixed standard of **4**, **5**, and **7** as references for peak identification. Quantitation was performed in negative ionization assuming the same response factors for **4**–**9** as for their corresponding unmodified analogues (**1**–**3**) (Table 6). Trace peaks of **6** and **7** were observed in positive electrospray ionization, but insufficient signal was observed to identify a peak with negative electrospray ionization (Figures S45 and S46). The relative abundance of **4** observed in the samples was 0.6–5.6% *w*/*w* of that of OA (**1**) after hydrolysis. OA-glucoside (**4**) was only observed in *M. edulis* samples, although the apparent absence of **4**–**9** in other species may be related to the much lower concentrations of **1**–**3** in those samples. The concentration of **4** observed in samples was consistent between the non-hydrolyzed and base-hydrolyzed samples, indicating that the base hydrolysis used routinely for hydrolysis of esterified forms of **1**–**3** is unsuitable for hydrolysis of glucosides **4**–**6** and suggesting that only limited esterification of glucosides **4** and **6** was occurring in these blue mussels.

## 3. Conclusions

The 24-*O*-β-d-glucosides of **1** and **2** (i.e., **4** and **5**) appeared to constitute ~0.5% the total OA/DTXs in the *Dinophysis* cultures examined here (Table 4), with the ratio of **4**:**5** reflecting the ratio of **1**:**2**. No *Dinophysis* strains producing significant amounts of **3** were available for this study, but it seems highly likely that when such strains are examined, they will similarly be found to produce low levels of **6**. None of the *P. lima* strains studied produced detectable levels of glucosides **4**–**6**, but **6** was identified by LC–HRMS/MS in Canadian and Norwegian mussels in which **3** was abundant. In each case, the ratio of **4**:**6** was similar to the ratio of **1**:**3** in these mussels, consistent with a shared algal origin for these compounds in the shellfish. In shellfish where glucosides **4**–**6** were detected, they comprised a much higher, and more variable, proportion of the total OA/DTXs **1**–**9**, ranging ~1.4–58%. This suggests either that glucosides **4**–**6** have longer residence times than their aglycones **1**–**3** in blue mussels, that glucosides might also be metabolites produced in mussels from **1**–**3**, or, less likely, that the shellfish were exposed to *Dinophysis* blooms containing much higher proportions of glucosides **4**–**6** than were present in the *Dinophysis* cultures in Table 4. Five SPATT discs from Ireland (Table S3) and one from Canada (Table 4) were available, and in all cases, the glucosides comprised less than 4% of the total OA/DTXs, although due to the higher polarity of the glucosides, this ratio may not accurately reflect the composition of the *Dinophysis* bloom from which they were obtained.

Current extraction methods are not optimized for the much more water-soluble glucosides of **1**–**3**, nor are the glucosides hydrolyzed by the standard base hydrolysis procedure used to cleave the fatty acid ester metabolites produced from **1**–**3** in shellfish. Furthermore, at a given CE, glucosides **4** and **5** may undergo less efficient retro-Diels–Alder cleavage to give the *m*/*z* 255.1238 product ion in negative mode than do **1**–**3** (Figure S40), which would also result in an underestimation of these glucosides when using LC–MS/MS methods optimized for **1**–**3**. These, together with other factors such as the toxin profile of the local *Dinophysis*, water temperature, time since exposure to the *Dinophysis* bloom, and the genetics and metabolic activity of the local blue mussels, could all contribute to variations in the apparent relative abundance of OA/DTX-glucosides **4**–**6** in blue mussels for different regions and seasons (Table 4, Table 5 and Table 6).

7-DeoxyOA (**7**) has been reported from *P. lima* [11,35], but the results obtained here suggest that 7-deoxyOA/DTXs **7**–**9** are probably produced at low levels by all *Dinophysis* and *Prorocentrum* spp. that also produce **1**–**3**. In the strains examined here (Table 4), the dinoflagellate cultures generally produced **7**–**9** at ~0.1% of the levels of **1**–**3**, and approximately in proportion to the relative abundance of the 7-hydroxylated congeners, although in a few strains a much higher proportion of 7-deoxycongeners was present (Table 4). Only very low levels of 7-deoxyOA/DTXs were present in the shellfish analysed in this study (Table 4, Table 5 and Table 6), despite the fact that fatty acid esterification of the 7-hydroxy group—one of the major metabolic pathways for **1**–**3** in blue mussels—is not possible for these analogues. This may indicate that 7-deoxyOA/DTXs are usually only present at low levels in natural blooms, or that they accumulate less efficiently in shellfish than their 7-hydroxylated congeners **1**–**3**.

In this study, glucosides **4**–**6** were found in blue mussel extracts, *Dinophysis* cultures, or algal blooms (via algal harvesting or SPATT discs) in Spain, Norway, Ireland, Canada, and New Zealand, although generally at low levels. DeoxyOA/DTXs **7**–**9** were similarly found in blue mussels, *Dinophysis* and *P. lima* cultures, and algal blooms in Spain, Norway, Ireland, and Canada, invariably at very low levels except in a few of the *P. lima* cultures. Congeners **4**–**9** were not detected in shellfish species other than blue mussels, although this may have been due to the limited number of samples available for testing and the low levels of contamination with **1**–**3** in those samples. Nevertheless, given that glucosides **4** and **5** were present in all the *Dinophysis* cultures tested, and that 7-deoxyOA/DTXs **7**–**9** were detected in all the *P. lima* and most of the *Dinophysis* cultures tested (Table 4), **4**–**9** can be expected to have a worldwide distribution and have the potential to occur at low to moderate levels wherever shellfish contaminated with **1**–**3** are found.

**Figure 9:**
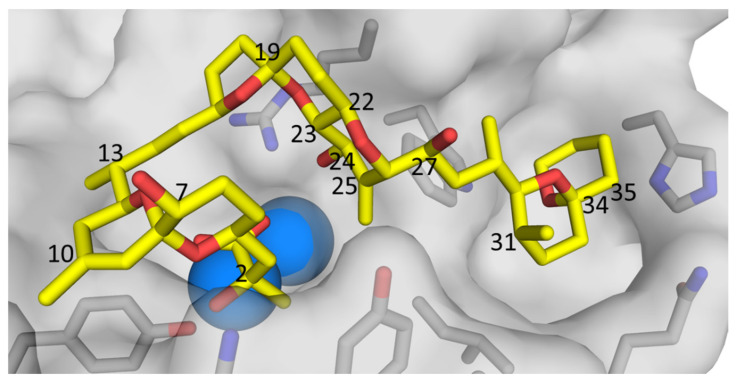
X-ray crystal structure of OA (**1**) (yellow = carbon, red = oxygen) bound to protein phosphatase 2A, generated from the data of Xing et al. [38], showing the protein surface (light grey), catalytic Mn^2+^ ions (blue spheres), and amino acid side-chains (grey = carbon, blue = nitrogen, red = oxygen) adjacent to the binding site. Selected atom numbers for **1** (Figure 1) are marked. Note the orientation of the 24-OH of **1**, pointing directly into the binding site, suggesting that 24-*O*-glucosylation of **1** (i.e., to form **4**) is likely to reduce the affinity of PP2 toward **4**, relative to **1**.

Current regulatory methodology will underestimate the total burden of OA/DTXs in shellfish ingested by consumers. However, the toxicities of analogues **4**–**9** relative to **1**–**3** remain to be determined. Generally, protein phosphatases 1 and 2A (PP1 and PP2A) are considered to be the primary molecular targets of the OA-group of toxins [36]. X-ray crystal structures of **1** bound to PP1 [37], and **1**–**3** bound to PP2A [38,39], indicate that the 24-OH group points directly into the binding site cavity (Figure 9). This suggests that significant disruption to PP-binding, with a consequent reduction in binding affinity, is likely to accompany the addition of a bulky *O*-glucosyl group to the 24-position of **1**–**3**. However, the glucosides could potentially be cleaved by enzymatic, bacterial, and/or acidic hydrolysis during passage through the human digestive tract, in which case the toxicities of **4**–**6** could approach those of aglycones **1**–**3**. The inhibitory potencies of 7-deoxyOA (**7**) toward PP2A and PP1 are approximately half of those of OA (**1**), which is consistent with the orientation of the 7-OH group out and away from the binding sites in PP1 and PP2A (Figure 9). However, with *K*_i_ values of 69 pM and 215 nM for PP2A and PP1, respectively [10], **7** is nonetheless a very potent inhibitor of these enzymes. Given this, and the close structural similarity of **1** and **7**, these compounds can be expected to have similar toxicities in vivo, and the same considerations seem likely to apply to the corresponding pairs of DTXs (**2** and **8**, and **3** and **9**).

Compounds **4**–**6**, **8,** and **9** have not been reported before, and **7** has only been reported from *P. lima*. The present study suggests that **4**–**9** may co-occur routinely with **1**–**3**, but the limited data obtained in this study suggest that the levels of **4**–**9** may be of limited toxicological significance in shellfish. The availability of reference materials containing all of these compounds (**1**–**9**) (Table 4), such as CRM-FDMT1, allows identification of these compounds and an estimation of their levels in a wider array of species and geographical locations by anyone with suitable analytical instrumentation.

## 4. Materials and Methods

### 4.1. Reagents

Solvents (LC–MS grade) for LC–HRMS (method D) were from Labscan (Dublin, Ireland). Solvents and reagents (MeCN, MeOH, formic acid, and ammonium formate) used with liquid chromatography–multi-stage mass spectrometry (LC–MS^n^, method A) were from Rathburn Chemical (Walkerburn, UK) [24]. MeOH for extraction of shellfish, MeCN for LC–MS (both Optima LC–MS grade), from Fisher Scientific (Whitby, ON, Canada), and formic acid (98%) from Honeywell–Fluka (Oakville, ON, Canada) were used with LC–HRMS (methods B, C, and E). Distilled water was further purified using Barnstead nanopure diamond UV (Thermo Scientific, IA, USA) and Milli-Q (Millipore Corp., Billerica, MA, USA) purification systems. Formic acid (≥98%), ammonium formate (>98%), and sodium hydroxide for LC–MS (methods A and D), and β-glucosidase (G4511; 10–30 units/mg, from almonds) were from Sigma–Aldrich (Steinheim, Germany and Oakville, ON, Canada). Diaion HP-20SS resin, 75–150 µm, was from Supelco (Bellefonte, PA, USA). CRMs OA-d, DTX1-b, DTX2-b, DSP-Mus-c, and FDMT1 were from the National Research Council (Halifax, NS, Canada) [12,32]. A pure specimen of synthetic 7-deoxyOA (**7**) was provided by Craig J. Forsyth (The Ohio State University, Columbus, OH, USA) from work performed in collaboration with Amy B. Dounay at the University of Minnesota (Minneapolis, MN, USA) [22]. Specimens of **4** and **5** purified from a *Dinophysis* bloom (see below), and synthetic **7**, were each dissolved in MeOH (1 mL), and their approximate relative concentrations estimated by LC–HRMS (method C). A non-quantitative mixed standard was prepared by mixing aliquots of **4** and **5** with an aliquot of **7**. NRC RM-Multi-toxin, an in-house reference material containing a range of purified lipophilic algal toxins, including OA (271 ng/mL), DTX1 (315 ng/mL), and DTX2 (240 ng/mL) in MeOH, was used for quantitation with LC–HRMS (method B). Quantitative standards of **1**–**3** were used to quantify **1**–**9** in algal and shellfish extracts (Table 4, Table 5 and Table 6, Tables S3 and S4) by assuming identical responses for related sets of analogues (e.g., **1**, **4**, and **7**) and applying a correction to allow for the differences in molecular weights of the glucosides (**4**–**6**) and 7-deoxy-derivatives (**7**–**9**) relative to their unmodified analogues (**1**–**3**).

### 4.2. Shellfish Samples

*Ireland*. OA group-contaminated raw samples, tested as part of the routine monitoring programme in Ireland, were selected for analysis. The shellfish (*M. edulis*, *Cerastoderma edule*, *Crassostreagigas*, and *Pecten maximus* remainder tissue) were shucked and homogenized with a Waring blender before extraction. Tissue samples were weighed (2 g) into 50 mL centrifuge tubes and extracted by vortex-mixing for 1 min with 9 mL of MeOH, centrifuged at 2683× *g* (5 min), and the supernatants were decanted into 25 mL volumetric flasks. This step was repeated, and the supernatants were decanted into the same 25 mL volumetric flasks, which were brought to volume with MeOH. The extracts were filtered through Whatman 0.2 µm cellulose acetate filters into HPLC vials from which 1 mL was pipetted into a separate HPLC vial for hydrolysis (to convert the OA/DTX esters to the parent compounds). Hydrolysis was performed by addition of NaOH (2.5 M; 125 µL), with heating in a water bath (76 °C; 20 min), cooling, and neutralizing with HCl (2.5 M; 125 µL). Both hydrolyzed and unhydrolyzed samples were analysed by LC–HRMS (method D). Existing LC–HRMS data (method B from Kilcoyne et al. [40]) from an extract of hepatopancreas (HP) from AZA-contaminated mussels from Bruckless, with and without periodate treatment, were also re-examined for the presence of **1**–**9**.

*New Zealand*. Eleven shellfish samples identified as naturally contaminated with OA-group toxins in the New Zealand non-commercial testing program were obtained from the positive samples library. Six were blue mussel (*M. edulis*), four were green-lipped mussel (*Perna canaliculus*), and one was tuatua (*Paphies subtriangulata*). The shellfish were shucked and homogenized before extraction. Samples were analysed as per McNabb et al. [41]. Briefly, tissue samples (2 g) were weighed into 50 mL centrifuge tubes and extracted by ultra-homogenization (19,000 rpm, 1 min) with 90% MeOH (18 mL), then centrifuged (3200× *g*, 10 min). An aliquot (2 mL) was extracted with n-hexane (5 mL), and centrifuged at 2000× *g*. A portion of the aqueous–methanolic layer was transferred to a vial for analysis. A 1 mL aliquot of the extract without hexane extraction was transferred to a microcentrifuge tube with an O-ring-sealed screw cap for hydrolysis. Hydrolysis was performed by the addition of NaOH (2.5 M; 125 µL) and heating (76 °C; 40 min), cooling in an ice bath for 5 min, and neutralizing with acetic acid (2.5 M; 125 µL). The extract was then centrifuged (17,000× *g*, 5 min), and an aliquot (200 µL) was diluted with 80% MeOH (600 µL). Both hydrolyzed and unhydrolyzed samples were analysed by LC–MS/MS (method E).

*Canada and Norway*. Frozen (stored −20 °C) mussel tissue samples naturally contaminated with **1**–**3** were available from earlier studies. Whole mussel and excised HP from Flødevigen, Norway, were from a batch previously used in the production of FDMT1 [30]. Whole raw mussels from Bonavista Bay, NF, Canada, harvested in October 1993, were associated with a DSP incident linked to a *Dinophysis norvegica* bloom [42]. Mussels from Ship Harbour, NS, Canada, were harvested in July 2004. Mussels harvested in August 2017 from Indian Pt., NS, Canada, were obtained from the Canadian Food Inspection Agency [43], and the residual liquid “broth” from steaming and draining the shucked mussels, containing ~10% solids, was stored at −20 °C until extraction in the same manner as for mussel tissues. Frozen shucked tissues were extracted in a single dispersive extraction. Pieces of the frozen whole animal tissue (3–4 g) were broken off, weighed, and homogenized with a hand-held Omni Prep homogenizer (Omni International, Kennesaw, GA, USA) using a disposable shearing probe until apparent homogeneity (ca 3 min). The HP tissue had been homogenized prior to freezing, and a portion was taken from thawed material. The broth was decanted into a 50 mL centrifuge tube. For extraction, tissue (~0.8 g) was placed in a 50 mL centrifuge tube with MeOH (4 mL) and homogenized (Omni Prep homogenizer; 3 min, 10,000 rpm). The homogenate was centrifuged (3950 *g*, 10 min, 15 °C), and the supernatant decanted into a 5 mL volumetric flask and brought to volume with MeOH. Prior to analysis, samples were filtered through spin filters (0.45 µm PVDF, Millipore Corp., Billerica, MA, USA) by centrifugation (1400 *g*, 5 min). Extracts of CRM-FDMT1 and CRM-DSP-Mus were prepared according to the liquid–solid extraction method of McCarron et al. [32,44], and a solid-phase extraction (SPE) concentrate of CRM-FDMT1 was prepared as described by Wright and McCarron [31]. The CRM-FDMT1 SPE-concentrate (100 µL) was spiked with the stock solutions of **4**, **5,** and **7** using volumes (~1–2 µL) estimated by LC–HRMS (method C) to add approximately equimolar amounts of each compound.

### 4.3. Passive Sampler Extracts

Extracts of dissolved lipophilic toxins in the water column along the northwest (Bruckless, Donegal) and southern (Bantry Bay) coasts of Ireland were obtained at different depths (surface and 10 m) using passive samplers, with solid-phase adsorption toxin tracking (SPATT). The extracts were generated in the studies described by Fux et al. [45,46], and were stored at −20 °C until analysis by LC–HRMS/MS (method C). Two SPATT extracts taken close together on the same day from a separate study at Ship Harbour, NS, Canada, in 2005, and obtained in the same way, were provided by Nancy I. Lewis (manuscript in preparation). The levels of **4**–**9** in the Canadian SPATT samples were barely detectable (LC–HRMS method B), so aliquots were combined, evaporated under a stream of N_2_, and redissolved in 50% MeOH to increase the concentration 7-fold.

### 4.4. Isolation of Glucosides ***4*** and ***5***

The aqueous fractions were derived from large-scale pumping of filtered *Dinophysis* blooms at Bueu, Ría de Pontevedra, Spain (November 2005), Flødevigen, Norway (October–November 2005), and Sogndal, Norway (November 2005) through columns containing HP-20, and subsequent sequential liquid–liquid partitioning of the HP-20 extracts with diethyl ether and dichloromethane, as described by Rundberget et al. [24]. The combined residual aqueous fractions were applied to a column (20 mm i.d.) packed with LiChroprep RP-18 (40–63 µm, ~10 g; Merck, Darmstadt, Germany). The column had previously been conditioned sequentially with MeOH (400 mL) and MeOH–H_2_O (1:1, 300 mL followed by 1:9, 300 mL). After application of the extract, the column was eluted with MeOH–H_2_O (1:9, 200 mL; 1:4, 200 mL; 3:7, 200 mL; 2:3, 200 mL; and 1:1, 200 mL) and MeOH (300 mL), and 50-mL fractions were collected. Aliquots of each fraction were analysed by LC–MS^n^ (method A). Fractions containing **4** and **5** were combined and evaporated to dryness using a rotary evaporator. The material obtained from three repetitions of the foregoing procedure was combined and taken up in MeOH–H_2_O (9:1, 3 mL). Portions (500 µL) of the resulting solution were applied to a Luna C18 HPLC column (5 µm, 250 mm × 10 mm; Phenomenex, Torrance, CA, USA) interfaced to a Gilson model 321 pump and a model 232 XL injector (Gilson, Middelton, WI, USA) and a model 1100 series G1315A diode array detector (Agilent, Palo Alto, CA, USA). The column was eluted (3 mL/min) isocratically with MeCN–H_2_O (1:4) for 6 min, and then with a gradient to MeCN–H_2_O (3:7) over 60 min, after which the column was flushed with MeCN (100 mL). Fractions (10 mL) were collected, and aliquots of each fraction were analysed by LC–MS^n^. Fractions from 6 repetitions of the semi-preparative LC separation that contained predominantly either OA 24-*O*-β-d-glucoside (**4**) (1st eluting glucoside) or DTX2 24-*O*-β-d-glucoside (**5**) (2nd eluting glycoside), respectively, were each combined and evaporated to dryness using a rotary evaporator, and the resulting residues were freeze-dried to afford ~40 µg of both **4** and **5** for NMR spectroscopy.

An aliquot of a side-fraction from purification that contained glycoside-**4**, but not any detectable **1**–**3** or **5**–**9**, was evaporated to dryness under a stream of dry nitrogen in a vial. β-Glucosidase (1.1 mg, 10–30 units/mg) was dissolved in a 500 µL sodium acetate buffer (0.1 M, pH 5.4), and added to the semi-purified **4**. The mixture was gently vortex-mixed, and the vial was placed in the LC with the tray set to 37 °C and injected periodically to monitor progress of the reaction using LC–MS^n^ (method A).

### 4.5. Dinophysis Cultures

The cultures of *Dinophysis* were all established from water samples from the Galician Rías Baixas, northwest Spain, and were kept at the CCVIEO culture collection at the Instituto Español de Oceanografía. *D. acuta* (VGO1065) and *D. acuminata* (VGO1349) were isolated from Ría de Pontevedra in October 2010 and May 2016, respectively, and *D. caudata* (VGO1396) and *D. acuminata* (VGO1391) from Ría de Vigo in July 2016 and November 2017, respectively. Mixotrophic cultures of *Dinophysis* were grown with the ciliate *Mesodinium rubrum* (AND-A0711) fed with the cryptophyte *Teleaulax amphioxeia* (AND-A0710). Both ciliate and cryptophytes were isolated from water samples from Huelva (southwest Spain) in 2007. The cultures were grown as described previously [47], at 15 °C in diluted (1:20) K_−Si_ medium [48], except *D. caudata* that was grown in diluted (1:20) L1_−Si_ medium [49]. Culture media were prepared with autoclaved seawater at pH 8.0 and a salinity of 32 psu, and cultures were provided with a photon flux of ~150 µmol/m^2^/s of photosynthetically active radiation under a 12 h:12 h light:dark photoperiod. Irradiance was delivered by Osram LED 30W-cold light, 6400 K tubes (OSRAM GmbH, Munich, Germany). All cultures were non-axenic. Extraction of cultures was performed by lysing the cells by the addition of acetone (75 mL/L) to the culture, stirring at low speed for 24 h with activated HP-20SS resin (2 g/L). Recovery of the resin was conducted by filtration (20 µm mesh) and air-drying (50 °C, 3 h) the resin, and the dried resin was stored at −20 °C until warming to ambient temperature for extraction. Extraction of the combined *D. acuta* cultures (total of 9,226,000 cells) and recovery of the resin was performed as above. The resin from the *D. acuta* cultures was extracted by repeated sonication in MeOH for ~0.5 h (~7 × 50 mL and 6 × 100 mL, in total ~950 mL); the extracts were combined, filtered (Whatman filter paper), and evaporated under vacuum, and the residue was dissolved in MeOH (50 mL) for analysis. The resin from *D. acuminata* strain VGO1349 (sample #3, Table S5; 6,331,500 cells) [47] was extracted by repeated sonication in MeOH for ~0.5 h (~3 × 30 mL, in total 100 mL), and the combined extracts were filtered (Whatman filter paper). The resin from *D. caudata* strain VGO1396 (3,252,000 cells) was extracted by repeated sonication in MeOH for ~0.5 h (~5 × 40 mL, in total 200 mL), and the combined extracts were filtered (Whatman filter paper). See Table S5 for details of *Dinophysis* cultures.

### 4.6. Cultured P. lima Extracts

A pellet of *P. lima* (CCMI-1036, isolated from southwest Ireland) was generated in studies carried out by Kilcoyne et al. [14]. The contents of a cell stack (~2 L) were filtered through a 20 µm mesh, and the biomass was transferred into a 50 mL centrifuge tube. An aliquot (0.5 g) was extracted by vortex mixing for 1 min with 9 mL of MeOH and centrifuged at 2683 *g* (5 min), and the supernatant was decanted into a 25 mL volumetric flask. This step was repeated, and the supernatant was decanted into the same 25 mL volumetric flask, which was brought to volume with MeOH. The extract was filtered through a Whatman 0.2 µm cellulose acetate filter into an HPLC vial for analysis by LC-HRMS/MS (method D). A culture of *P. lima* strain PL20V, isolated from Galicia, Spain [50], was grown in a 5 L (Corning cellSTACK, Lowel, MA, USA) culture flask, containing ~2.2 L of L1 culture medium [51] (at 18 °C, photoperiod 12 h:12 h light:dark). *P. lima* strain IP197 was established from single cells isolated from plankton samples collected in coastal waters of Nova Scotia, Canada in August 1990 [52]. *P. lima* CCMP2579 was obtained from the Bigelow National Center for Marine Algae and Microbiota (NCMA, Boothbay, ME, USA). Cultures were grown on L1 medium [51] at 18 °C under a 14 h:10 h light:dark photoperiod with a photon flux of ~60–75 μmol/m^2^/s cool white light. For toxin analysis, cells were concentrated by centrifugation at 2100× *g* for 10 min at 4 °C in 15 mL centrifuge tubes, and the supernatant was reserved for analysis. Pellets were sonicated on ice with 1:1 MeOH–H_2_O (0.5 mL), using an ultrasonicator probe (Q500, Qsonica LLC, Newtown, CT, USA) at 40% amplitude on 2:3 pulse mode for 3 min. Extracts were centrifuged as above, and the supernatant was filtered using 0.45 μm centrifuge cartridges (PVDF Ultrafree-MC-HV). Extracts were stored in amber autosampler vials at −20 °C for analysis.

### 4.7. LC–MS^n^ (Method A)

Liquid chromatography was performed on a Symmetry C18 column (3.5 µm, 50 × 2.1 mm; Waters, Milford, MA, USA), using a Surveyor HPLC system (Thermo Electron Corporation, Waltham, MA, USA). Separation was achieved using mobile phases A and B of H_2_O and MeCN, respectively (both containing 2 mM ammonium formate and 0.01% formic acid). A linear gradient elution (0.25 mL/min) was used from 30 to 100% B over 20 min, held for 10 min, then returned to 30% B. The HPLC system was coupled to an LTQ ion trap mass spectrometer operating with an electrospray ionization (ESI) interface (Thermo Electron Corporation). Typical ESI parameters were spray voltage ±4.5 kV, heated capillary temperature 250 °C, and capillary at 35 or −20 V (in positive or negative modes). The mass spectrometer was operated in scan mode (*m*/*z* 400–1500). LC–MS^n^ experiments were conducted in positive ionization mode with collision energy (CE) 55 eV (**1**–**6**) and in negative ionization mode at CE 35 eV (**1**–**3**) or 40 eV (**4**–**6**). The mass range scanned was 10 units greater than the precursor *m*/*z* down to the lowest *m*/*z* value allowed by the ion trap mass spectrometer.

### 4.8. LC–HRMS (Method B)

LC-HRMS (method B) was conducted with a Q Exactive-HF Orbitrap mass spectrometer equipped with a HESI-II heated electrospray ionization interface (ThermoFisher Scientific, Waltham, MA, USA) with an Agilent 1200 G1312B binary pump, G1367C autosampler (tray set to 10 °C), and G1316B column oven (Agilent Technologies, Palo Alto, CA, USA). Analyses were performed with a Symmetry Shield RP18 column (3.5 µm, 100 × 2.1 mm; Waters, USA) at 40 °C, with mobile phases A and B of H_2_O and MeCN, respectively, each of which contained formic acid (0.1% *v*/*v*). A linear gradient (0.3 mL/min) was used from 20 to 100% B over 20 min, held at 100% B (6 min), then returned to 20% B over 0.1 min and held at 20% B (2.9 min) to equilibrate the column. The flow was diverted to waste for the first 2 min and final 3 min, and the injection volume was typically 1–10 µL depending on the sample and experiment. The mass spectrometer was calibrated from *m*/*z* 74–1622 and *m*/*z* 69–1780 in positive and negative ion modes, respectively; the spray voltage was ±3.7 kV; the capillary temperature was 350 °C; and the sheath and auxiliary gas flow rates were 25 and 8 units, respectively, with MS data acquired from 2 to 27 min. Initial screening was performed in full-scan (FS) in positive and negative switching mode at *m*/*z* 500–2000, with the resolution setting 60,000, AGC target 1 × 10^6^, and maximum injection time (max IT) 120 ms. Subsequent targeted selected ion monitoring (SIM) was performed in negative mode using the same parameters except for an AGC target of 5 × 10^5^ and max IT 1000 ms, at *m*/*z* 947.5–997.5 from 8 to 11.5 min and at *m*/*z* 786–836 from 11.5 to 22 min. Quantitation (Table 4) was performed by extracting exact masses of the [M−H]^−^ ions (Figure 1, ±5 ppm). MS/MS spectra were obtained using alternating FS (obtained as described above) and parallel reaction monitoring (PRM), in either positive or negative mode. Scheduled PRM was performed with an inclusion list, selection width set to 0.4 Da, the resolution set to 15,000, max IT 3000 ms, and AGC target of 2 × 10^5^, and a stepped CE of 50, 65, and 80 eV in negative mode ([M−H]^−^). In positive mode ([M+Na]^+^), scheduled PRM was performed with the same settings except that the isolation width was set to 0.7 Da, and the stepped CE was 80, 90, and 100 eV. Data were processed using Xcalibur v. 4.0 (Thermo Fisher Scientific).

### 4.9. LC–HRMS (Method C)

LC–HRMS (method C) was conducted with the same pump, autosampler, and mass spectrometer as for LC–HRMS method B. An Agilent Poroshell 120 SB-C18 column (2.7 µm, 150 × 2.1 mm) was held at 40 °C and eluted (0.275 mL/min) with mobile phases consisting of H_2_O (A) and 95% MeCN (B), each containing 50 mM formic acid and 2 mM ammonium formate. The gradient elution was from 5% to 100% B over 20 min, then held for 25 min before re-equilibration (10 min), with <5 µL injections. The mobile phase was diverted to waste for the first 6 min of the run and during re-equilibration of the column. Source conditions for the mass spectrometer, run in negative mode and calibrated as described for LC–HRMS method B, were: spray voltage 2.7 kV, capillary temperature 350 °C, sheath gas 40, auxiliary gas 15 (arbitrary units) with the probe heater temperature set to 300 °C, and S-Lens RF Level 50. Full scan (FS)**:** used the 60,000 resolution setting, 1 × 10^6^ AGC target, and max IT 200 ms at *m*/*z* 500–1600; FS with parallel reaction monitoring (PRM)**:** FS settings were as for FS (above), with PRM used for targeted MS/MS collection using an isolation window of 0.7 Da on precursor masses and spectra acquired with the 15,000 resolution setting, AGC target 2 × 10^5^, max IT 150 ms, and CE 70 eV. FS with all ion fragmentation (AIF): FS settings were as above except that the resolution setting was 120,000, max IT 100 ms, and the scan range was *m*/*z* 700–1200. AIF scans were obtained in negative mode using the 30,000 resolution setting, AGC target 1 × 10^6^, max IT 300 ms, stepped CE of 60 and 80, and scanned at *m*/*z* 80–1200. Alternating FS and AIF scans were acquired, and quantitation of shellfish extracts (Table S4) was performed by extracting exact masses of the [M−H]^−^ ions (Figure 1, ±5 ppm) from the negative FS chromatograms.

### 4.10. LC–HRMS (Method D)

Analysis was performed using a Waters Acquity UPLC coupled to a Xevo G2-S QToF monitoring in MS^e^ mode (negative ionization, *m*/*z* 100–1200) using leucine enkephalin as the reference compound, selecting masses for OA/DTX2 (**1** and **2**), *m*/*z* 803.45; OA/DTX2 glucosides **4** and **5**, *m*/*z* 965.51; and 7-deoxyOA/DTX2 (**7**), 787.46. The cone voltage was 40 V; CE was 50 eV; the cone and desolvation gas flows were set at 0 and 600 L/h, respectively; and the source temperature was 120 °C. Chromatography was performed with an Acquity UPLC BEH C18 column (1.7 µm, 50 × 2.1 mm; Waters, Wexford, Ireland). Binary gradient elution was used, with mobile phase A consisting of H_2_O and mobile phase B of MeCN (95%) in H_2_O (both containing 2 mM ammonium formate and 50 mM formic acid). The gradient was from 5 to 90% B over 2 min at 0.3 mL/min, held for 1 min, and returned to the initial conditions and held for 1 min to equilibrate the system (total run time 4 min). The injection volume was 2 µL, and the column and sample temperatures were 25 °C and 6 °C, respectively.

### 4.11. LC–MS/MS (Method E)

LC–MS/MS was conducted with a 6500+ QTRAP tandem quadrupole mass spectrometer coupled with an Exion LC composed of two AD pump modules each with low pressure proportioning valves, a multiplate autosampler, an AC column oven with a two-position six-port column selection valve, and a wide pH compatibility kit (all Sciex, Framingham, MA, USA). Analysis was performed in both negative and positive electrospray ionization with an ionization voltage −4500 V and 5500 V, respectively. Curtain gas was set at 30; collision gas was set to high; temperature was set to 550 °C; and ion source gases 1 and 2 were set at 50 and 55, respectively. The declustering potential was set to 90 V in positive ion mode, and −80 V in negative ion mode. Selected multiple reaction monitoring (MRM) transitions (numbers in parentheses indicate CE (eV)) in negative ion mode were: 803.5🡒255.1 (63) and 563.3 (57) for OA/DTX2 (**1** and **2**); 817.5🡒255.1 (63) and 563.3 (57) for DTX1 (**3**); 965.5🡒255.1 (63) and 725.3 (57) for OA/DTX2 glucosides **4** and **5**; 979.5🡒255.1 (63) and 725.3 (57) for DTX1 glucoside **6**; 787.5🡒239.1 (63) and 547.2 (57) for 7-deoxyOA/DTX2 (**7** and **8**); and 801.5🡒239.1 (63) and 547.2 (57) for 7-deoxyDTX1 (**9**) (Table S6). Selected MRM transitions in positive ion mode were: 827.6🡒723.5 (65) for OA/DTX2 (**1** and **2**); 841.5🡒737.4 (65) for DTX1 (**3**); 989.5🡒885.5 (65) for OA/DTX2 glucosides **4** and **5**; 1003.5🡒899.4 (65) for DTX1 glucoside **6**; 811.6🡒707.5 (65) for 7-deoxy-OA/DTX2 (**7** and **8**); and 825.5🡒721.4 (65) for 7-deoxyDTX1 (**9**) (Table S6). Chromatography was performed with an Acquity UPLC BEH Shield RP18 column (1.7 µm, 50 × 2.1 mm; Waters, Ireland). Binary gradient elution was used, with mobile phase A consisting of 5% MeCN in H_2_O, and mobile phase B of 95% MeCN (each containing 50 mM formic acid and 2.53 mM NH_4_OH). The gradient (0.5 mL/min) started at 0% B, held for 0.1 min, then increased linearly over 0.3 min to 15% B, then to 30% B over 0.5 min, to 80% B over 4 min, then to 100% B over 0.01 min and held for 1.49 min before returning to 0% B over 0.01 min and held for 0.6 min (total run time 7.01 min) to equilibrate the system. The injection volume was 2 µL, and the column and sample temperatures were 40 °C and 4 °C, respectively.

### 4.12. NMR Spectroscopy

NMR spectroscopy was performed using Avance I and Avance II 600 MHz spectrometers (Bruker, Fallanden, Switzerland) equipped with TCI cryoprobes and Z-gradient coils, at 30 °C. Structures were determined by analysis of ^1^H, correlation spectroscopy (COSY), total correlated spectroscopy (TOCSY), nuclear Overhauser effect spectroscopy (NOESY), rotational nuclear Overhauser effect spectroscopy (ROESY), heteronuclear single quantum correlation (HSQC), heteronuclear multiple bond coherence (HMBC), and a series of 1D selective TOCSY (SELTOCSY) and ROESY (SELROESY) NMR spectra. Samples of **4** and **5** were dissolved in ~0.5 mL CD_3_OD at 30 °C, and chemical shifts were referenced to internal C*H*D_2_OD (3.31 ppm) or *C*D_3_OD (49.0 ppm). Single- or double-frequency pre-saturation of solvent resonances was performed using continuous wave pre-saturation, as required. After completion of the NMR studies, **4** and **5** were transferred to vials and stored at −20 °C until required for LC–HRMS analysis.

## Figures and Tables

**Table 1:** Exact masses (*m*/*z*) of prominent diagnostic product ions from [M−H]^−^ (ions A–D) and [M + Na]^+^ (ions E–L) observed during LC–MS^n^ (method A) and LC–HRMS/MS (method B) analysis of samples containing OA analogues **1**–**9** (see Figure 1 for R-groups and exact masses of precursor ions) *^a^*.

^ 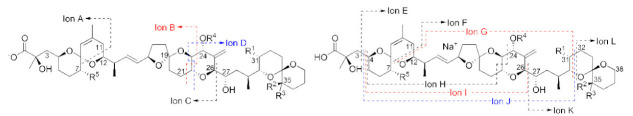 ^													
	***t*_R_*^b^***	**Ion A**	** Ion B**	**Ion C**	**Ion D**	**Ion E**	**Ion F**	** Ion G**	**Ion H**	** Ion I**	**Ion J**	**Ion K**	**Ion L**
**1**	13.42	255.1238	*463.2337*	563.2862	321.2071	723.4079	571.3241	443.2404	*383.1829*	483.2353	595.3241	263.1618	151.0730
**2**	14.15	255.1238	*463.2337*	563.2862	321.2071	723.4079	571.3241	429.2248	*383.1829*	483.2353	581.3085	263.1618	165.0886
**3**	15.75	255.1238	*463.2337*	563.2862	335.2228	737.4235	585.3398	443.2404	*383.1829*	483.2353	595.3241	277.1774	165.0886
**4**	9.12	255.1238	463.2337	725.3390	321.2071	885.4607	733.3770	605.2932	383.1829	*645.2881*	757.3770	263.1618	151.0730
**5**	9.48	255.1238	463.2337	725.3390	321.2071	885.4607	733.3770	591.2776	383.1829	*645.2881*	743.3613	263.1618	165.0886
**6**	11.01	255.1238	463.2337	725.3390	335.2228	899.4763	747.3926	605.2932	383.1829	*645.2881*	757.3770	277.1774	165.0886
**7**	17.68	239.1289	*447.2388*	547.2913	321.2071	707.4130	571.3241	443.2404	*367.1880*	467.2404	579.3292	263.1618	151.0730
**8**	18.25	239.1289	*447.2388*	547.2913	321.2071	707.4130	571.3241	429.2248	*367.1880*	467.2404	565.3136	263.1618	165.0886
**9**	19.76	239.1289	*447.2388*	547.2913	335.2228	721.4286	585.3398	443.2404	*367.1880*	467.2404	579.3292	277.1774	165.0886

*^a^* LC–MS^n^ data were at unit-mass resolution only. All precursor and product ions were detected (and almost always with ±5 ppm) in samples containing **1**–**9** at sufficient concentration for adequate signal-to-noise, with the exception of those in italic text (which when seen were at ~0.5–1% of base-peak intensity). Example spectra are shown in Figures 4, 5, 7, 8 and Figures S3, S4 and S6. *^b^* Retention times (min) with LC–HRMS method B (see Figure 3).

**Table 2:** ^1^H and ^13^C NMR assignments for **1** and **2** (from Larsen et al. [19]) and their 24-*O*-β-d-glucopyranosides (**4** and **5**) in CD_3_OD at 600 MHz. NMR assignments for the glucosyl moieties are presented in Table 3.

	OA (1)		24-GlcOA (4)		24-GlcDTX2 (5)		DTX2 (2)	
Atom	^13^C	^1^H	^13^C	^1^HG	^13^C	^1^H	^13^C	^1^H
1	182.8		182.1		183.3		180.6	
2	76.3		76.5		76.2		76.1	
3	46.7	1.65, 1.91	46.8	1.69, 1.88	46.7	1.68, 1.89	45.7	1.65, 2.01
4	68.9	4.08	68.7	4.09	68.7	4.09	69.3	4.08
5	33.4	1.30, 1.89	33.6	1.30, 1.79	33.6	1.31, 1.81	33.4	1.34, 1.79
6	28.3	1.66, 1.94	28.4	1.65, 1.96	28.3	1.65, 1.97	28.0	1.65, 1.98
7	73.4	3.35	73.4	3.36	73.4	3.36	73.1	3.37
8	97.5		97.5		97.6		97.6	
9	123.8	5.26	123.9	5.28	123.8	5.28	123.2	5.29
10	139.3		139.2		139.2		139.9	
11	34.0	1.82, 1.98	34.1	1.83, 1.97	34.1	1.84, 1.97	34.1	1.88, 1.96
12	71.8	3.87	71.8	3.91	71.8	3.91	71.9	3.75
13	43.2	2.35	43.5	2.36	43.4	2.36	43.3	2.34
14	137.2	5.95	137.3	5.95	137.2	5.93	137.4	5.83
15	132.0	5.50	131.8	5.50	131.8	5.49	132.3	5.52
16	80.6	4.66	80.7	4.60	80.7	4.60	80.5	4.62
17	31.5	1.60, 2.21	31.6	1.61, 2.18	31.6	1.61, 2.18	31.5	1.61, 2.20
18	38.1	1.85, 2.00	38.1	1.89, 2.00	38.1	1.89, 2.02	38.0	1.85, 2.00
19	107.1		107.4		107.4		107.1	
20	34.2	1.84–1.87	34.3	1.85–1.92	34.3	1.85–1.92	34.1	1.82–1.89
21	27.7	1.80, 1.90	28.0	1.80, 1.91	28.0	1.81, 1.90	27.7	1.79, 1.90
22	71.4	3.63	71.6	3.72	71.6	3.72	71.4	3.64
23	78.3	3.42	76.9	3.59	76.8	3.59	78.1	3.40
24	72.1	4.07	75.9	4.44	75.5	4.44	72.2	4.10
25	147.1		143.9		144.1		147.4	
26	86.4	3.94	86.8	3.93	87.0	3.94	86.4	3.97
27	66.3	4.08	65.9	4.14	66.1	4.13	67.0	4.07
28	36.8	0.96, 1.37	37.0	0.92, 1.41	38.9	1.37, 1.42	38.6	1.37, 1.44
29	32.3	1.89	32.5	1.87	35.3	1.82	35.3	1.83
30	76.8	3.27	76.8	3.27	75.0	3.42	75.0	3.42
31	28.8	1.81	28.9	1.82	28.8	1.27, 1.52	28.7	1.27, 1.52
32	27.5	1.38, 2.00	27.6	1.38, 2.00	20.1	1.61, 1.74	20.1	1.74, 1.60
33	31.2	1.36, 1.59	31.3	1.32, 1.59	33.4	1.17, 1.67	33.4	1.16, 1.68
34	97.0		97.0		99.2		99.3	
35	37.0	1.43, 1.61	37.1	1.41, 1.61	37.4	1.62	37.4	1.61
36	19.8	1.53, 1.88	19.9	1.53, 1.88	26.9	1.32, 2.17	26.7	1.29, 2.17
37	26.5	1.49–1.55	26.6	1.49–1.56	20.9	1.28, 1.78	21.0	1.78, 1.26
38	61.3	3.51, 3.71	61.4	3.52, 3.71	61.4	3.51, 3.70	61.4	3.51, 3.70
25 =CH_2_	112.6	5.04, 5.38	114.6	5.11, 5.67	114.3	5.10, 5.69	112.1	5.04, 5.36
10-Me	23.1	1.72	23.2	1.72	23.2	1.73	23.1	1.75
13-Me	17.1	1.11	17.1	1.11	17.2	1.11	16.8	1.07
29-Me	16.6	1.05	17.0	1.07	14.5	0.96	14.4	0.95
2-Me	27.9	1.31	27.6	1.32	27.5	1.32	27.7	1.34
31-Me	11.1	0.93	11.2	0.94				
35-Me					14.6	1.00	14.7	0.99

**Table 3:** ^1^H and ^13^C NMR assignments for the glucosyl moieties of OA and DTX2 24-*O*-β-d-glucopyranosides (**4** and **5**) in CD_3_OD at 600 MHz.

	OA 24-*O*-β-d-Glucoside (4)			DTX2 24-*O*-β-d-Glucoside (5)		
atom	^13^C	^1^H	Mult, *J* (Hz)	^13^C	^1^H	Mult, *J* (Hz)
1′	102.5	4.39	d, 7.8 *^a^*	102.5	4.39	d, 7.8 *^a^*
2′	75.0	3.39	dd, 7.8, 9.0 *^b^*	75.0	3.39	dd, 7.8, 9.0 *^b^*
3′	78.1	3.35	~t, *J* = 9.0 *^b^*	78.2	3.32	~t, 9.0 *^b^*
4′	71.9	3.28	~t, 9.0 *^b^*	71.9	3.29	~t, 9.0 *^b^*
5′	78.5	3.20	m	78.4	3.19	m
6′	63.1	3.64	dd, 12.0, 5.5 *^b^*	63.1	3.64	dd, 12.0, 5.5 *^b^*
		3.83	dd, 12.0, 2.2 *^b^*		3.83	dd, 12.0, 2.2 *^b^*

*^a^* ±0.1 Hz as determined in ^1^H NMR spectra. *^b^* ±0.3 Hz as determined in 1D-SELTOCSY NMR spectra.

**Table 4:** Percentage composition (*w*/*w*) and total concentrations of **1**–**9** in unhydrolyzed extracts analysed using LC–HRMS SIM (method B) *^a^*.

			Percentage of Sum of 1–9									Sum 1–9	
Sample	Description	Origin *^b^*	1	2	3	4	5	6	7	8	9	ng/mL	µg/g
Mixed standard	Std of **4**, **5** and **7**	N/A	0.4	0.2	0.07	21	72	ND	6.1	ND	ND	2100	
RM-Multi-toxin	Multitoxin RM	N/A	30	31	40	ND	ND	ND	ND	ND	ND	750	
D. acuta	VGO1065/1	ES	71	28	0.2	0.3	0.05	ND	0.09	0.04	ND	38,000	
*D. caudata* (conc.)	VGO1396/2	ES	77	12	10	0.5	ND	ND	ND	ND	ND	5.2	
*D. caudata* (conc.)	VGO1396/4	ES	68	14	17	0.3	ND	ND	ND	ND	ND	2.0	
D. acuminata	VGO1349/3	ES	99	ND	0.01	0.6	0.01	ND	0.1	ND	ND	2000	
*D. acuminata*	VGO1349/5	ES	100	ND	0.03	0.07	ND	ND	0.1	ND	ND	1200	
*D. acuminata*	VGO1391/7	ES	100	ND	0.01	0.3	ND	ND	0.1	ND	ND	2700	
P. lima	CCMP2579	RI, US	7.3	ND	93	ND	ND	ND	0.01	ND	0.04	2300	
*P. lima*	JHPL2	NS, CA	7.4	ND	92	ND	ND	ND	0.01	ND	0.07	18,000	
*P. lima*	JHPL3	NS, CA	5.8	ND	94	ND	ND	ND	0.01	ND	0.07	12,000	
*P. lima*	KP200	PE, CA	27	ND	73	ND	ND	ND	0.02	ND	0.03	13,000	
*P. lima*	KP209	NB, CA	37	ND	60	ND	ND	ND	2.1	ND	0.03	4100	
*P. lima*	IP197	NS, CA	44	ND	45	ND	ND	ND	11	ND	0.05	3100	
*P. lima*	CCMI1036	IE	5.0	ND	95	ND	ND	ND	ND	ND	0.08	1900	
*P. lima*	PL20V, Galicia	ES	81	ND	17	ND	ND	ND	0.08	ND	0.02	1800	
FDMT1-SPE (conc.)	CRM concentrate	IE/NO	19	55	13	8.6	3.9	0.3	0.1	0.2	0.07	6500	
CRM FDMT1	CRM Extract	IE/NO	25	59	8.8	5.1	2.5	0.2	ND	ND	ND	120	
CRM DSP-Mus	CRM Extract	IE/NO	34	30	32	1.2	0.9	0.03	1.3	0.03	0.02	330	1.63
Mussel HP	Flødevigen	NO	14	20	57	1.5	0.4	6.9	ND	ND	ND	7.3	0.04
Whole mussel	Flødevigen	NO	7.4	8.5	78	0.4	ND	5.4	ND	ND	ND	61	0.32
Whole broth	Indian Pt.	NS, CA	0.2	ND	98	ND	ND	1.4	ND	ND	0.02	93	0.52
Whole mussel	Bonavista Bay	NF, CA	0.2	ND	97	ND	ND	2.5	ND	ND	0.5	41	0.33
Whole mussel	Ship Harbour	NS, CA	92	ND	4.2	3.9	ND	0.2	ND	ND	ND	50	0.48
Whole mussel *^c^*	Castlemaine Harbour	IE	35	5.7	3.8	54	1.3	0.2	ND	ND	ND	10	0.09
Whole mussel *^d^*	Bantry Middle	IE	67	8.7	2.0	21	0.5	0.1	0.7	ND	ND	36	0.43
SPATT (conc.)	Ship Harbour	NS, CA	8.8	ND	91	ND	ND	0.2	ND	ND	0.1	37	

*^a^* All values are rounded to two significant figures, except for values less than 0.1 (rounded to one significant figure). Consequently, the sum of the percentages of **1**–**9** for samples may not add to exactly 100%; ND, not detected. *^b^* In the format standard two-letter province/state code (where relevant), two-letter country code; N/A, not applicable. *^c^* Extract from entry 12 in Table 5. *^d^* Extract from entry 15 in Table 5.

**Table 5:** LC–HRMS analysis (method D) of shellfish samples submitted to the Irish monitoring program showing official reported results (hydrolyzed and with TEFs applied) for OA group toxins **1** and **2** (µg/g), and the abundance of their glucosides (**4** and **5**) expressed as a percentage (*w/w*) of **1** and **2** assuming identical response factors *^a^*.

					Reported Results		
	Location	Date *^b^*	Species	4 + 5 (%)	1	2	1 Equiv. *^c^*
1	Ardgroom	02/06/20	*M. edulis*	5.5	2.68	<LOD	2.68
2	Kilmakilloge	02/06/20	*M. edulis*	6.7	2.61	<LOD	2.61
3	Mine Head Ground	03/06/20	*P. maximus*	ND	0.11	0.03	0.13
4	Youghal Bay	10/06/20	*S. solida*	ND	0.13	<LOD	0.13
5	Valentia River	11/06/20	*C. gigas*	ND	0.12	<LOD	0.12
6	Castlemaine Harbour	24/06/20	*C. edule*	ND	0.20	<LOD	0.20
7	Tahilla	30/06/20	*M. edulis*	20.2	0.69	0.02	0.70
8	Bantry Middle	30/06/20	*M. edulis*	16.8	1.21	0.08	1.26
9	Gouleenacoush	01/07/20	*M. edulis*	17.2	1.45	<LOD	1.45
10	Adrigole	01/07/20	*M. edulis*	14.1	0.66	0.07	0.70
11	Tahilla	13/07/20	*M. edulis*	39.2	0.33	0.04	0.35
12	Castlemaine Harbour *^d^*	13/07/20	*M. edulis*	58.0	0.09	<LOQ	0.09
13	Castlemaine Harbour	13/07/20	*M. edulis*	44.1	0.08	<LOQ	0.08
14	Bantry North	13/07/20	*M. edulis*	40.5	0.22	0.06	0.26
15	Bantry Middle	13/07/20	*M. edulis*	21.4	0.40	0.05	0.43
16	South of Smalls Ground	04/08/20	*P. maximus*	ND	0.10	0.03	0.12
17	Bantry North *^d^*	31/08/20	*M. edulis*	5.7	0.35	0.73	0.79
18	Bantry South	31/08/20	*M. edulis*	4.9	0.84	0.76	1.30
19	Ardgroom	31/08/20	*M. edulis*	26.0	0.07	0.12	0.14

*^a^* Assuming a 1:1 relative molar response; ND = not detected; LOD, limit of detection; LOQ, limit of quantitation; no **3** or **6**–**9** were detected in any of the samples. *^b^* Date format dd/mm/yy. *^c^* OA-equivalents calculated from official TEFs for **1** and **2**. *^d^* See Figure S43.

**Table 6:** LC–MS/MS (method E) analysis of shellfish samples submitted to the New Zealand non-commercial monitoring programme, showing concentrations of OA-group toxins (µg/g) obtained using negative ionization mode *^a^*.

			Non-Hydrolyzed			Hydrolyzed		
Location	Species	Date *^b^*	1	3	4	1	3	4
Sumner	*M. edulis*	04/01/17	0.151	0.008	0.011	0.226	<LOQ	0.013
Akaroa harbour	*M. edulis*	05/07/17	0.420	0.010	0.010	0.826	0.012	<LOQ
Akaroa harbour	*M. edulis*	12/07/17	0.268	0.005	0.004	0.590	<LOQ	<LOQ
Akaroa harbour	*M. edulis*	31/01/17	0.412	0.018	0.006	0.833	0.022	<LOQ
Akaroa harbour	*M. edulis*	07/08/17	0.514	0.025	0.0010	0.978	0.032	<LOQ
Motukiekie Beach	*P. canaliculus*	25/02/19	0.025	ND	ND	0.201	ND	ND
Cape Foulwind	*P. canaliculus*	04/03/19	0.021	ND	ND	0.191	ND	ND
Cape Foulwind	*P. canaliculus*	18/03/19	0.020	ND	ND	0.220	ND	ND
Motukiekie Beach	*M. edulis*	01/04/19	0.037	ND	ND	0.220	ND	ND
Sumner	*P. subtriangulata*	02/06/20	ND	<LOQ	ND	ND	0.025	ND
Sumner	*P. canaliculus*	09/06/20	<LOQ	0.009	ND	0.012	0.072	ND

*^a^* ND = not detected; LOQ, limit of quantitation (0.002 µg/g for non-hydrolyzed samples, 0.01 µg/g for hydrolyzed samples); compounds **2**, **5**, **8**, and **9** were not detected, while traces of **6** and **7** were detected, but only in positive ionization mode. *^b^*Date format dd/mm/yy.

## Supplementary Materials

The following can be found at https://www.mdpi.com/article/10.3390/toxins13080510/s1. Figure S1: Periodate cleavage of OA/DTX2-Glc by LC–HRMS; Figure S2: 1D-SELROESY NMR spectrum for 25 =CH_2_ (*E*) of DTX2-Glc (**5**); Figure S3: LC–HRMS/MS spectra of [M–H]^˗^ of OA/DTXs **1**–**3**; Figure S4: LC–HRMS/MS spectra showing modes of neutral loss of Glc from **4**–**6**; Figure S5: Proposed fragmentation for [M˗H]^-^ of **1** and **4**; Figure S6: LC–HRMS/MS spectra of [M + Na]^+^ of OA/DTXs **1**–**3**; Figure S7: LC–MS^2^ spectra of [M–H]^-^, [M + NH_4_]^+^, and [M + Na]^+^ of OA-Glc (**4**); Figure S8: Proposed fragmentation for [M + Na]^+^ of **4** and **5**; Figure S9: LC–MS^2^ spectra of [M + NH_4_]^+^, of putative OA-diglucoside; Figure S10: ^1^H NMR spectrum of OA-Glc (**4**) in CD_3_OD; Figure S11: COSY NMR spectrum of OA-Glc (**4**) in CD_3_OD; Figure S12: TOCSY NMR spectrum of OA-Glc (**4**) in CD_3_OD; Figure S13: 1D-SELTOCSY NMR spectra of H-1′ of OA-Glc (**4**) in CD_3_OD; Figure S14: HSQC NMR spectrum of OA-Glc (**4**) in CD_3_OD; Figure S15: HMBC NMR spectrum of OA-Glc (**4**) in CD_3_OD; Figure S16: ROESY NMR spectrum of OA-Glc (**4**) in CD_3_OD; Figure S17: 1D-SELROESY NMR spectrum for H-1′ of OA-Glc (**4**) in CD_3_OD; Figure S18: 1D-SELROESY NMR spectrum for H-24 of OA-Glc (**4**) in CD_3_OD; Figure S19: 1D-SELROESY NMR spectrum for 25 =CH_2_ (*Z*) of OA-Glc (**4**) in CD_3_OD; Figure S20: 1D-SELROESY NMR spectrum for 25 =CH_2_ (*E*) of OA-Glc (**4**) in CD_3_OD; Figure S21: ^1^H NMR spectrum of DTX2-Glc (**5**) in CD_3_OD; Figure S22: Secondary methyl region of the ^1^H NMR spectrum of DTX2-Glc (**5**); Figure S23: COSY NMR spectrum of DTX2-Glc (**5**) in CD_3_OD; Figure S24: TOCSY NMR spectrum of DTX2-Glc (**5**) in CD_3_OD; Figure S25: 1D-SELTOCY NMR spectra for H-1′ of DTX2-Glc (**5**); Figure S26: HSQC NMR spectrum of DTX2-Glc (**5**) in CD_3_OD; Figure S27: HMBC NMR spectrum of DTX2-Glc (**5**) in CD_3_OD; Figure S28: ROESY NMR spectrum of DTX2-Glc (**5**) in CD_3_OD; Figure S29: 1D-SELROESY NMR spectrum for H-1′ of DTX2-Glc (**5**) in CD_3_OD; Figure S30: 1D-SELROESY NMR spectrum for H-24 of DTX2-Glc (**5**) in CD_3_OD; Figure S31: 1D-SELROESY NMR spectrum for 25 =CH_2_ (*Z*) of DTX2-Glc (**5**); Figure S32: Positive mode LC–HRMS chromatograms of [M + Na]^+^ adducts of **1**–**8**; Figure S33: Negative mode LC–MS chromatograms of *Dinophysis* culture extracts; Figure S34: Negative mode LC–HRMS chromatograms of a range of sample types; Figure S35: Negative mode LC–HRMS chromatograms of mussel tissue CRMs; Figure S36: Negative mode LC–HRMS chromatograms of FDMT1 spiking experiment; Figure S37: Negative mode HRMS/MS spectra of **4** in FDMT1 and the standard; Figure S38: Negative mode HRMS/MS spectra of **5** in FDMT1 and the standard; Figure S39: Negative mode HRMS/MS spectra of **7** in FDMT1 and the standard; Figure S40: Negative mode HRMS/MS spectra of **4**, **1**, and **7** at 70 eV; Figure S41: Negative mode LC–HRMS chromatograms of samples containing **1**–**6**; Figure S42: Negative LC–HRMS chromatograms of samples containing **1**–**3** and **7**–**9**; Figure S43: LC–HRMS analysis Irish *M. edulis* containing **1**, **2** and glucosides (**4**, **5**); Figure S44: LC–MS TIC chromatogram of CRM-FDMT1 with method E; Figure S45: LC–MS TIC chromatogram of New Zealand *M. edulis* with method E; Figure S46: LC–MS TIC chromatogram of New Zealand *M. edulis* with method E; Table S1: Ions detected in negative mode LC–HRMS/MS of **1**–**9**; Table S2: Ions detected in positive mode LC–HRMS/MS of Na^+^ adduct ions of **1**–**9**; Table S3: LC–HRMS analysis SPATT disc extracts from Ireland; Table S4: LC–HRMS (method C) analysis of Canadian, Norwegian, and Irish mussels; Table S5: Details of *Dinophysis* cultures; Table S6: MRM transitions for LC–MS/MS method E.
