# Supplementary material for: Identification of 24-O-β-d-Glycosides and 7-Deoxy-Analogues of Okadaic Acid and Dinophysistoxin-1 and -2 in Extracts from Dinophysis Blooms, Dinophysis and Prorocentrum Cultures, and Shellfish in Europe, North America and Australasia
Source: Toxins (Basel). 2021 Jul 21;13(8):510. doi: 10.3390/toxins13080510 (PMC8402559; doi:10.3390/toxins13080510)
Supplement: Supplementary file 1 [file toxins-13-00510-s001.zip › toxins-1267994 SU conversion.pdf]

# Supplementary Materials: Identification of 24-O-β-D-Glycosides and 7-Deoxy-Analogues of Okadaic Acid and Dinophysistoxin-1 and -2 in Extracts from Dinophysis Blooms, Dinophysis and Prorocentrum Cultures, and Shellfish in Europe, North America, and Australasia

**Alistair L. Wilkins, Thomas Rundberget, Morten Sandvik, Frode Rise, Brent K. Knudsen, Jane Kilcoyne, Beatriz Reguera, Pilar Rial, Elliott J. Wright, Sabrina D. Giddings, Michael J. Boundy, Cheryl Rafuse, and Christopher O. Miles**

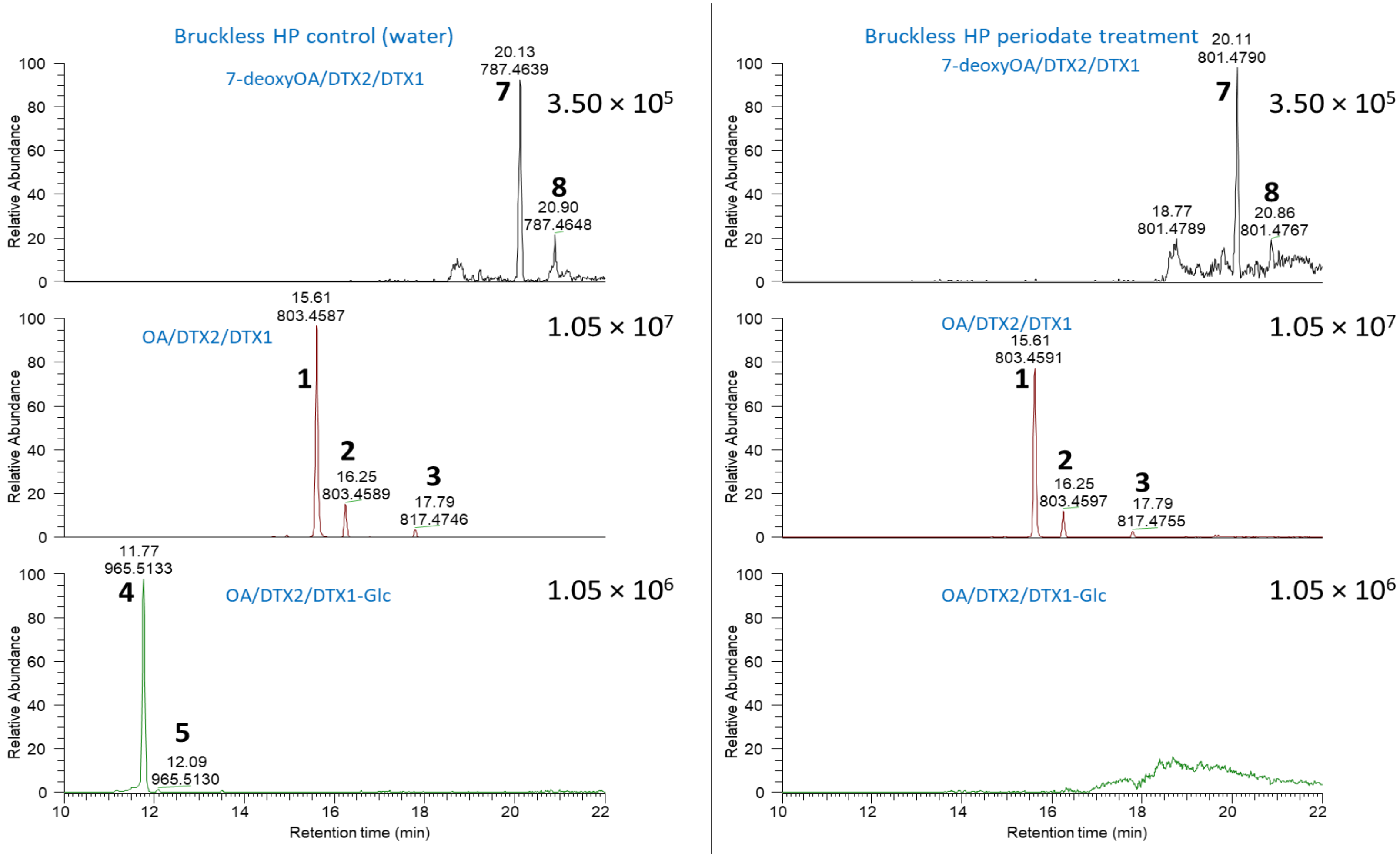


**Figure S1.** LC–HRMS extracted negative ion chromatograms of an extract of hepatopancreas from AZA-contaminated mussels from Bruckless before (left) and after treatment with sodium metaperiodate (right). The top panels are extracted for *m*/*z* 787.4638 and 801.4794 (**7**–**9**), the middle panels for *m*/*z* 803.4587 and 817.4744 (**1**–**3**), and the bottom panels for *m*/*z* 965.5115 and 979.5272 (**4**–**6**). Data is from analysis of existing historical full-scan LC–HRMS (method B) data from Kilcoyne et al. (2018).[1]

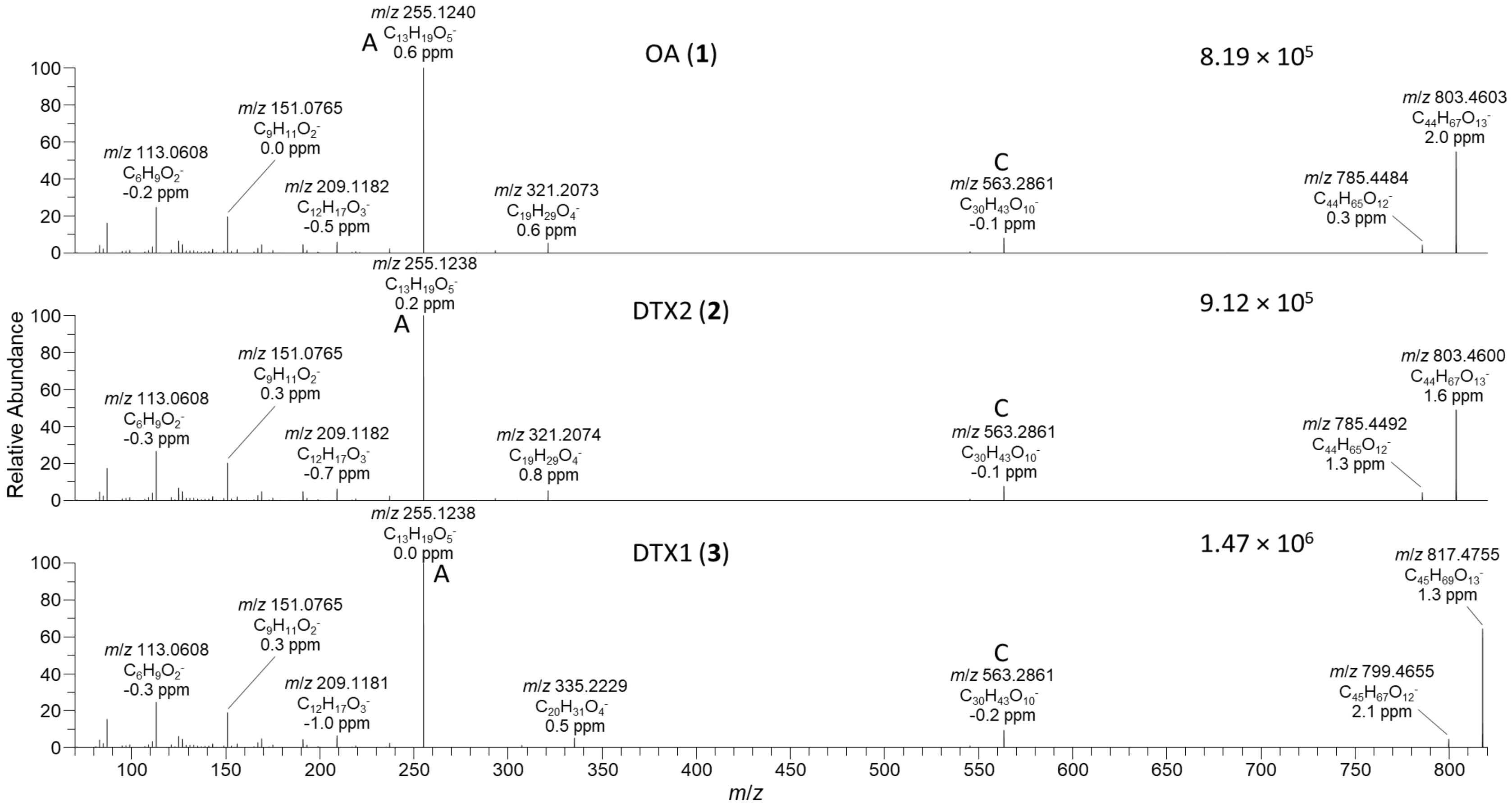


**Figure S2.** HRMS/MS spectra of [M−H]$^-$ for **1–3** obtained from NRC RM-Multi-toxin by LC–HRMS/MS (method B) in negative ionization mode. Spectra of **4–9** obtained under identical conditions are shown in Figures 4 and 7. Spectra of **1–3** in positive ionization mode are shown in Figure S6. Ions marked with letters refer to major structurally diagnostic ions listed in Table 1, with additional information in Figure S4 and Table S2.

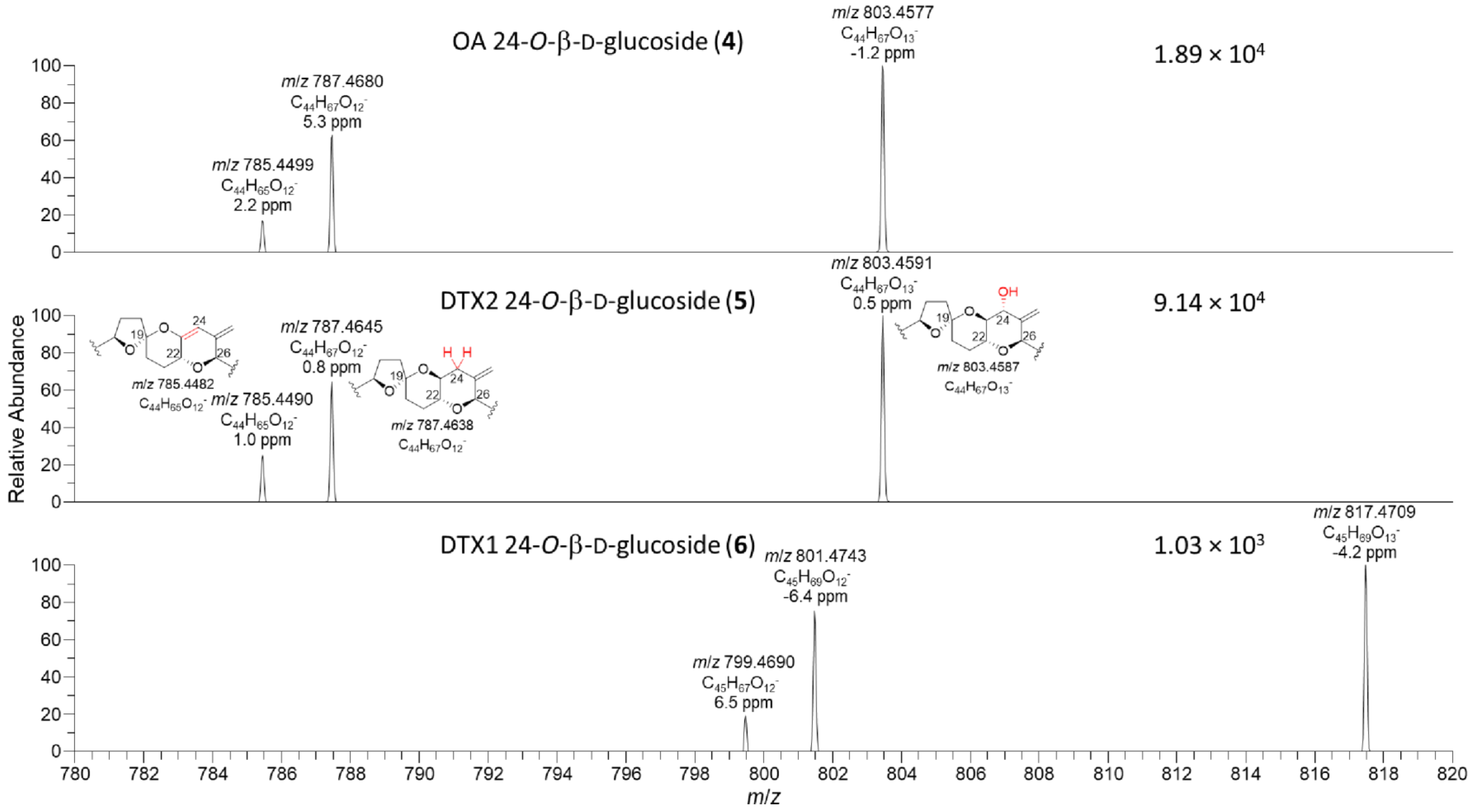


**Figure S3.** Expansion of the negative ion mode LC–HRMS/MS (method B) spectra of $[M{-}H]^-$ for 24-*O*-β-D-glucosides **4–6** showing the regions containing product ions resulting from the three modes for elimination of the glucosyl moiety (see Figure 4 for full spectra). The higher mass product ion arises from net elimination of dehydroglucose ($C_6H_{10}O_5$, 162.0528 Da), the lower mass ion from elimination of glucose ($C_6H_{12}O_6$, 180.0634 Da), and the product ion of intermediate mass from a less common elimination of gluconolactone ($C_6H_{10}O_6$, 178.0477 Da). Proposed structures for the resulting product ions are depicted on the MS/MS spectrum of **5**.

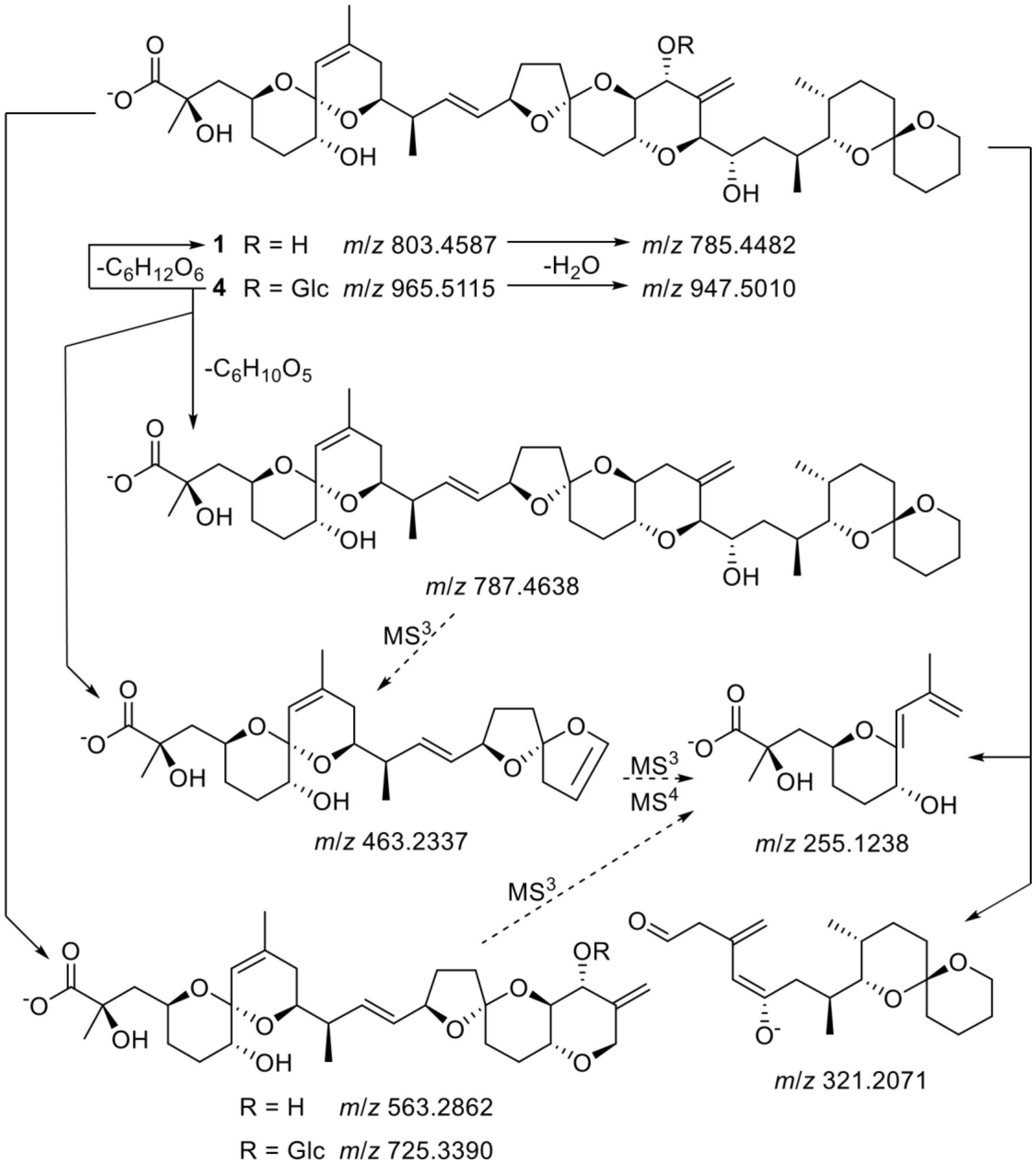


**Figure S4.** Proposed fragmentation pathways for OA (**1**) and its 24-*O*-β-D-glucoside (**4**) during LC–MS$^n$ (unit mass resolution) and LC–HRMS/MS in negative ion mode. The *m*/*z* values are exact masses, and are within ±5 ppm of the accurate masses observed during LC–HRMS/MS analysis. Solid arrows show fragmentations observed using [M–H]$^-$ as precursor ions with both LC–MS$^2$ and LC–HRMS/MS, while dashed arrows show fragmentations observed with LC–MS$^3$ and MS$^4$. Note that the product ions at *m*/*z* 787 and 463 were observed with glucoside-**4** but not with **1**. The corresponding product ions were also observed for **2**, **3**, **5**, **6** (LC–MS$^2$ and LC– HRMS/MS), and **7**–**9** (LC–HRMS/MS).

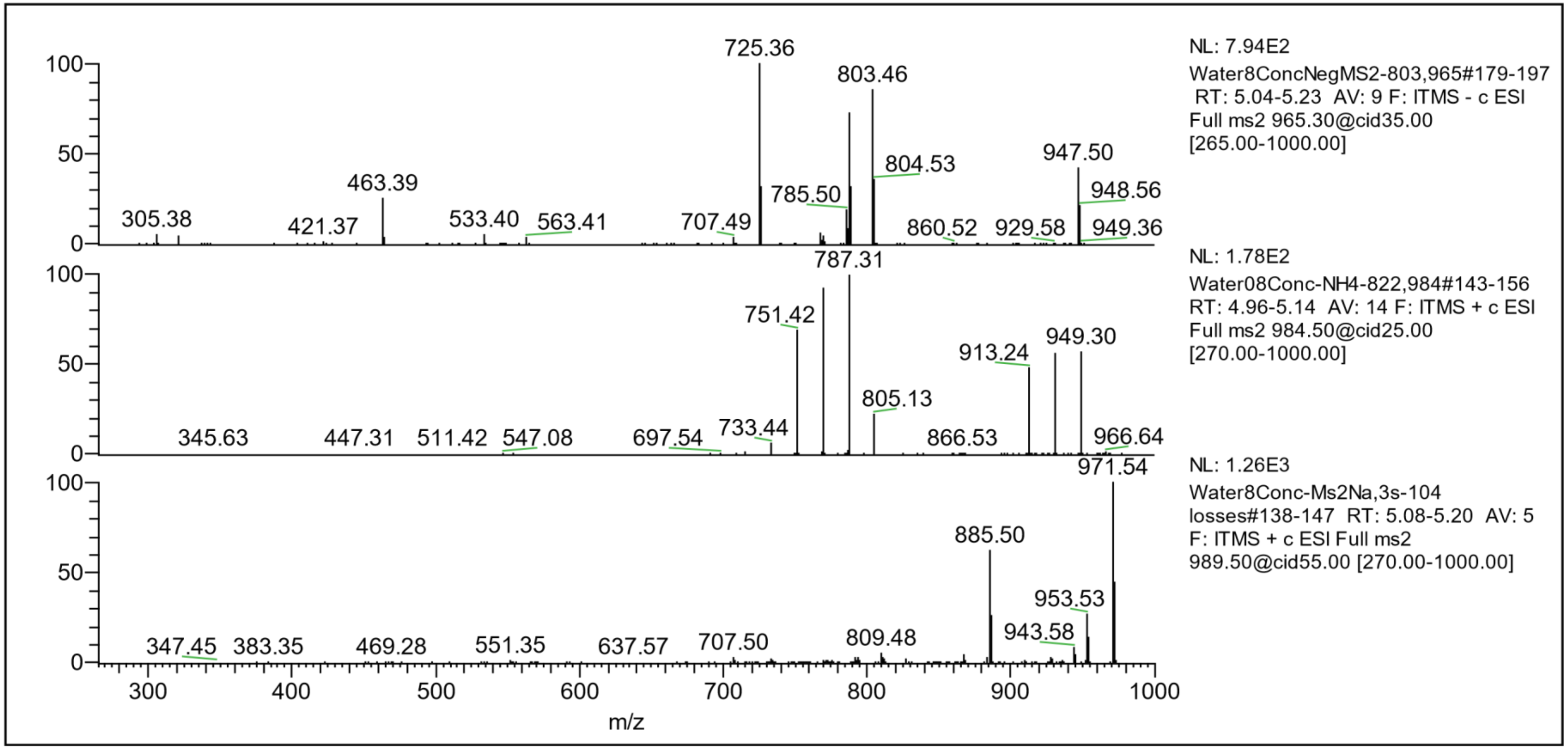


**Figure S5.** LC–MS² spectra of OA 24-*O*-β-D-glucoside (**4**), showing the [M−H]⁻ (upper), [M+NH₄]⁺ (middle), and [M+Na]⁺ (lower) spectra.

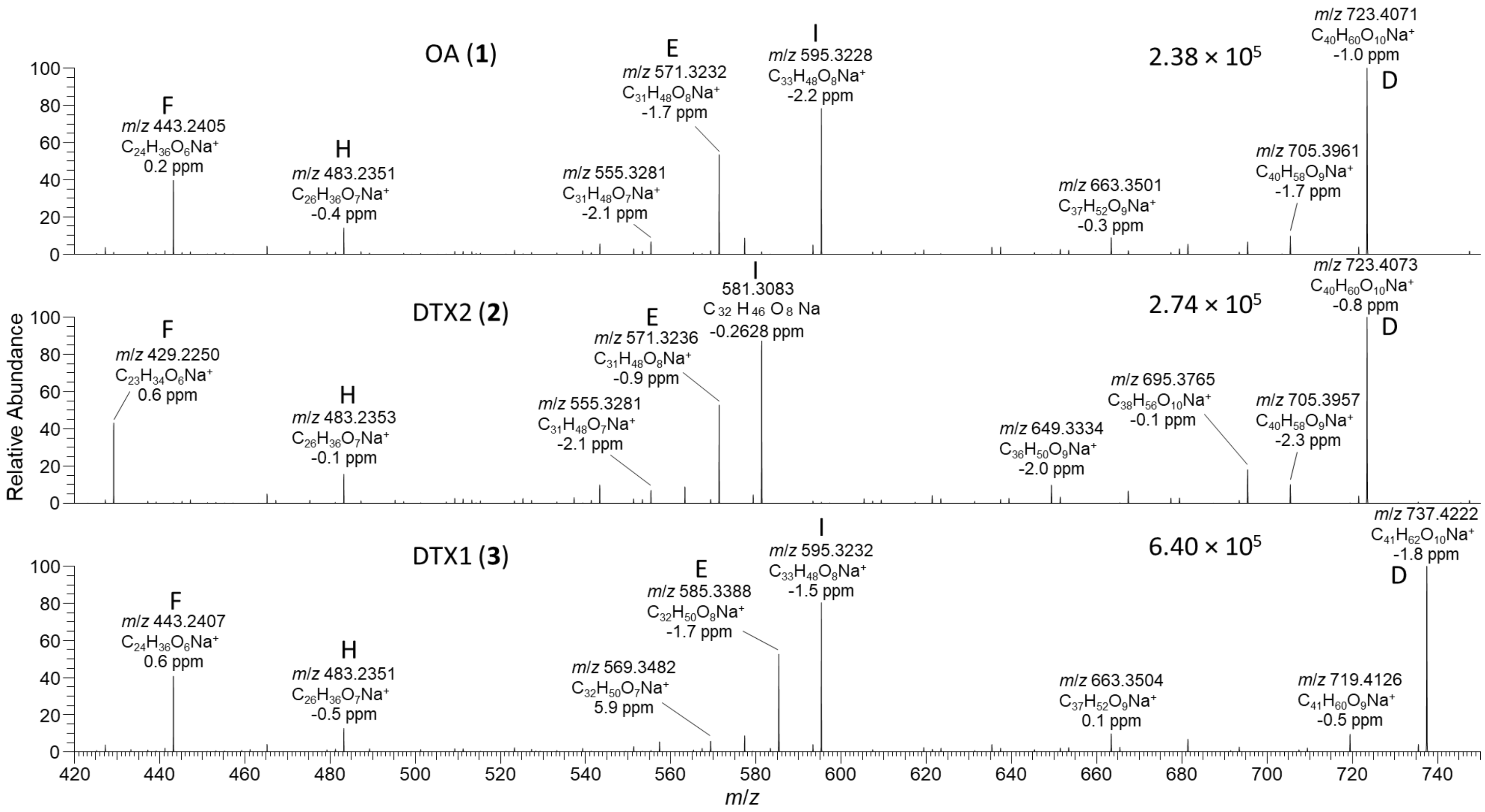


**Figure S6.** HRMS/MS spectra of $[M+Na]^+$ for **1**–**3** obtained by LC–HRMS/MS (method B) in positive ionization mode. Spectra of **1** and **2** were from the *D. acuta* extract, while the spectrum of **3** was from the extract of *P. lima* culture JHPL2. Spectra of **4**–**9** obtained under identical conditions are shown in Figures 5 and 8. Spectra of **1**–**3** in negative ionization mode are shown in Figure S2. Ions marked with letters refer to major structurally diagnostic ions listed in Table 1, with additional information Figure S7 and Table S3.

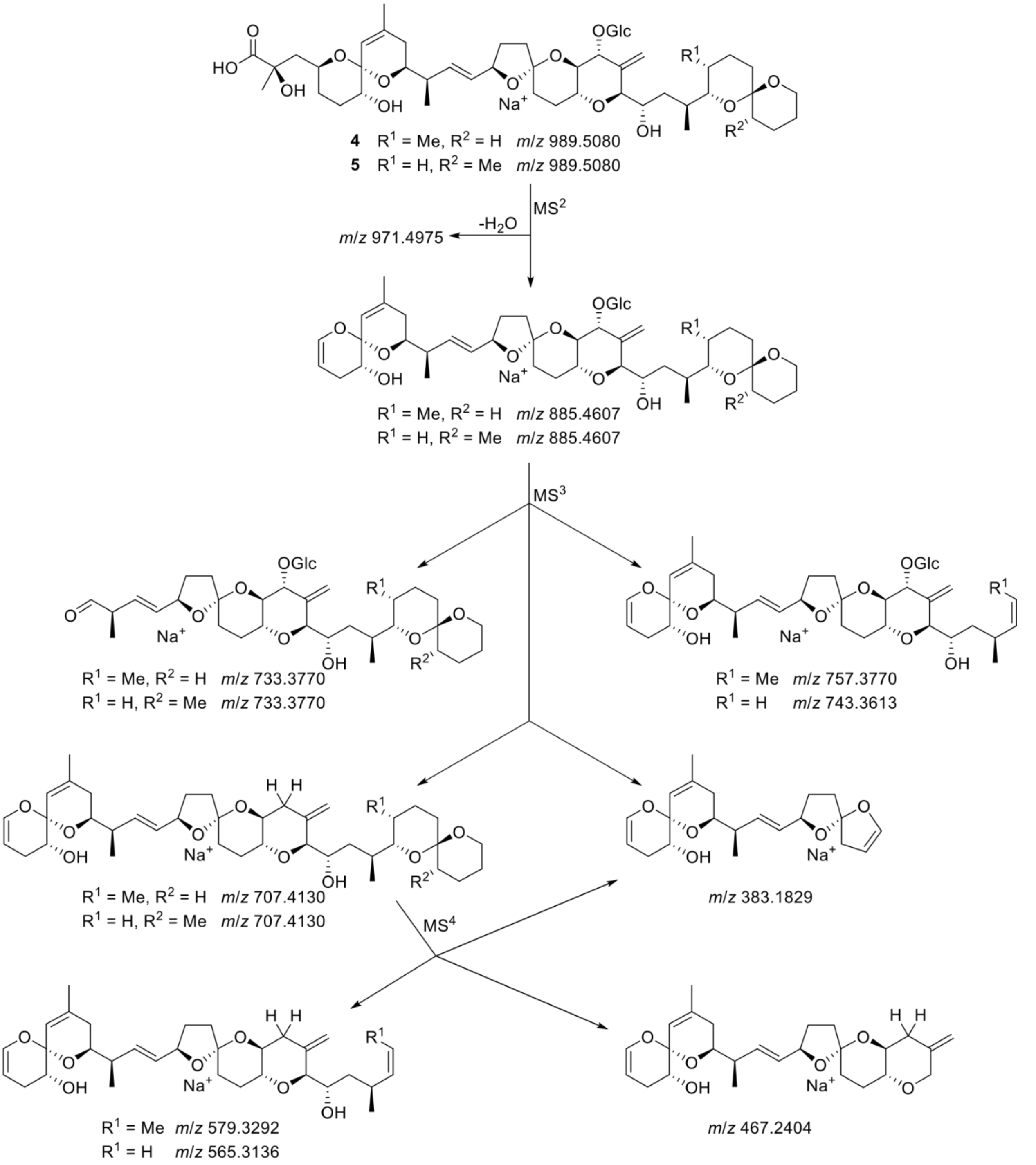


**Figure S7.** Proposed fragmentation of $[M+Na]^+$ for **4** and **5** based on LC–MS$^n$ (method A) and LC–HRMS/MS (method B). Product ions observed with the various stages of MS$^n$ (n = 2–4) are indicated, and were detected at unit mass resolution, but were also observed by HRMS/MS within ±5 ppm of the expected exact masses (which are indicated below the structures).

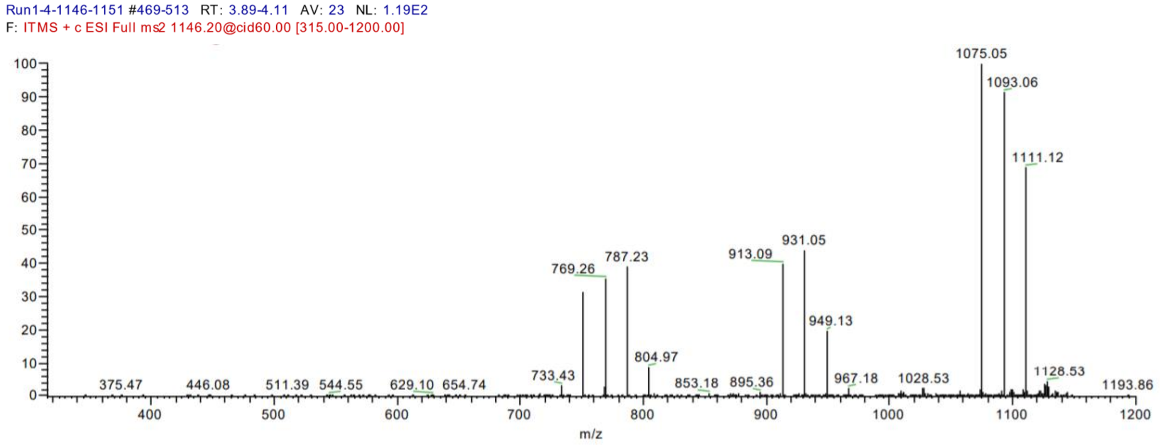


**Figure S8.** LC–MS² of the minor peak which exhibited a $[M+NH_4]^+$ at *m*/*z* 1146. Two series of [M−35 ($NH_4OH$)−18 $(H_2O)_n$−162 (dehydrated glucose or other glycosyl residues)]⁺ product ions are apparent, consistent with a diglycoside of OA.

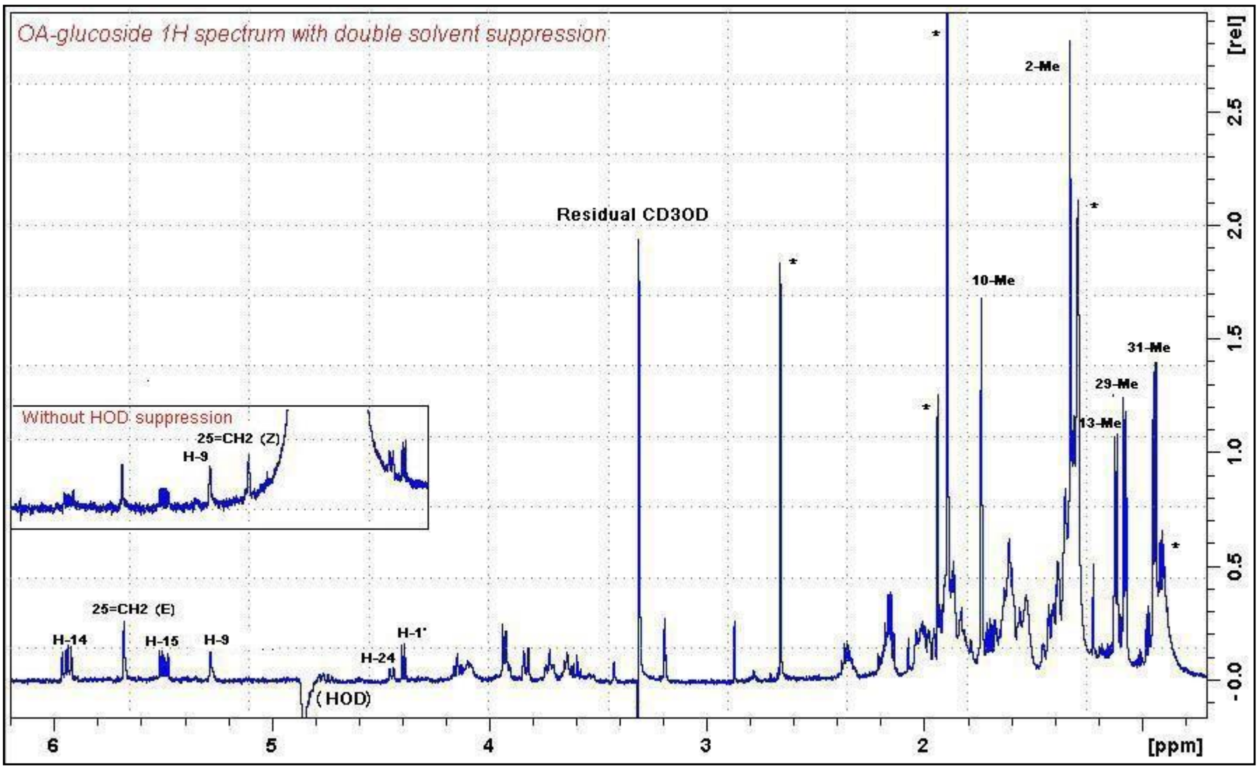


**Figure S9.** $^{1}$H NMR spectrum of OA-glc (**4**) in $CD_3OD$ at 600 MHz. Major impurity peaks are marked with asterisks. The inset shows the low-field region without solvent suppression.

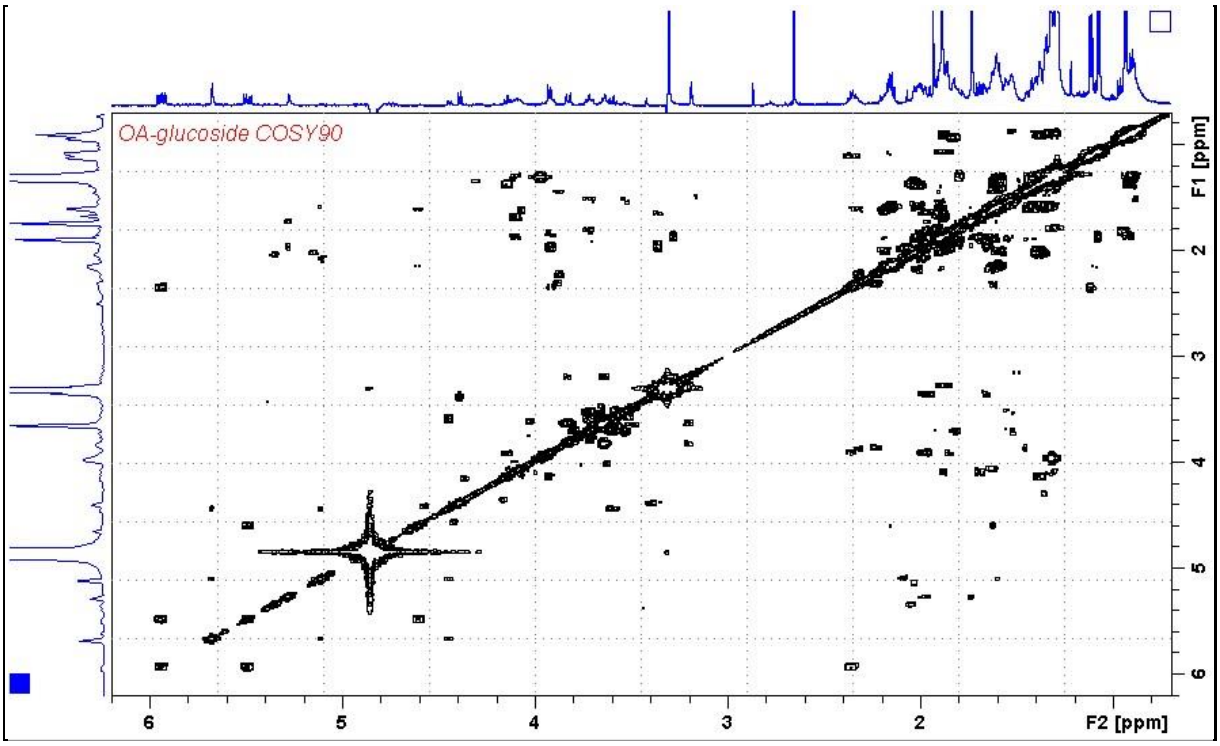


**Figure S10.** COSY NMR spectrum of OA-glc (**4**) in $CD_3OD$ at 600 MHz.

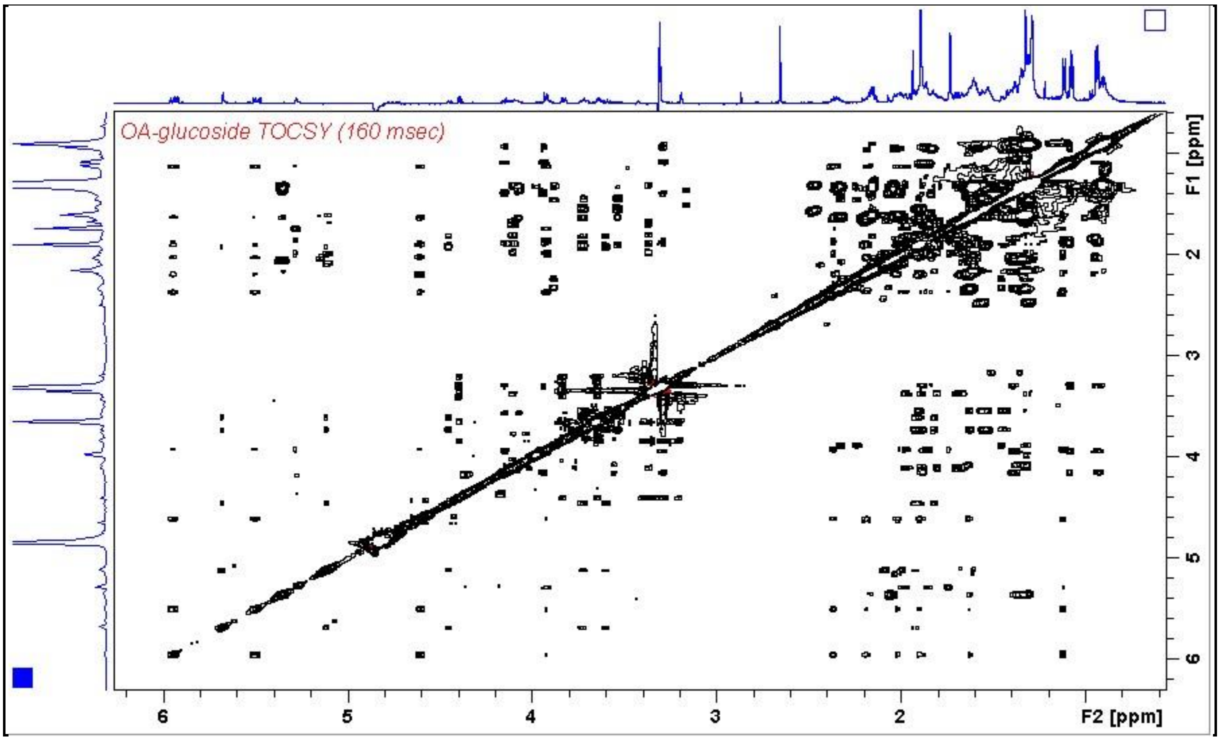


**Figure S11.** TOCSY NMR spectrum of OA-glc (**4**) in $CD_3OD$ at 600 MHz.

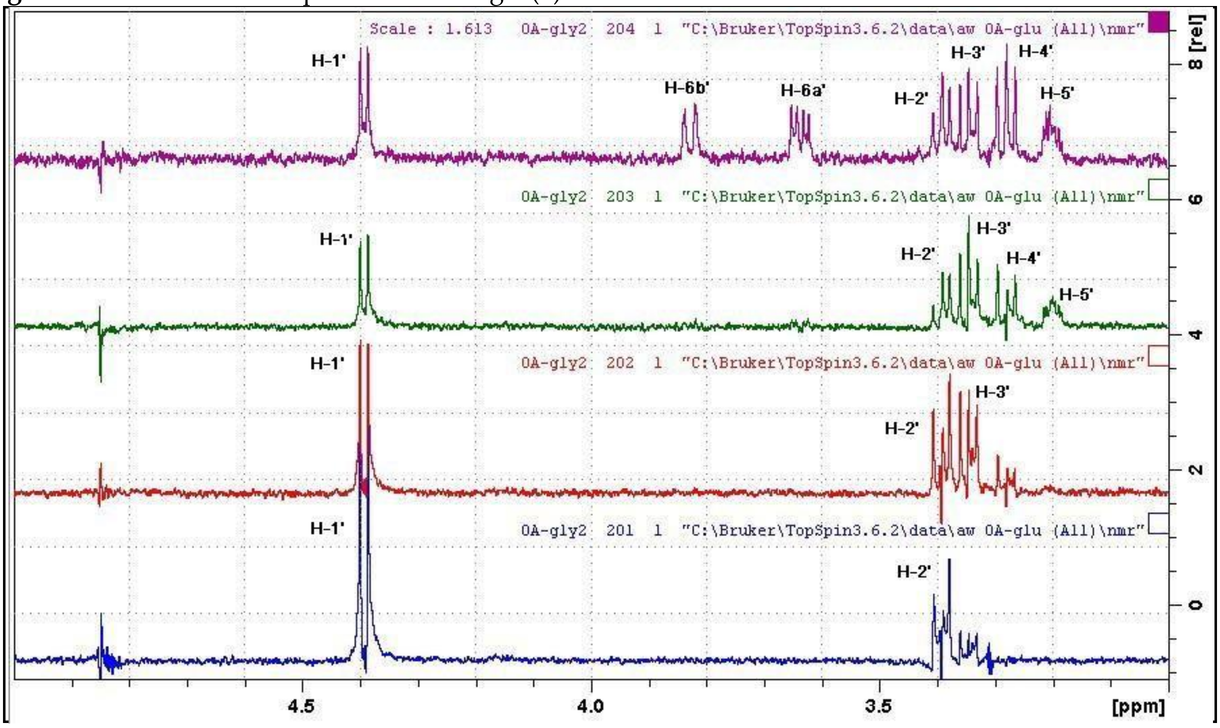


**Figure S12.** 1D-SELTOCY (d9 = 30, 50, 80 and 160 ms (from bottom to top)) NMR spectra at 600 MHz determined for H-1′ of OA 24-*O*-β-D-glucoside (**4**), which identify the H-2′ to H-6′ signals of the glucoside unit.

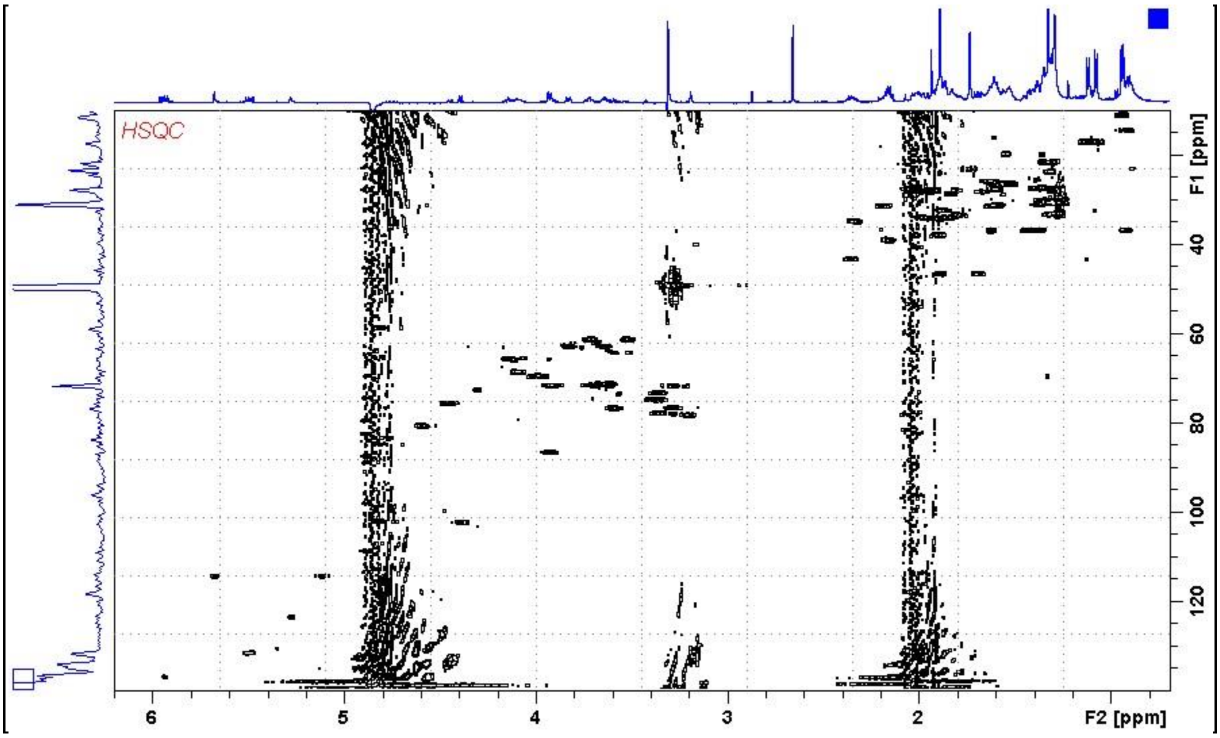


**Figure S13.** HSQC NMR spectrum of OA-Glc (**4**) in $CD_3OD$.

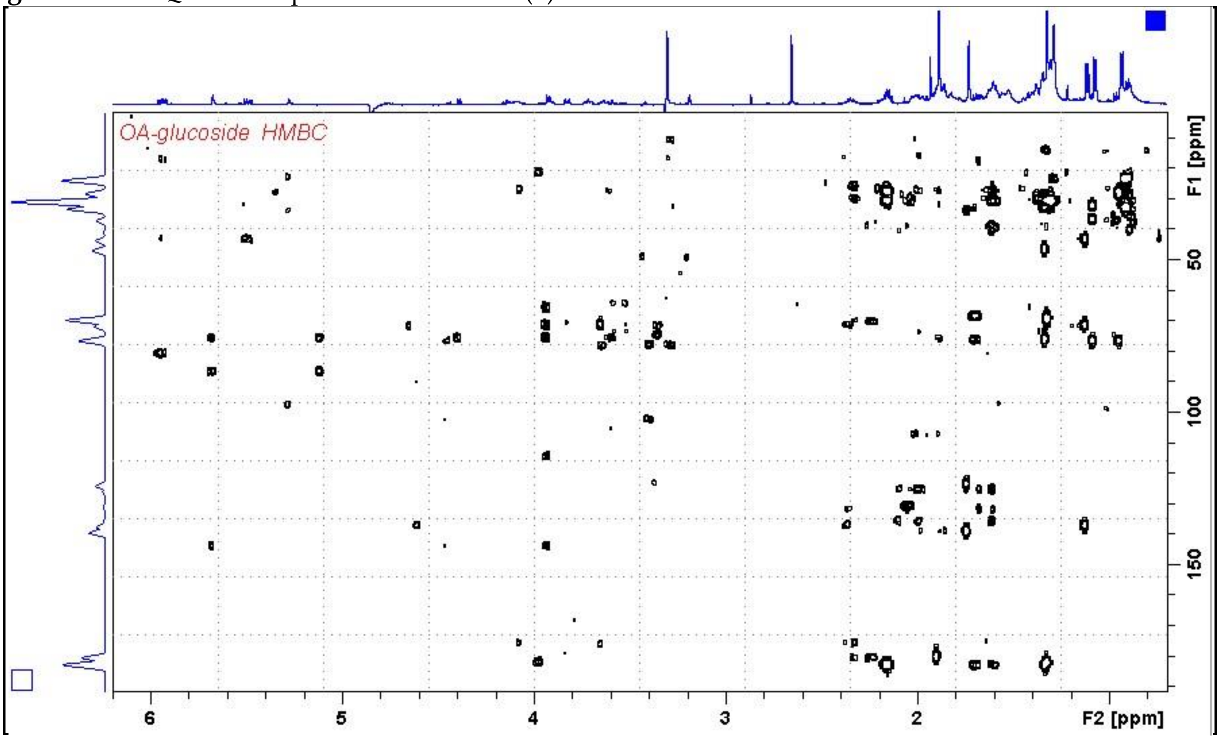


**Figure S14.** HMBC NMR spectrum of OA-Glc (**4**) in $CD_3OD$.

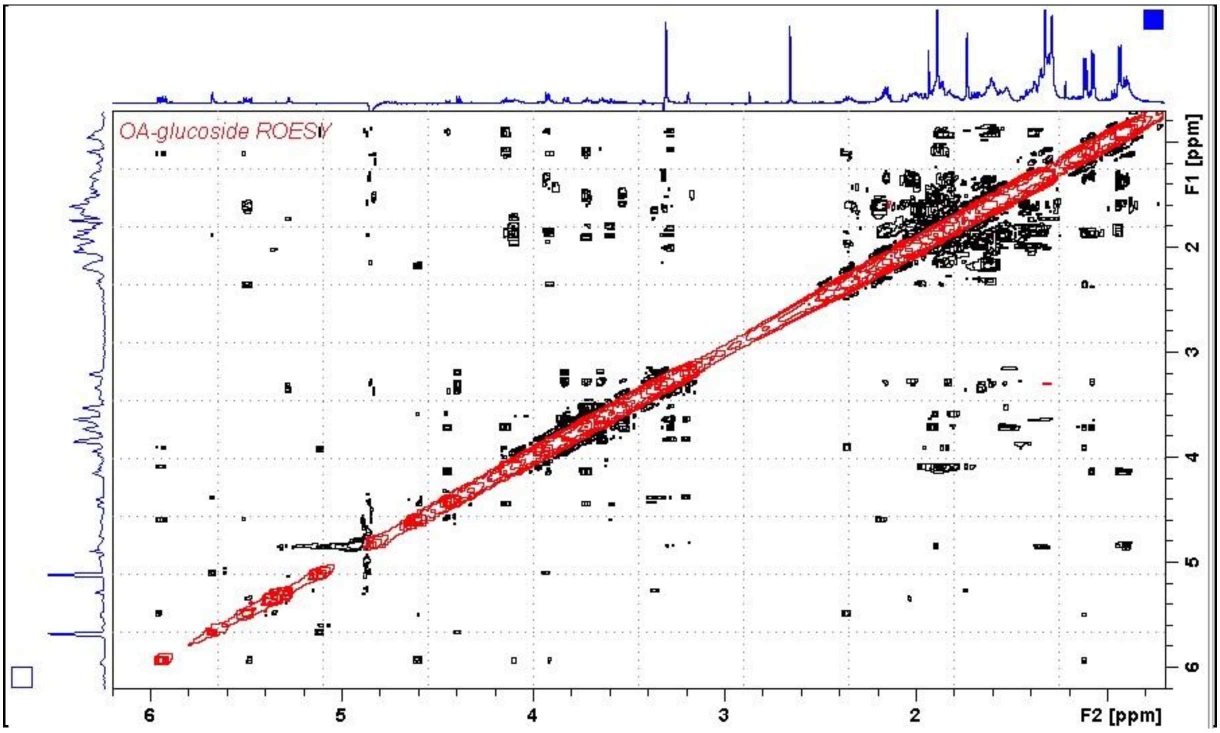


**Figure S15.** ROESY NMR spectrum of OA-Glc (**4**) in $CD_3OD$.

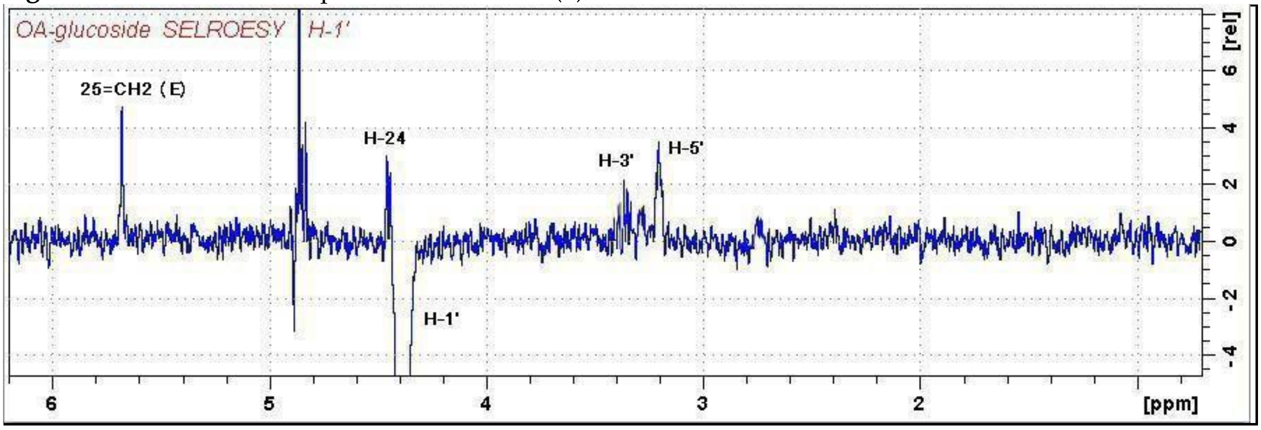


**Figure S16.** 1D-SELROESY NMR spectrum for H-1′ of OA-Glc (**4**) in $CD_3OD$.

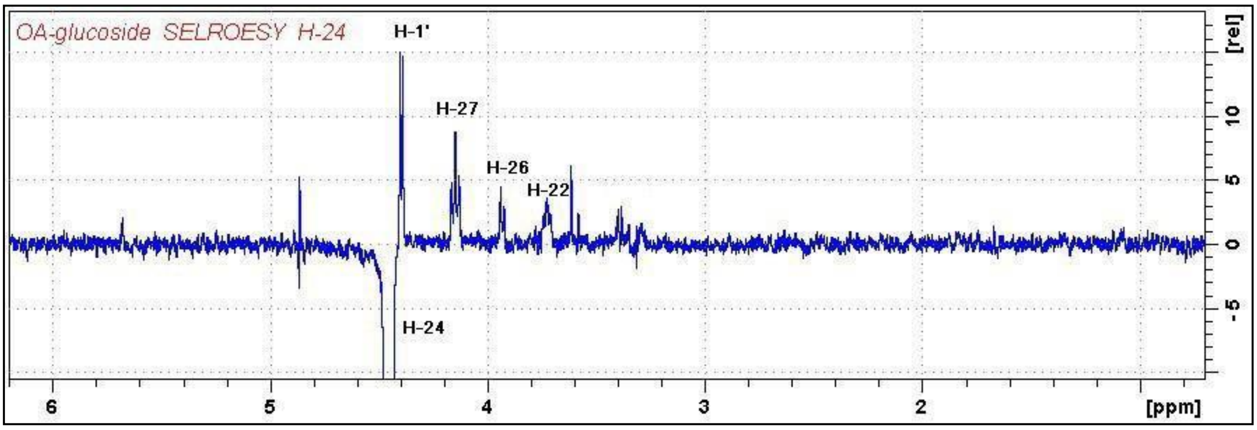


**Figure S17.** 1D-SELROESY NMR spectrum for H-24 of OA-Glc (**4**) in $CD_3OD$.

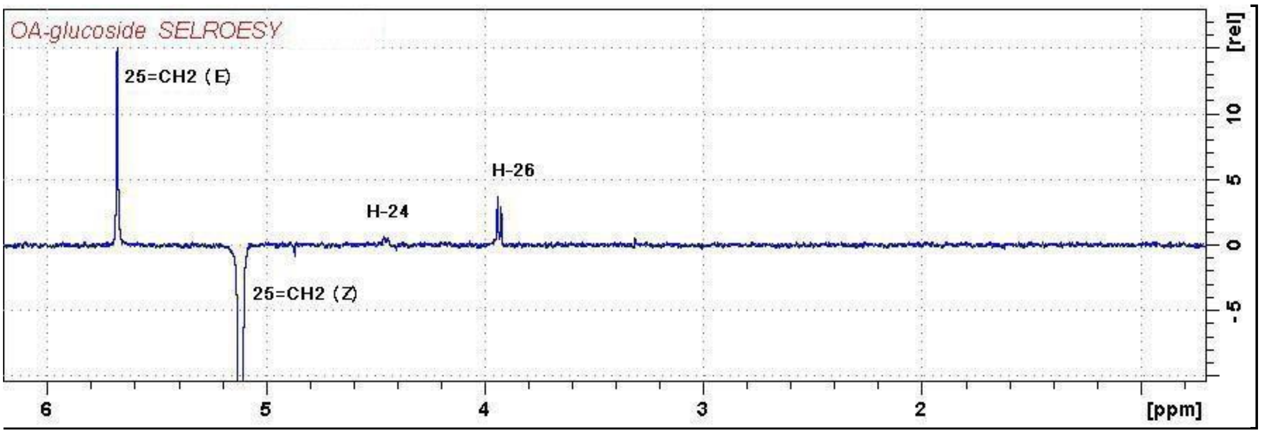


**Figure S18.** 1D-SELROESY NMR spectrum for 25=$CH_2$ (*Z*) of OA-Glc (**4**) in $CD_3OD$.

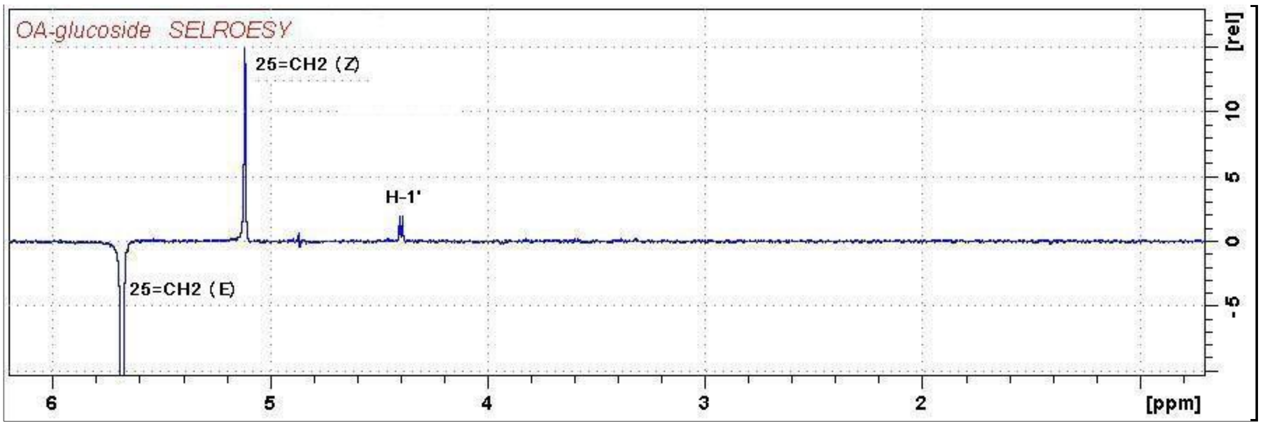


**Figure S19.** 1D-SELROESY NMR spectrum for 25=$CH_2$ (*E*) of OA-Glc (**4**) in $CD_3OD$.

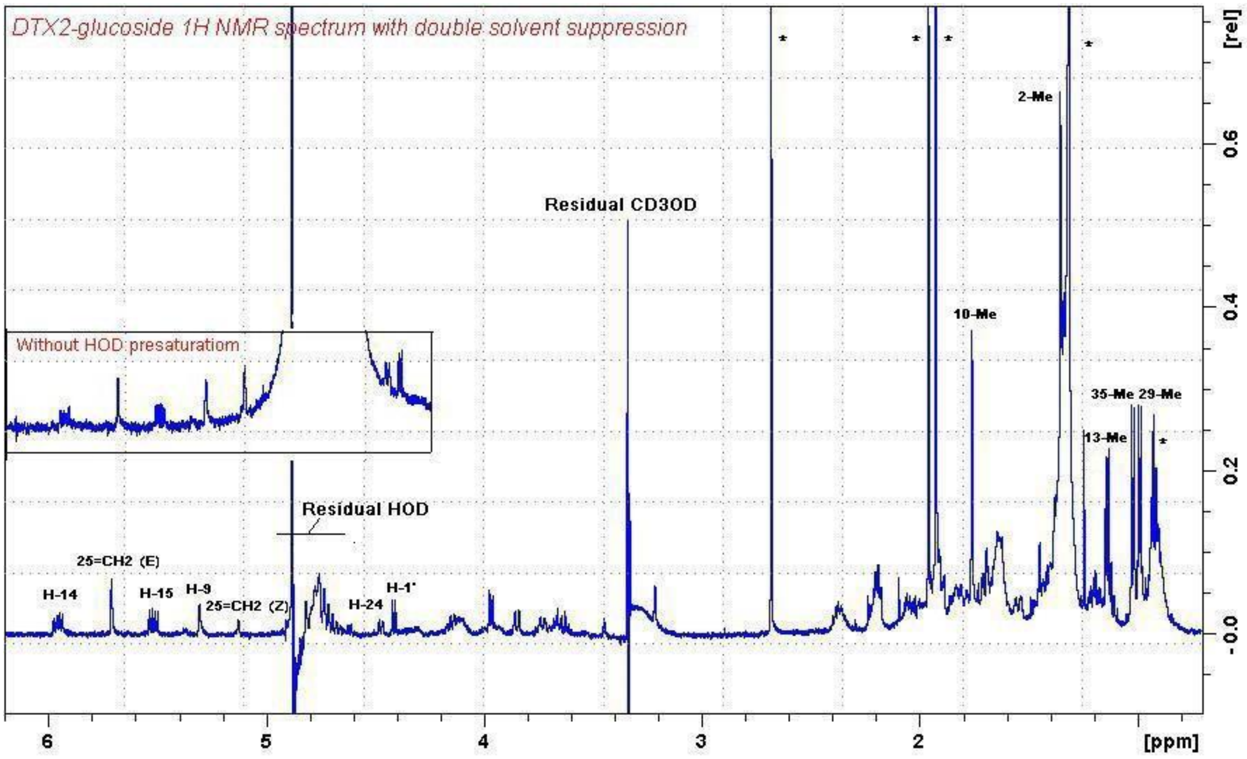


**Figure S20.** $^{1}$H NMR spectrum of DTX2-glc (**5**) in $CD_3OD$ at 600 MHz. Major impurity peaks are marked with asterisks. The inset shows the low-field region without solvent suppression.

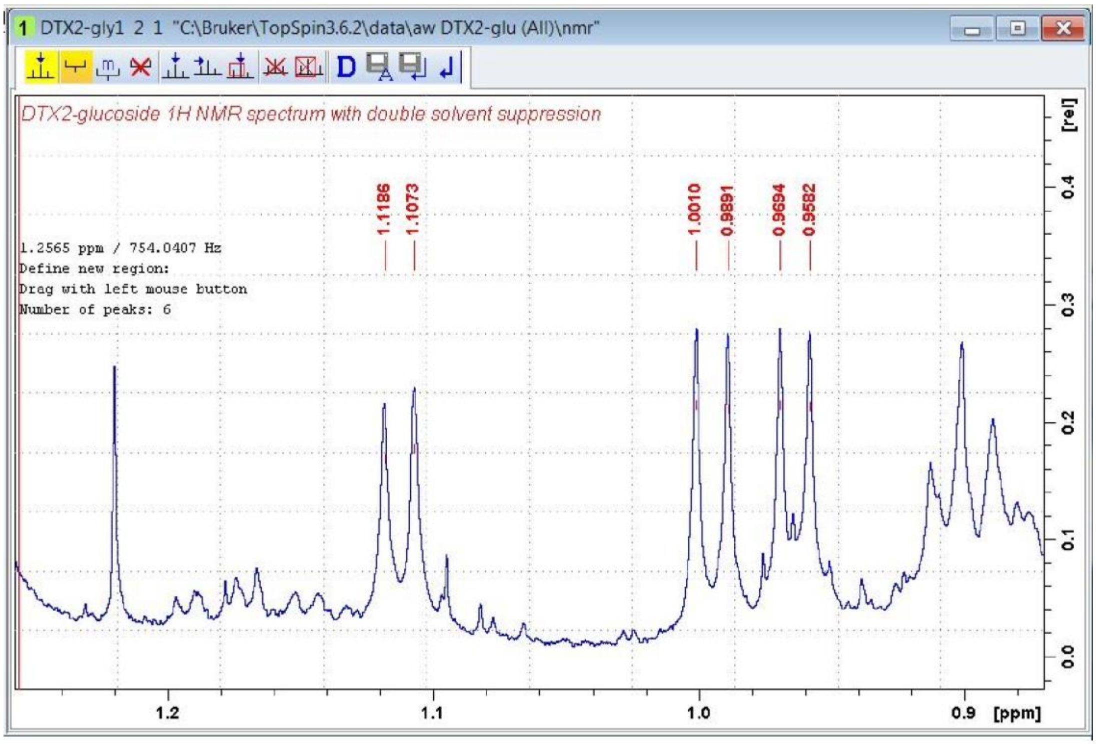


**Figure S21.** Expansion of the $^{1}$H NMR spectrum of DTX2-glc (**5**) (Figure S20) showing the secondary methyl region of **5**.

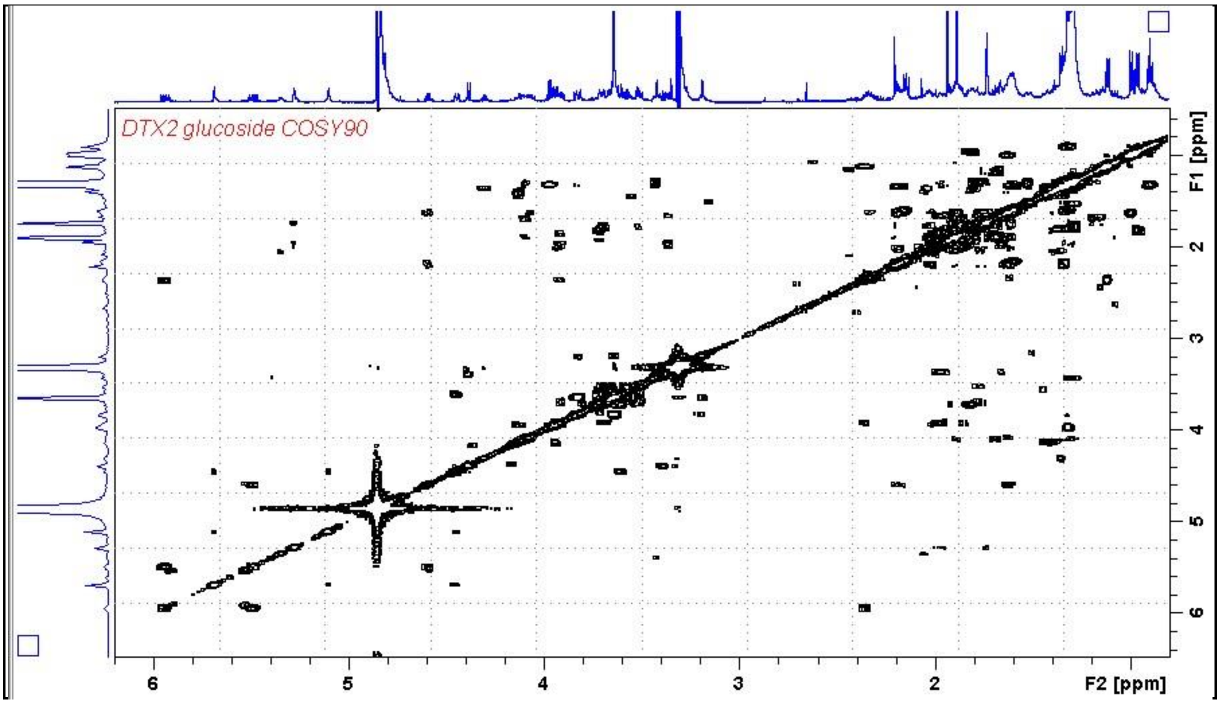


**Figure S22.** COSY NMR spectrum of DTX2-glc (**5**) in $CD_3OD$ at 600 MHz.

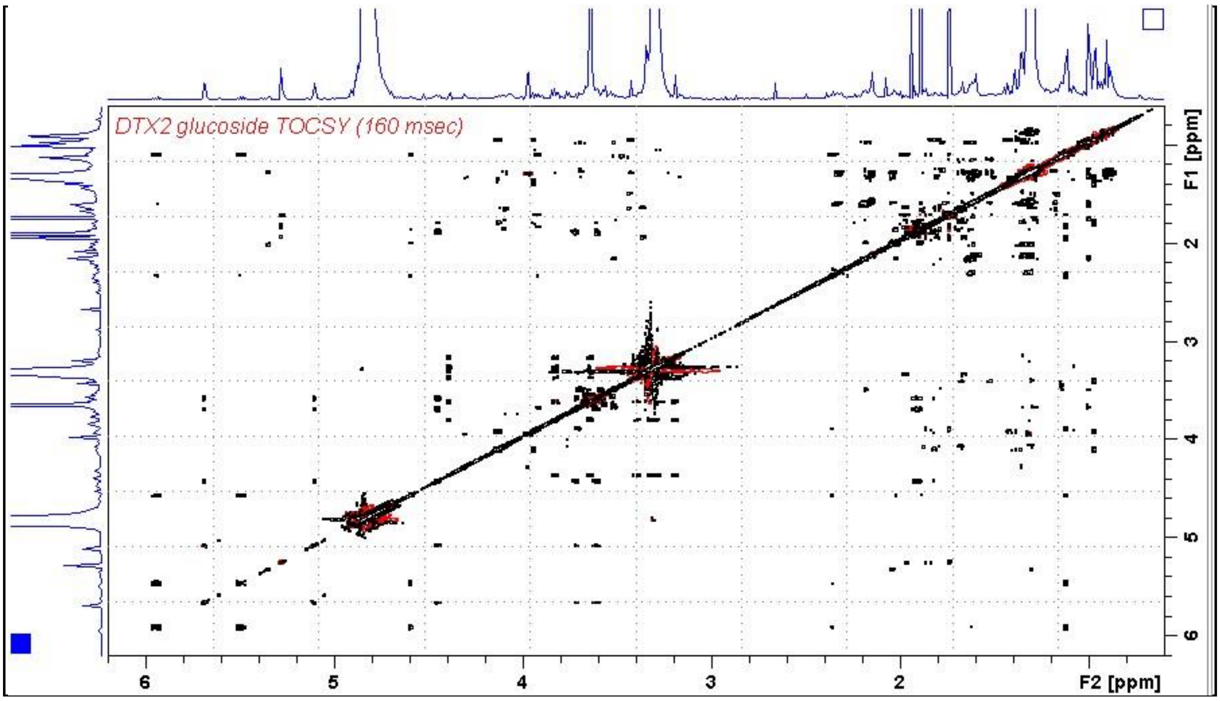


**Figure S23.** TOCSY NMR spectrum of DTX2-glc (**5**) in $CD_3OD$ at 600 MHz.

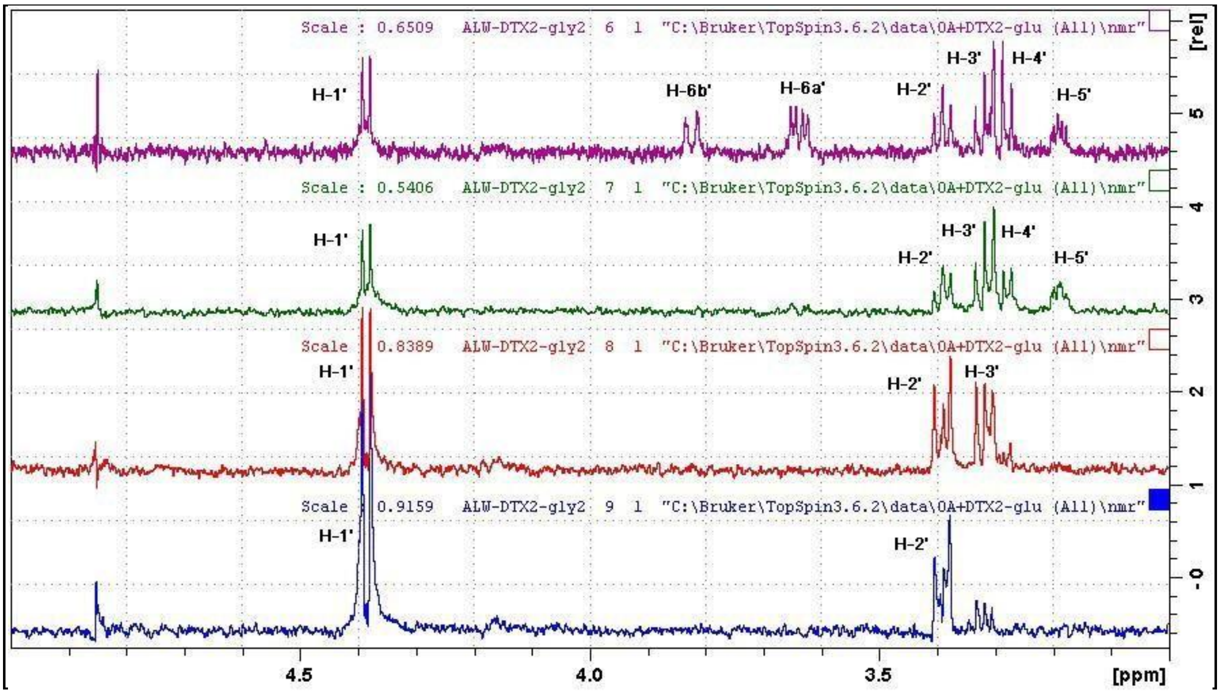


**Figure S24.** 1D-SELTOCY (d9 = 30, 50, 80, and 160 ms (from bottom to top)) NMR spectra at 600 MHz determined for H-1′ of DTX2 24-*O*-β-D-glucoside (**5**), which identify the H-2′ to H-6′ signals of the glucoside unit.

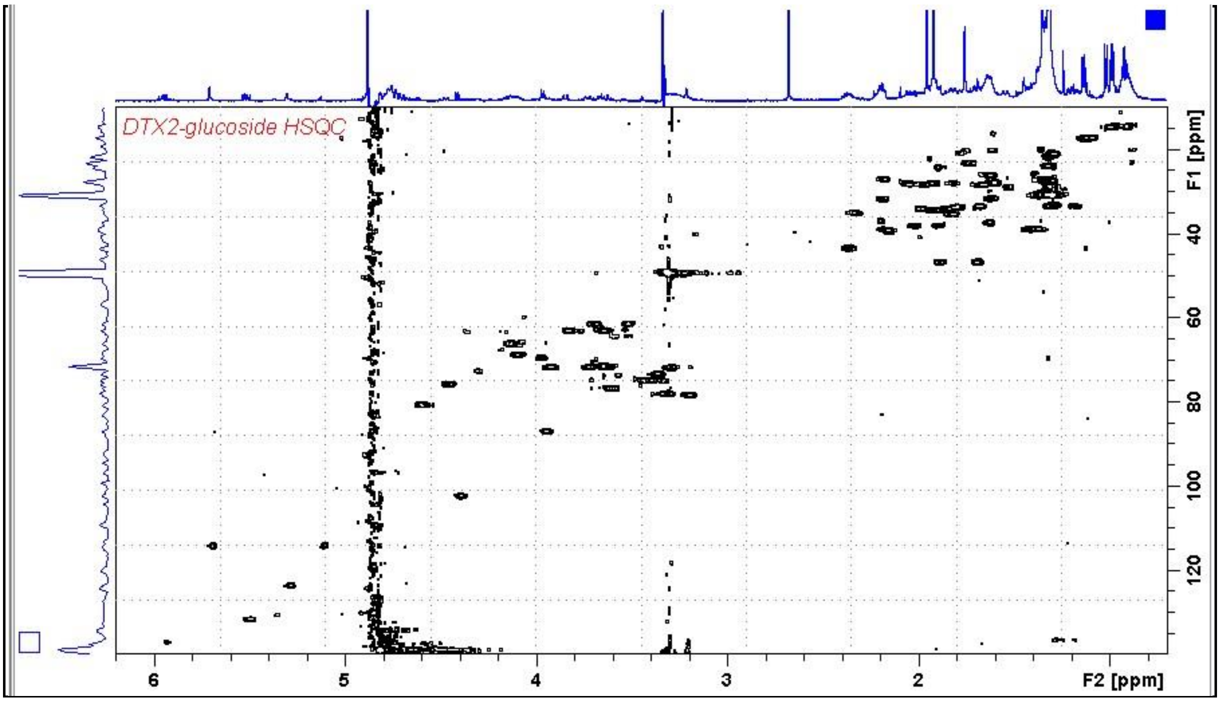


**Figure S25.** HSQC NMR spectrum of DTX2-glc (**5**) in $CD_3OD$.

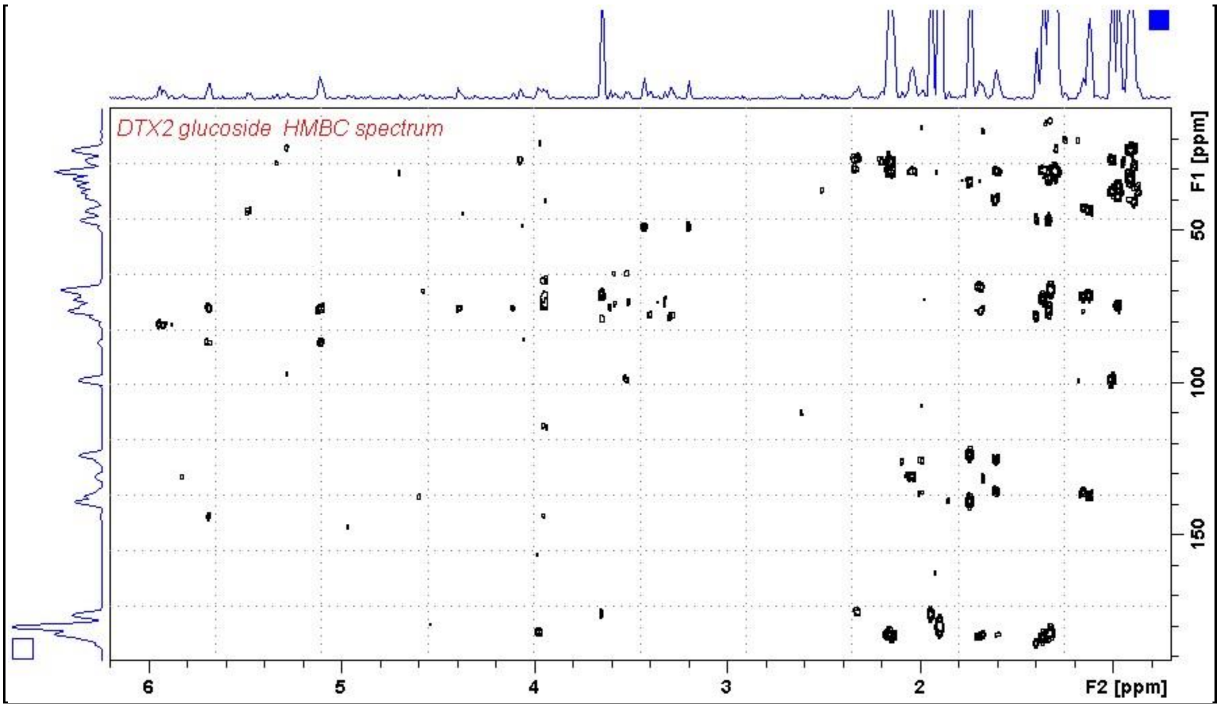


**Figure S26.** HMBC NMR spectrum of DTX2-glc (**5**) in $CD_3OD$.

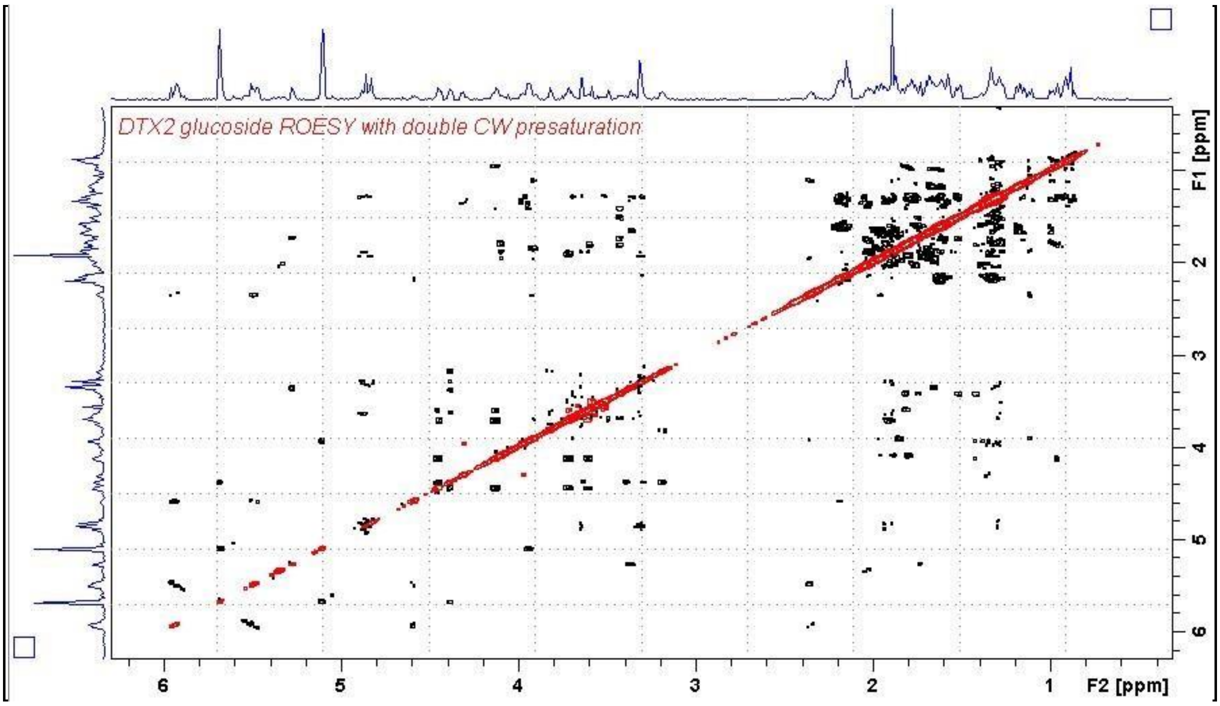


**Figure S27.** ROESY NMR spectrum of DTX2-glc (**5**) in $CD_3OD$.

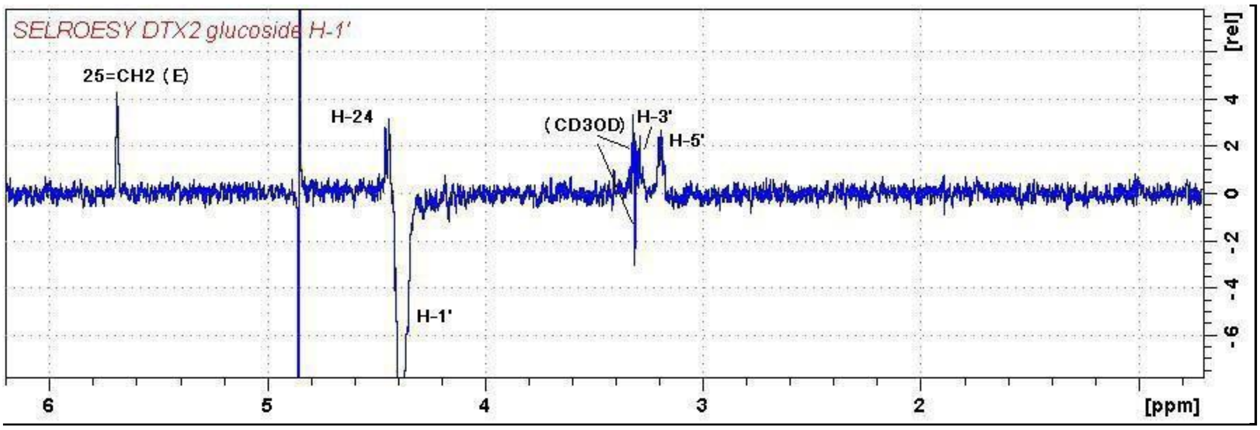


**Figure S28.** 1D-SELROESY NMR spectrum for H-1′ of DTX2-glc (**5**) in $CD_3OD$.

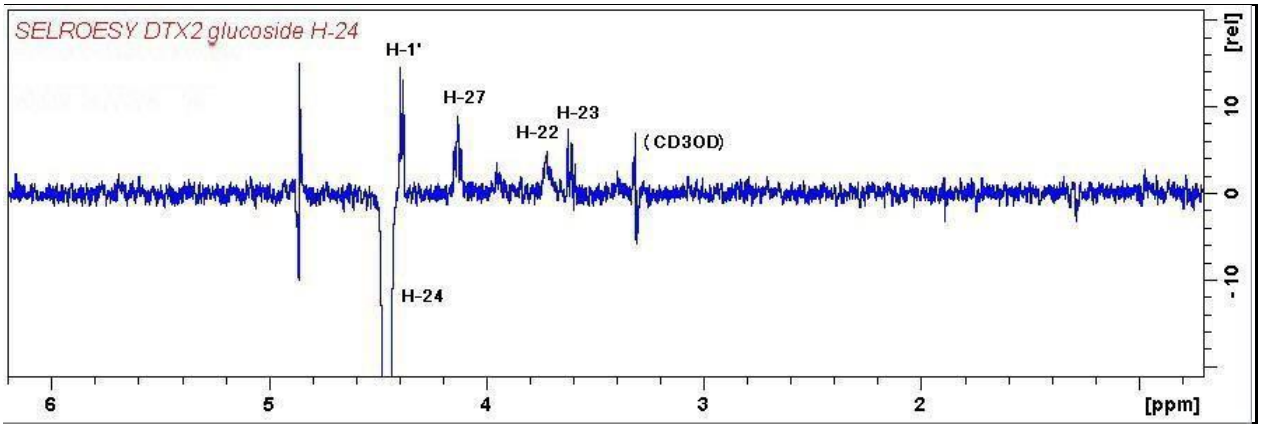


**Figure S29.** 1D-SELROESY NMR spectrum for H-24 of DTX2-glc (**5**) in $CD_3OD$.

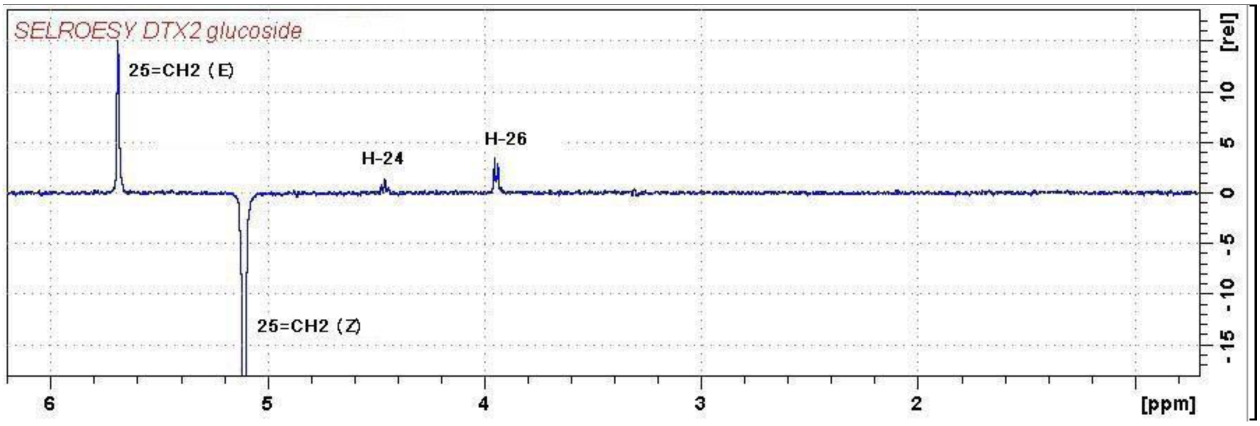


**Figure S30.** 1D-SELROESY NMR spectrum for 25=$CH_2$ (*Z*) of DTX2-glc (**5**) in $CD_3OD$.

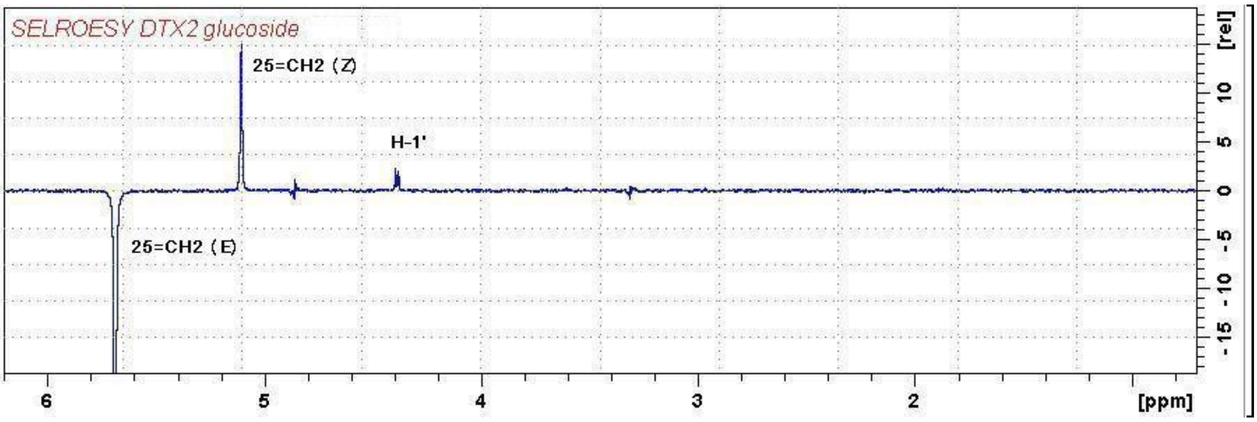


**Figure S31.** 1D-SELROESY NMR spectrum for 25=$CH_2$ (*E*) of DTX2-glc (**5**) in $CD_3OD$.

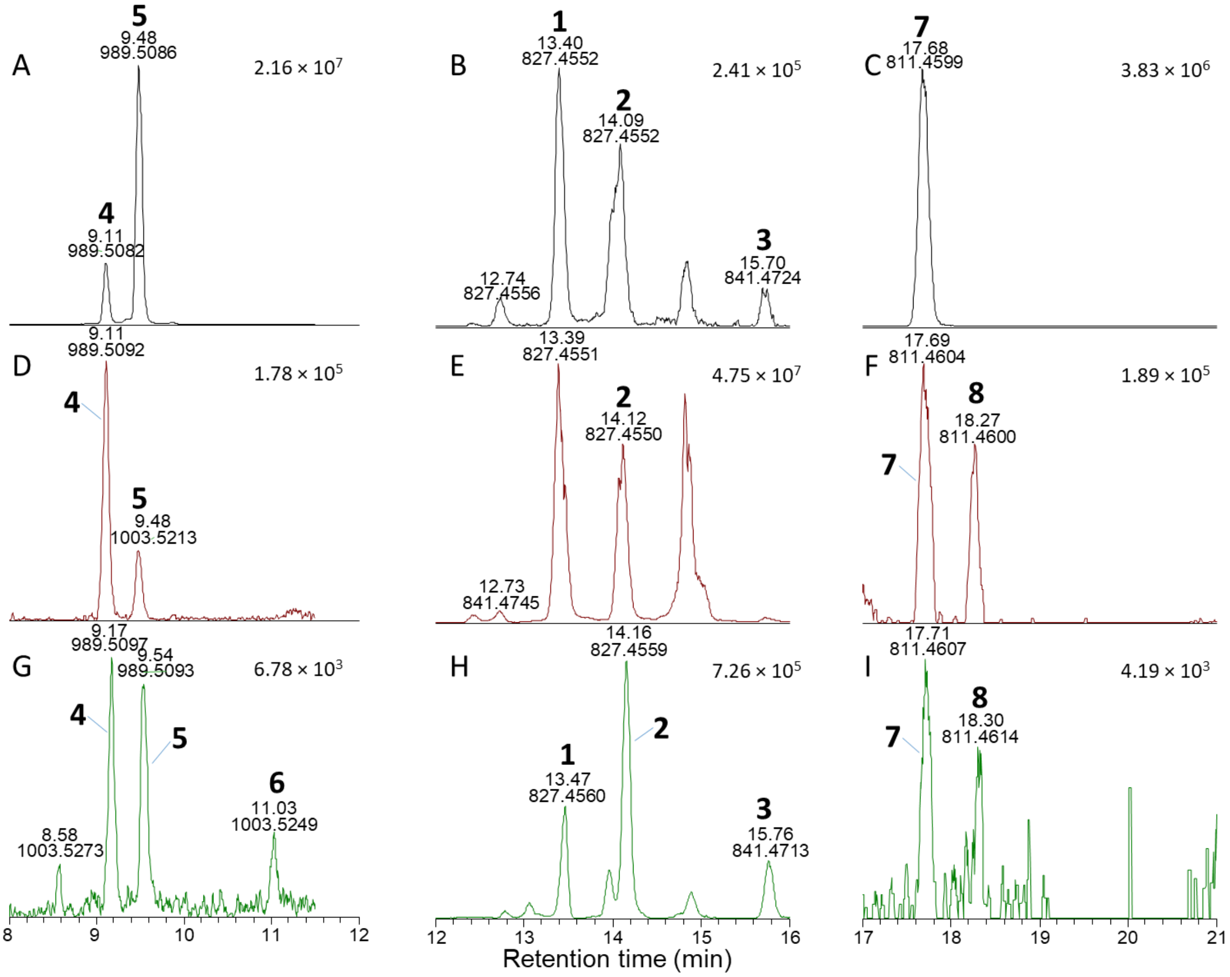


**Figure S32.** Positive-ionization mode extracted-ion (±5 ppm) SIM chromatograms from LC– HRMS analysis (method B) of: the mixed standard of **4**, **5**, and **7** containing **1–3** as minor contaminants (A–C); an extract of a *D. acuta* culture VGO1065 (D–F), and; the SPE concentrate from CRM-FDMT1 (G–I). The chromatograms in panels A, D, and G were extracted at *m*/*z* 989.5080 and 1003.5237 (*m*/*z* of $[M+Na]^+$ for **4–6**), those in panels B, E, and H at *m*/*z* 827.4552 and 841.4709 (*m*/*z* of $[M+Na]^+$ for **1–3**), and in panels C, F, and I at *m*/*z* 811.4603 and 825.4759 (*m*/*z* of $[M+Na]^+$ for **7–9**). Peaks are labelled with the compound numbers (Figure 1), observed retention times and *m*/*z* values, and the number in the top right-hand corner of each panel indicates the intensity (counts) of the highest peak. A corresponding set of peaks was also detected in the negative ionization chromatograms (e.g., see Figure 3).

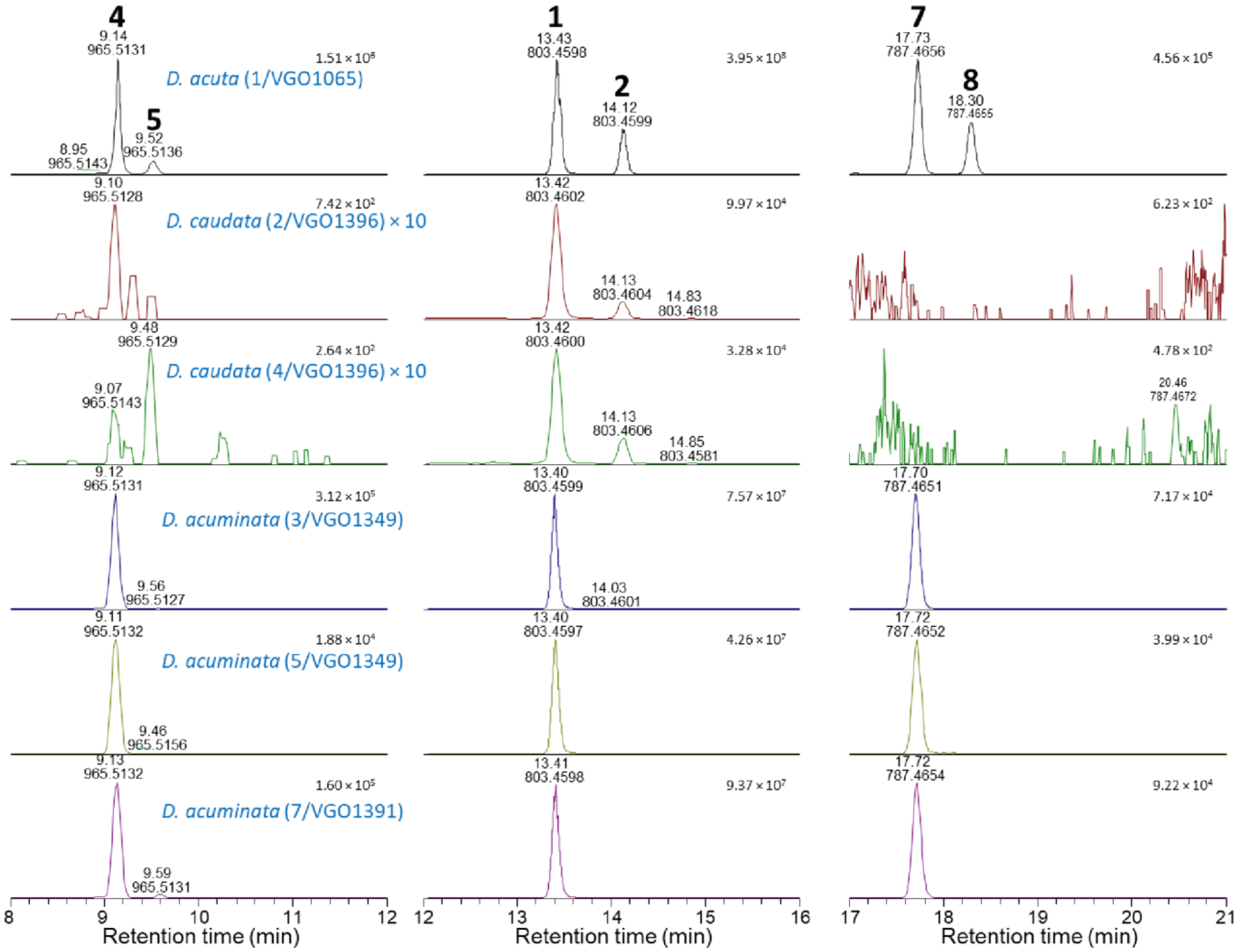


**Figure S33.** Negative ionization mode LC–HRMS SIM chromatograms (method B) of HP-20 extracts of *Dinophysis* cultures (Table S3) extracted at (±5 ppm, from left to right) *m*/*z* 965.5115 (**4**, **5**), 803.4587 (**1**, **2**), and 787.4638 (**7**, **8**). Comparisons with standards (**1**, **2**, **3**, **4**, **5**, and **7**) and an SPE concentrate of CRM-FDMT1 are shown in Figure 3.

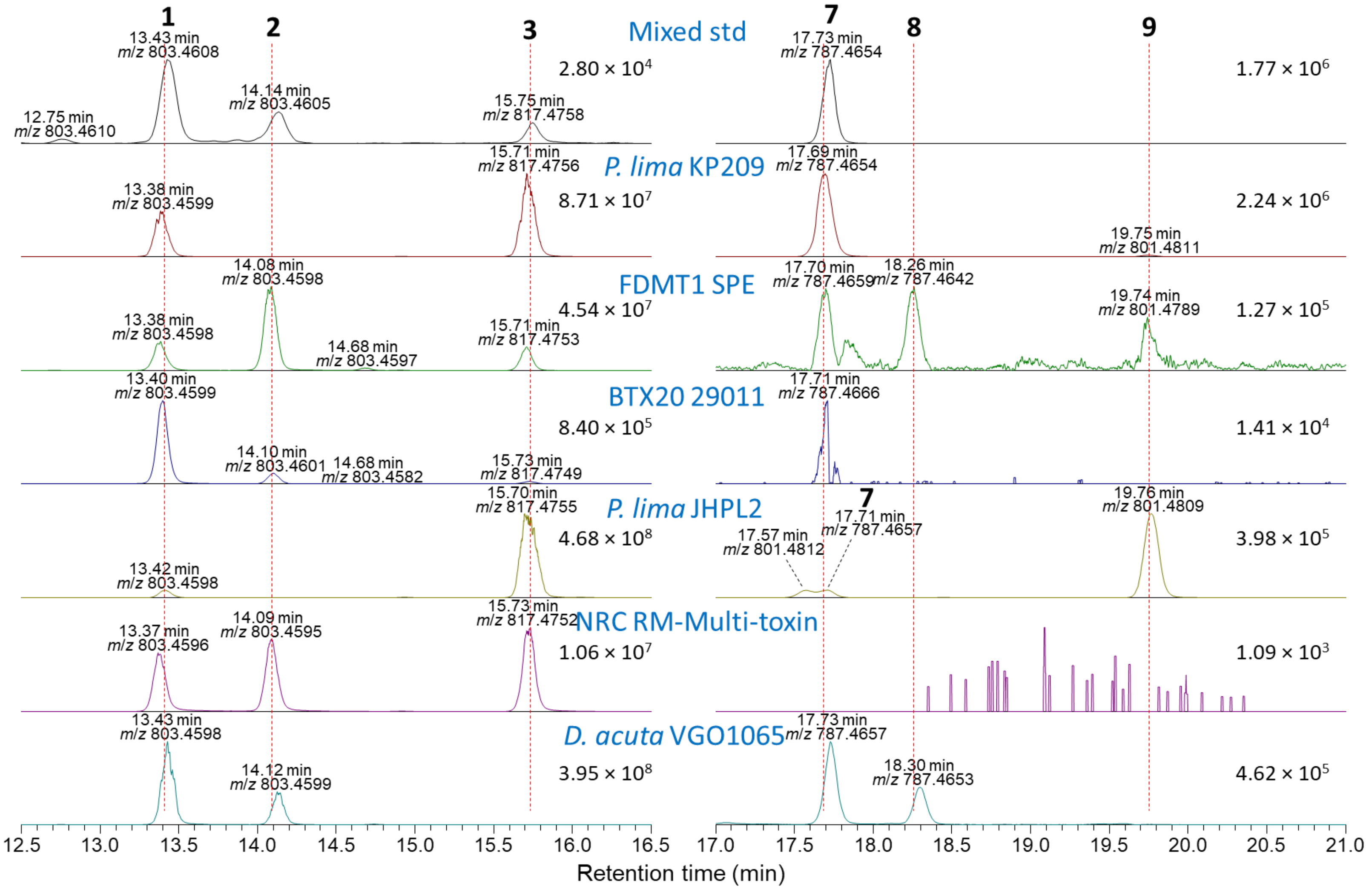


**Figure S34.** Negative mode LC–HRMS SIM chromatograms (method B) of the mixed standard of **4**, **5**, and **7** (top) and a selection of reference materials, shellfish extracts, and algal cultures extracted at the exact masses (±5 ppm) of **1–3** (left panel) and **7–9** (right panel).

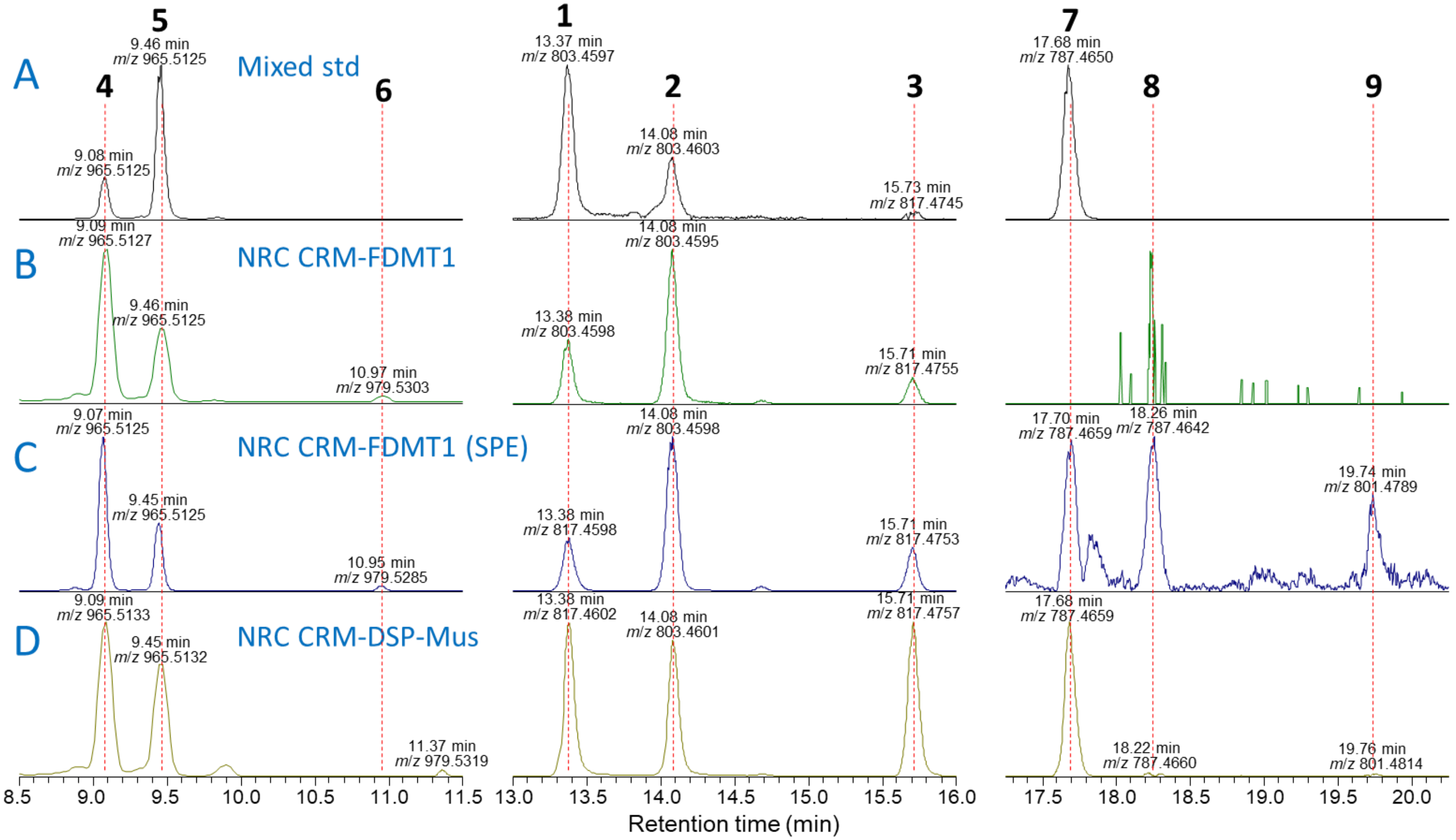


**Figure S35.** Negative mode LC–HRMS SIM chromatograms (method B) of: A, the mixed standard of **4**, **5**, and **7**; B, an extract of NRC CRM-FDMT1; C, an SPE concentrate of the extract of NRC CRM-FDMT1, and; D, and extract of NRC CRM-DSP-Mus-c. Chromatograms are extracted at the exact masses of **4–6** (left-hand panels), **1–3** (centre panels), and **7–9** (right-hand panels).

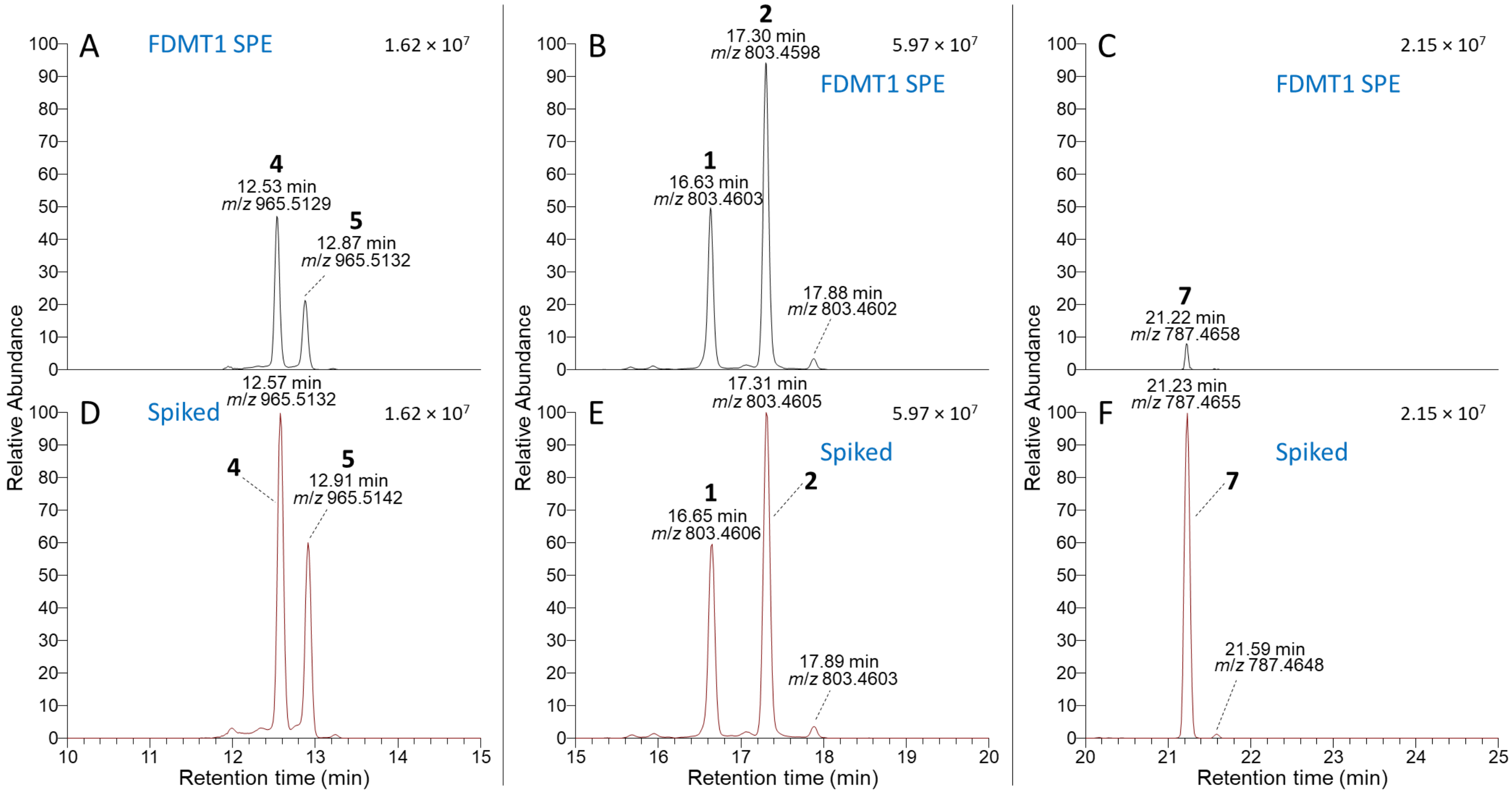


**Figure S36.** Negative mode full scan LC–HRMS (method C) chromatograms showing the CRM-FDMT1 SPE-concentrated extract (top), and the same extract after spiking with standards of **4**, **5**, and **7** (bottom). The segment at 10–15 min was extracted at *m*/*z* 965.5115, 15–20 min at *m*/*z* 803.4587, and 20–25 min at *m*/*z* 787.4638 (all at ±5 ppm). Each segment of the two chromatograms is displayed with the same absolute vertical scale to show the change in peak intensities of **4**, **5**, and **7** after spiking, relative to **1** and **2** (which were only present as trace contaminants in the standards used for spiking).

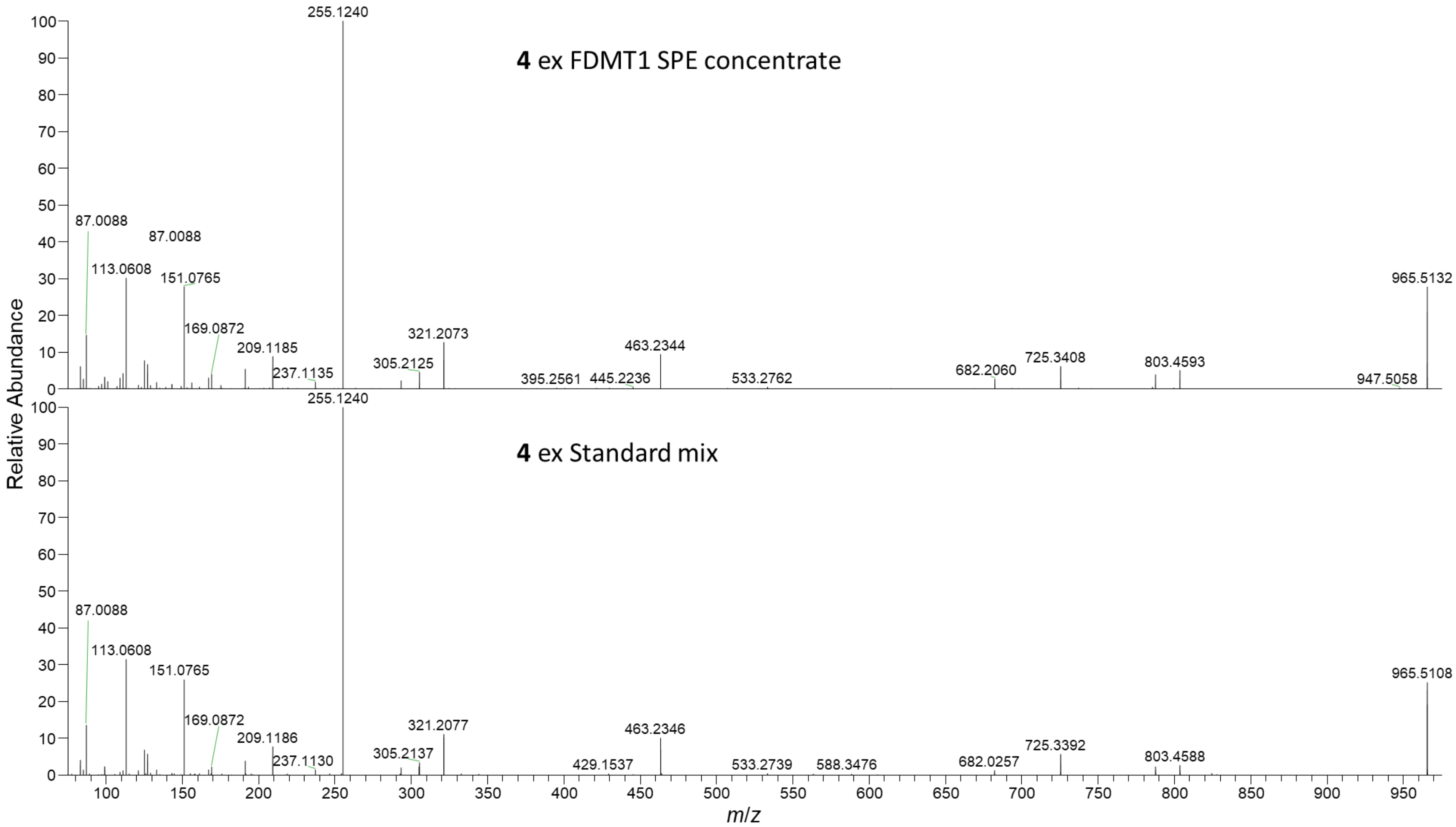


**Figure S37.** Negative mode LC–HRMS/MS PRM spectra (method C) of [M−H]⁻ of **4** in: top, the FDMT1 SPE concentrate, and; bottom, in the mixed standard of **4**, **5**, and **7**.

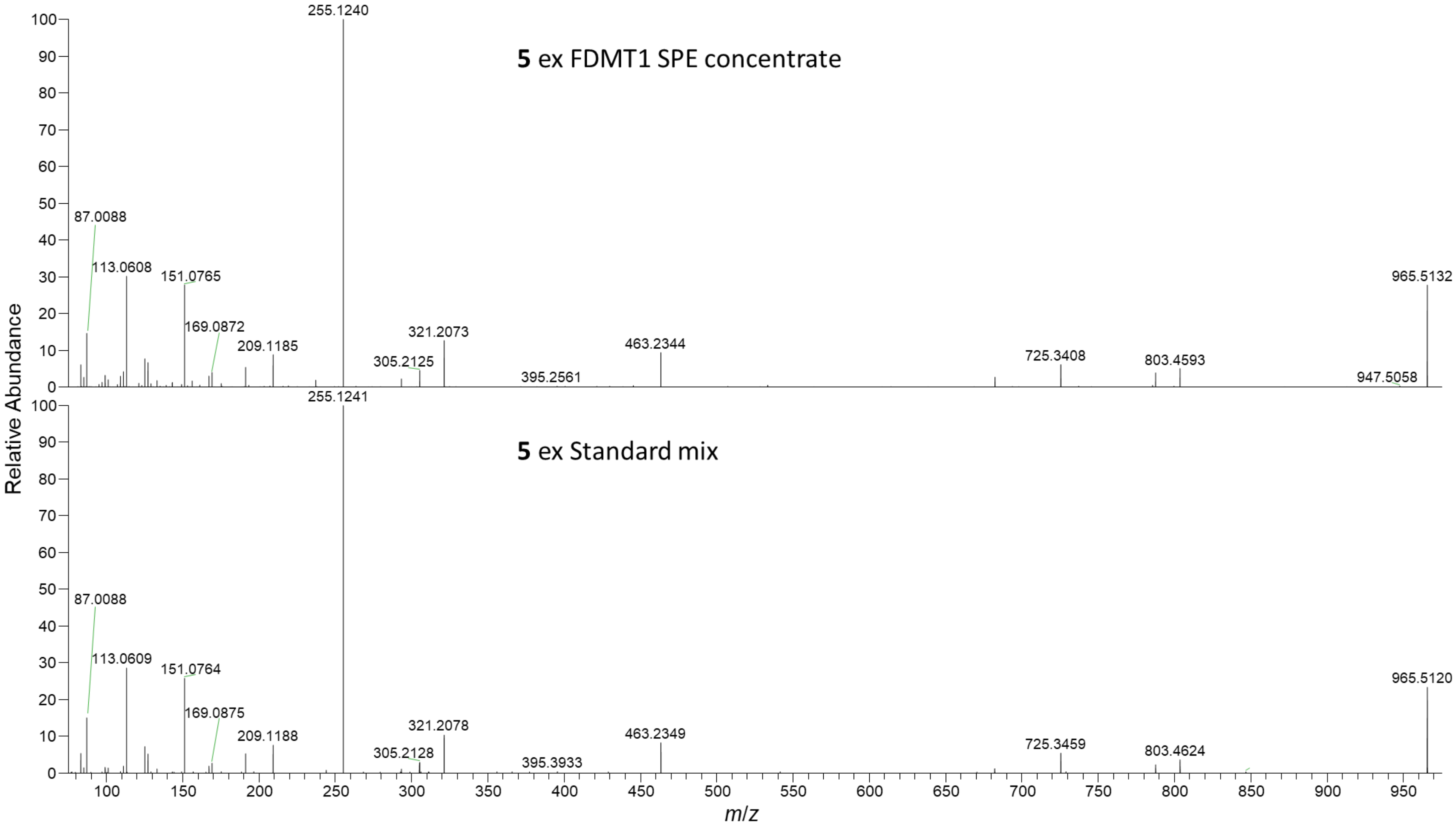


**Figure S38.** Negative mode LC–HRMS/MS PRM spectra (method C) of [M−H]$^-$ of **5** in: top, the FDMT1 SPE concentrate, and; bottom, in the mixed standard of **4**, **5**, and **7**.

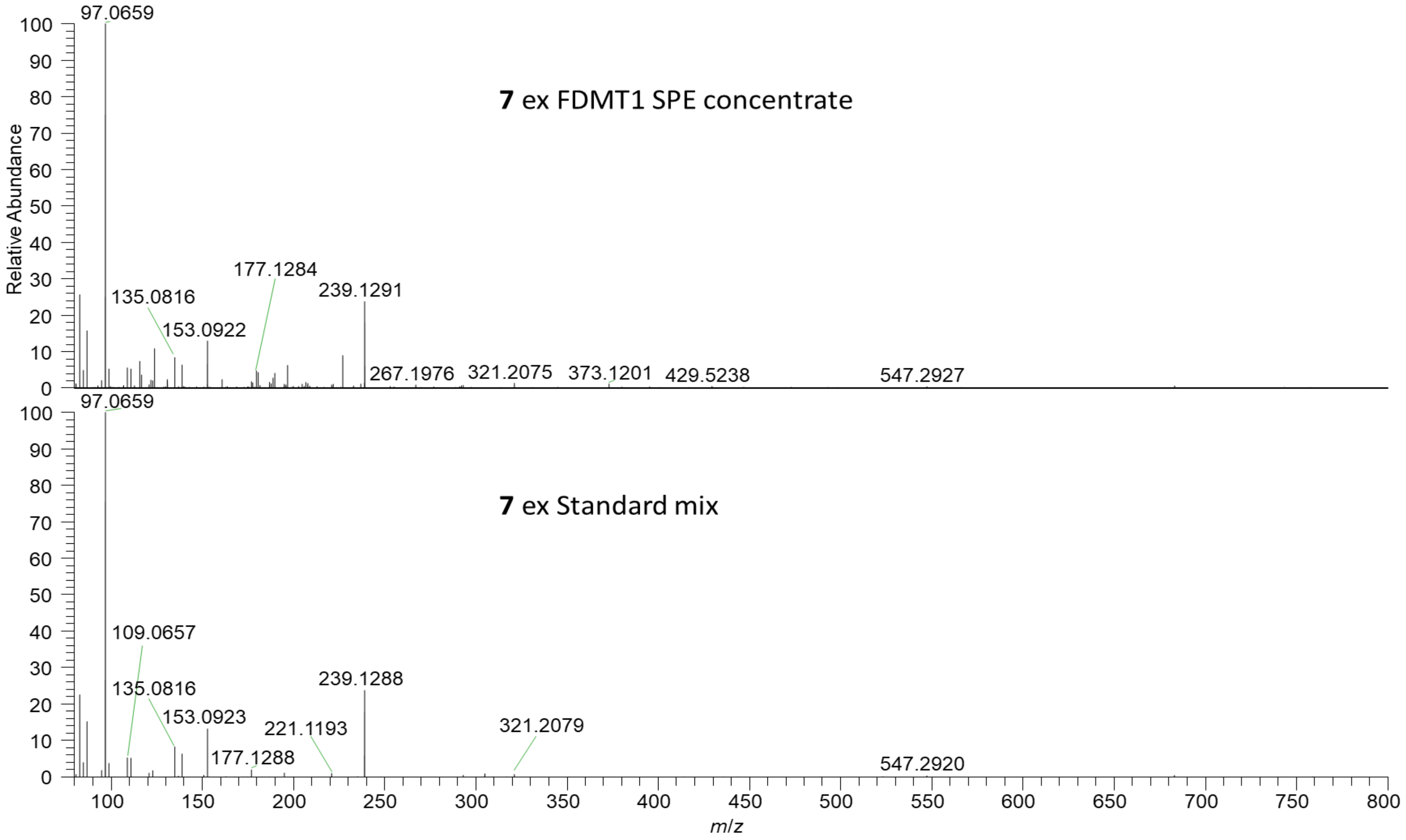


**Figure S39.** Negative mode LC–HRMS/MS PRM spectra (method C) of [M−H]⁻ of **7** in: top, the FDMT1 SPE concentrate, and; bottom, in the mixed standard of **4**, **5**, and **7**. Some extraneous peaks are visible in the spectrum obtained from FDMT1 due to the presence of components from the blue mussel matrix and the lower signal-to-noise for **7** in this sample.

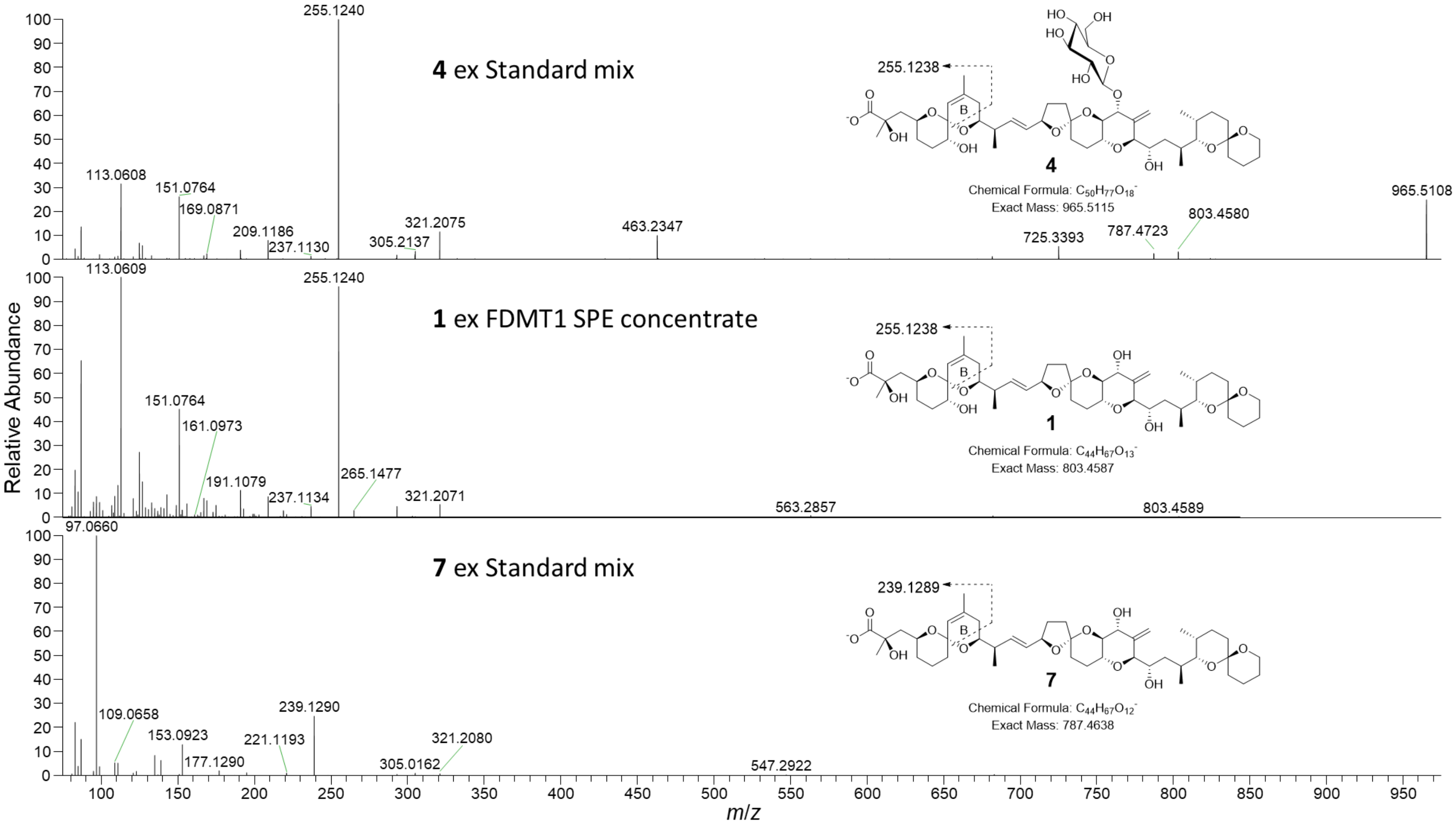


**Figure S40.** Negative mode LC–HRMS/MS PRM spectra (method C) of [M−H]⁻ of top, **4** in the mixed standard of **4**, **5**, and **7**; middle, **1** in the FDMT1 SPE concentrate, and; bottom, **7** in the mixed standard of **4**, **5**, and **7**. The PRM spectra were all acquired with the same collision energy (70 eV), and show a marked difference in the abundance of the B-ring retro-Diels–Alder cleavage (marked on structure) product ion (*m*/*z* 255.1238 for **1** and **4**, *m*/*z* 239.1289 for **7**) relative to the other product ions.

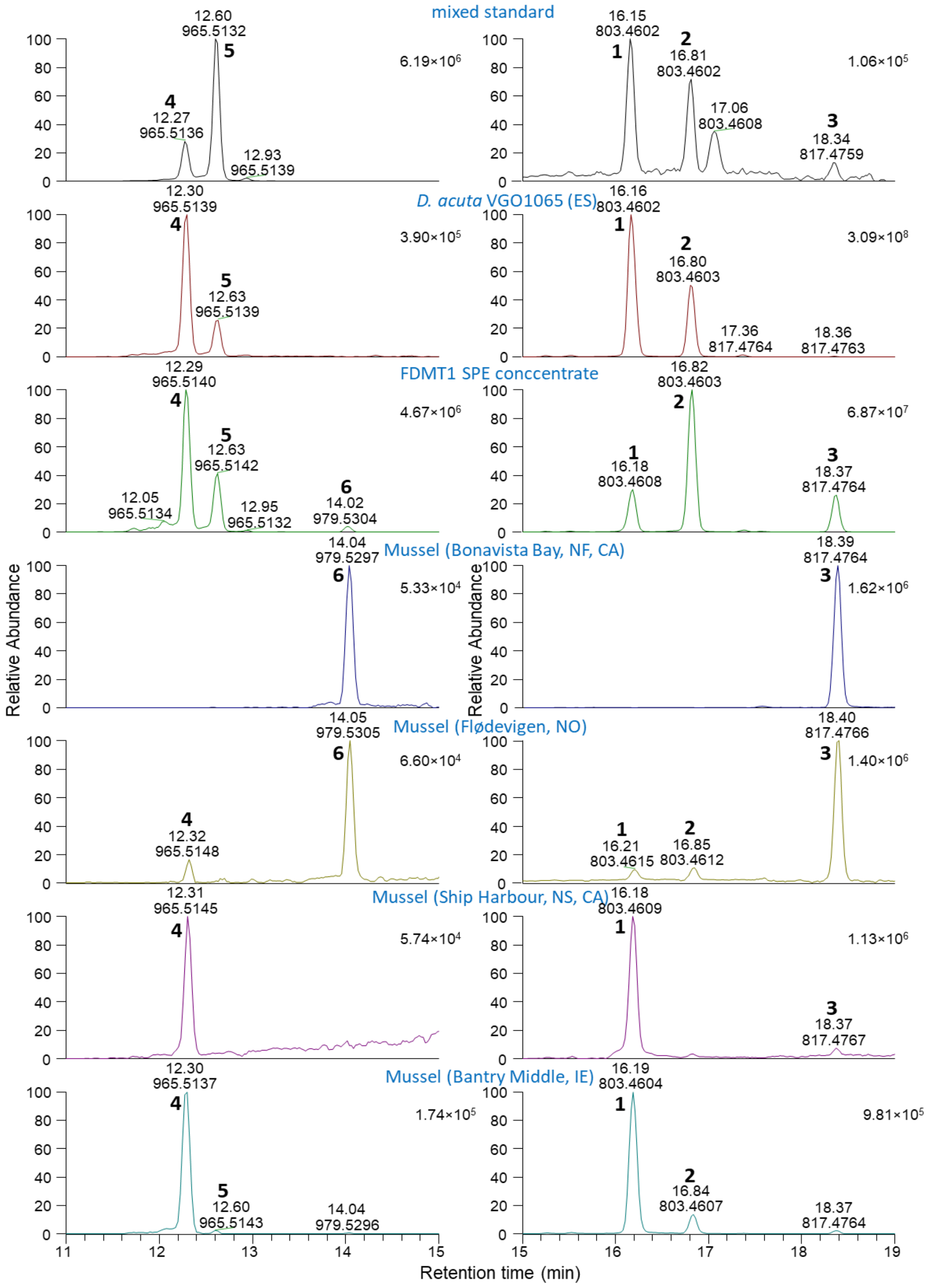


**Figure S41.** Full scan negative mode LC–HRMS (method C, FS/AIF) chromatograms from a range of samples. The left-hand segments at 11–15 min were extracted at *m*/*z* 965.5115 and 979.5272 (for **4**–**6**), and the right-hand segments at 15–19 min were extracted at *m*/*z* 803.4587 and 817.4744 (for **1**–**3**) (all at ±5 ppm). Peaks are labelled with their base-peak *m*/*z*, retention time (min), and compound-number (Figure 1).

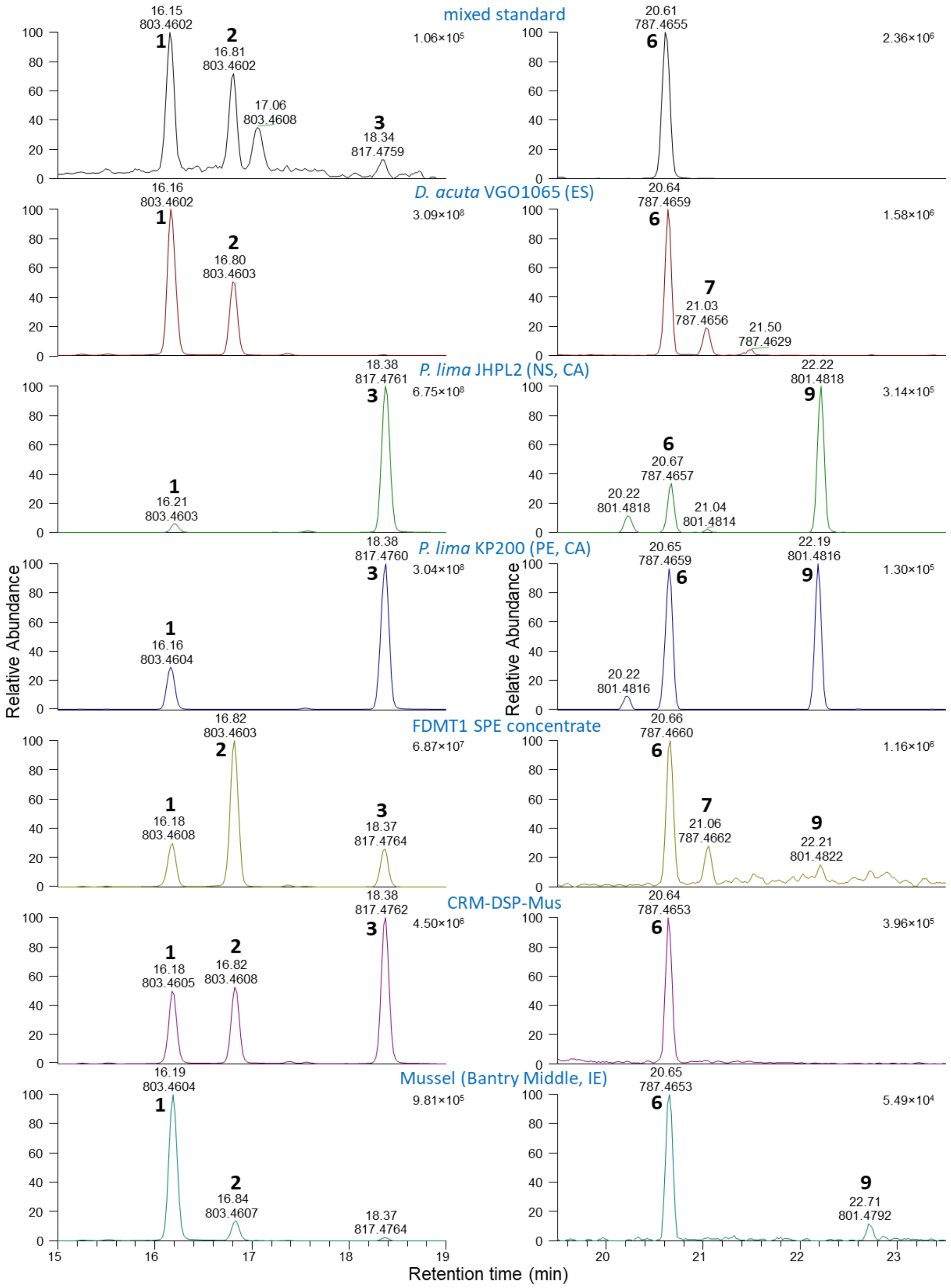


**Figure S42.** Full scan negative mode LC–HRMS (method C, FS/AIF) chromatograms from a range of samples. The left-hand segments at 15–19 min were extracted at *m*/*z* 803.4587 and 817.4744 (for **1**–**3**), and the right-hand segments at 19.5–23.5 min were extracted at *m*/*z* 787.4638 and 801.4794 (for **7**–**9**) (all at ±5 ppm). Peaks are labelled with their base-peak *m*/*z*, retention time (min), and compound-number (Figure 1).

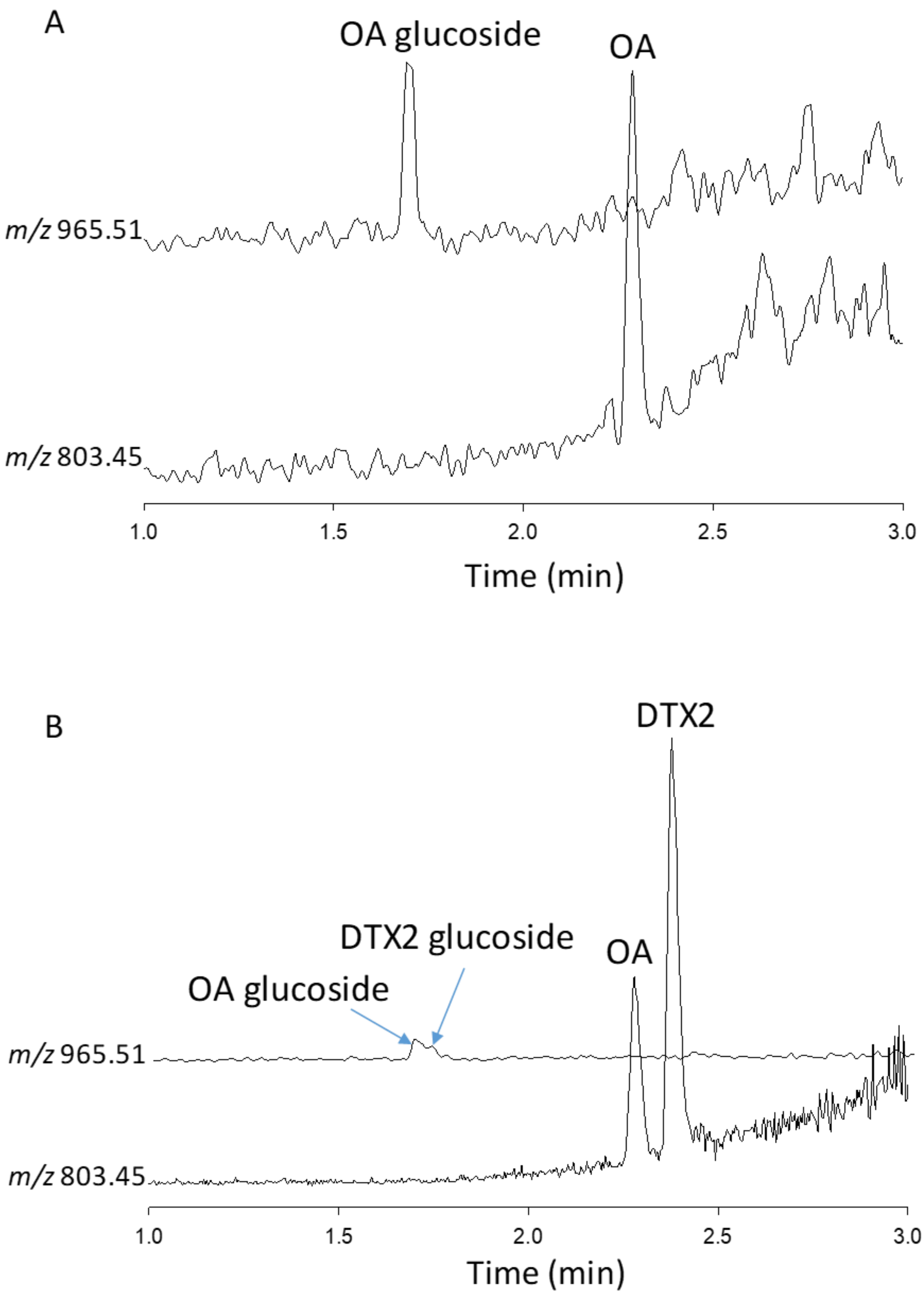


**Figure S43.** LC–HRMS analysis (method D) of: A, *M. edulis* (Castlemaine Harbour, 13/07/20) and; B, *M. edulis* (Bantry North, 31/08/20) samples containing OA (**1**), DTX2 (**2**), and their glucosides (**4** and **5**) (see Table 5).

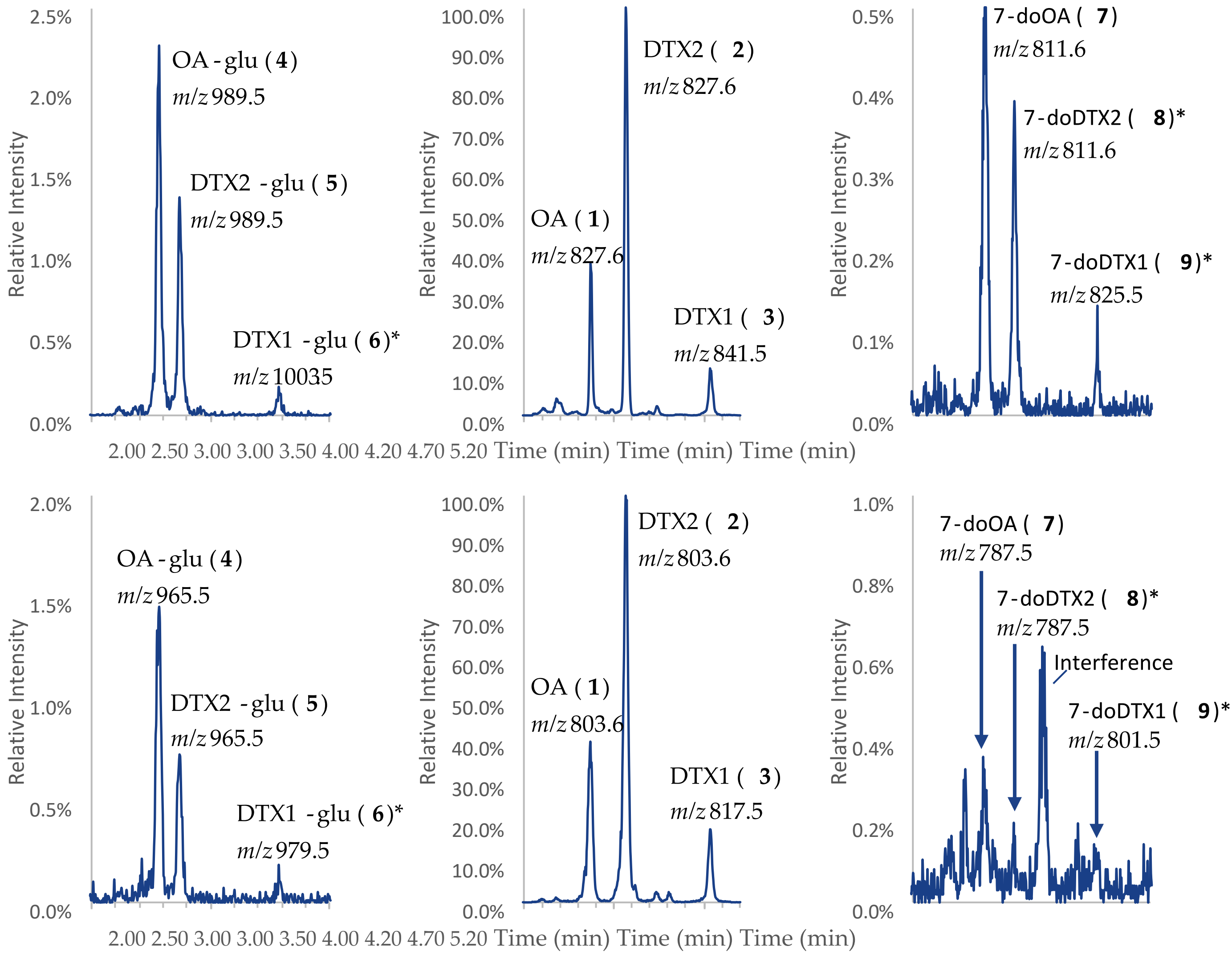


**Figure S44.** Total ion chromatograms from the NRC CRM-FDMT1 extract using LC–MS/MS (method E) in: positive ion mode (top), and; negative ion mode (bottom). *Indicates peak whose identity has not been confirmed with this analytical method.

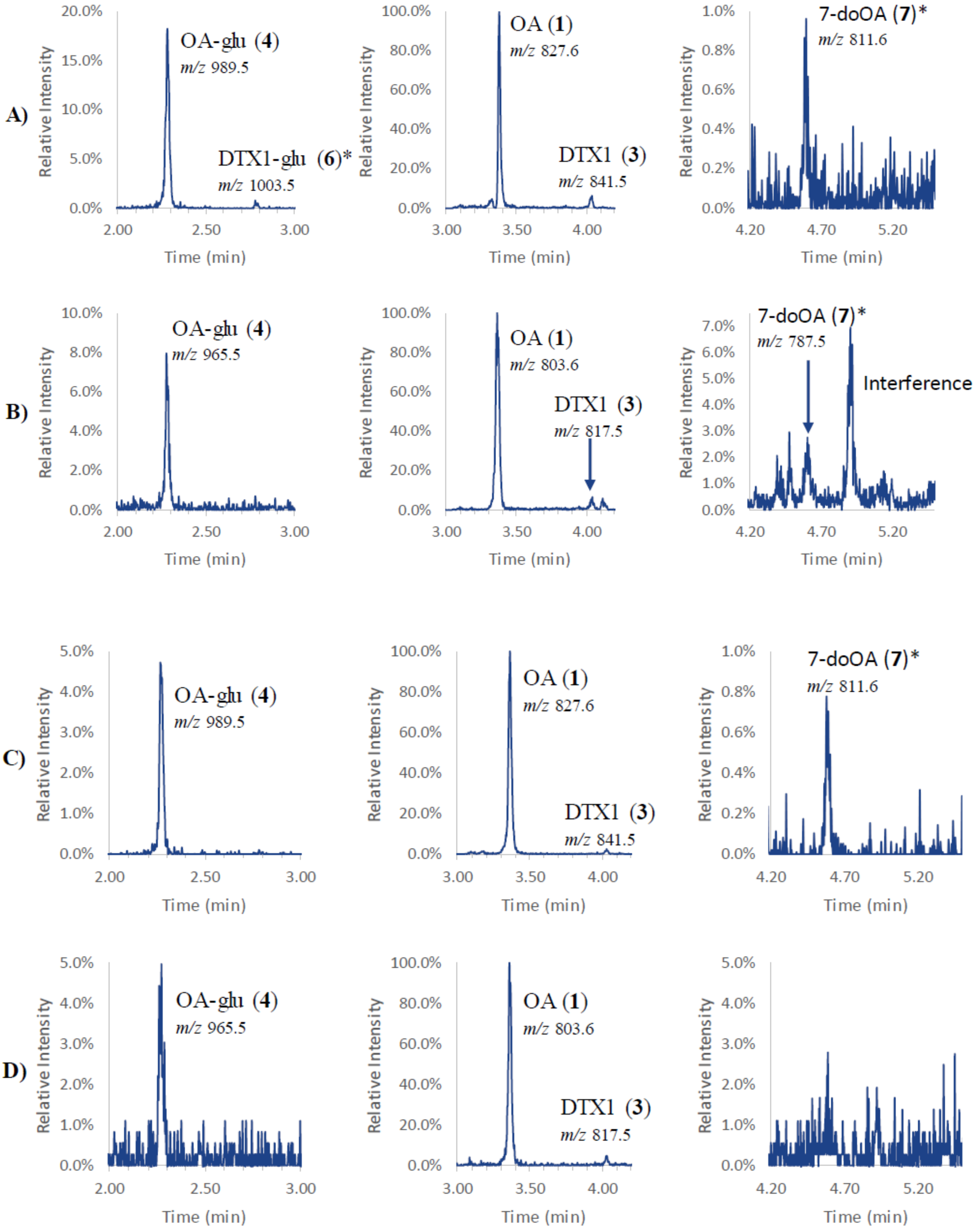


**Figure S45.** Total ion chromatograms obtained using LC–MS/MS (method E) on *M. edulis* from Sumner, New Zealand, on 04/01/2017 from: A, the non-hydrolyzed extract in positive, and; B, negative ion modes, and; C, the hydrolyzed extract in positive, and; D, negative ion modes. Possible traces of DTX1-glu (**6**) and 7-deoxyOA (**9**) close to the limit of detection are marked with asterisks.

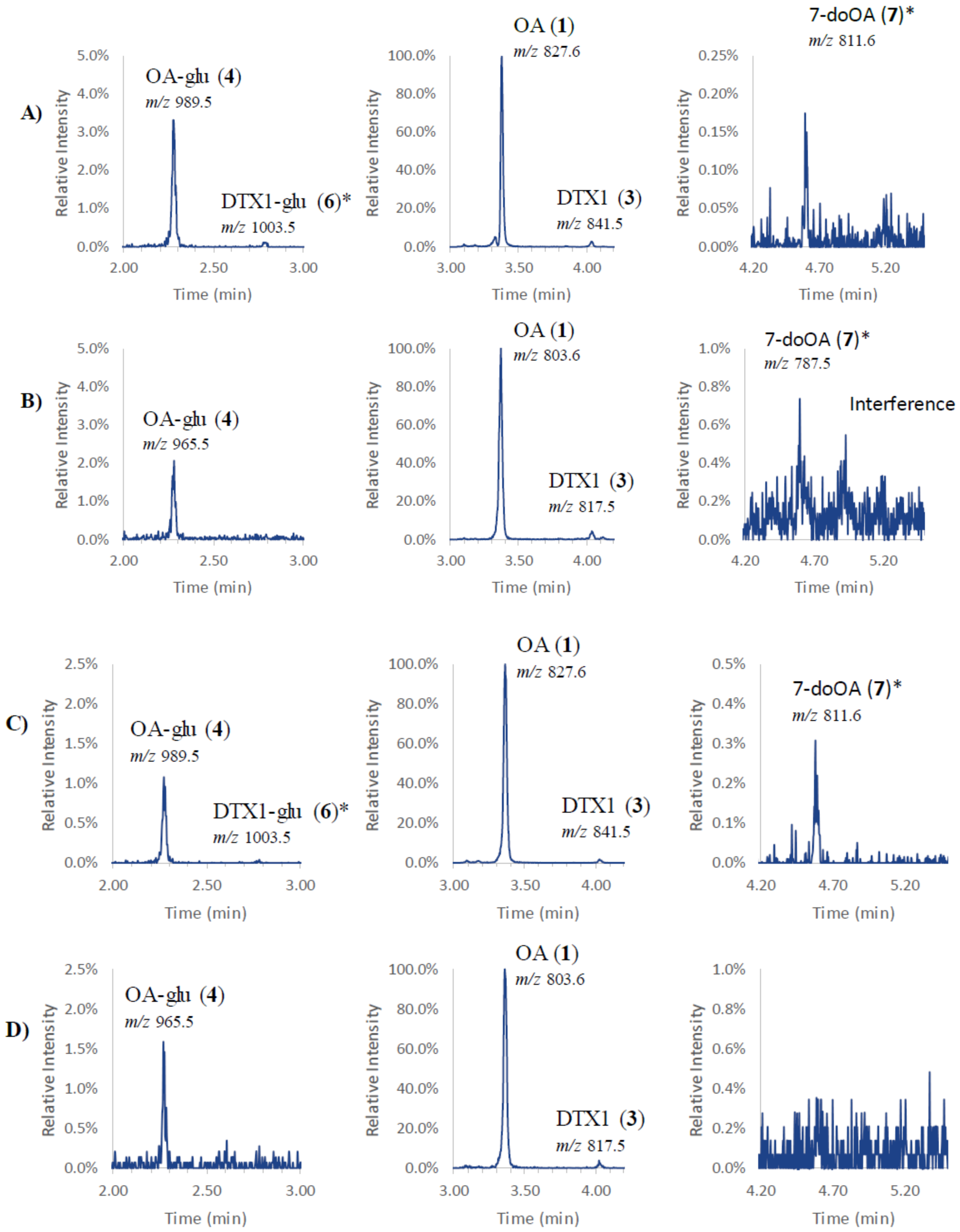


**Figure S46.** Total ion chromatograms obtained using LC–MS/MS (method E) on *M. edulis* from Akaroa Harbour, New Zealand, on 07/08/2017 from: A, the non-hydrolyzed extract in positive, and; B, negative ion modes, and; C, the hydrolyzed extract in positive, and; D, negative ion modes. Possible traces of DTX1-glu (**6**) and 7-deoxyOA (**9**) close to the limit of detection are marked with asterisks.

**Table S1.** Details of *Dinophysis* cultures extracted for analysis in this study.

| Label/accession number | Species | Volume (mL) | Yield (cells/mL) | Number of cells |
|---|---|---|---|---|
| VGO 1065 | *D. acuta** | N/A* | N/A* | 9,226,000 |
| 2/VGO 1396 | *D. caudata* | 1,084 | 3,000 | 3,252,000 |
| 3/VGO 1349 | *D. acuminata* | 4,500 | 1,407 | 6,331,500 |
| 4/VGO 1396 | *D. caudata* | 650 | 931 | 605,150 |
| 5/VGO 1349 | *D. acuminata* | 2,700 | 321 | 866,700 |
| 7/VGO 1391† | *D. acuminata* | 17,910 | 714 | 12,787,740 |

* Grown in eight separate vessels, and the extracts combined. †Grown in a photobioreactor without aereation, as described in Hernandez-Urcera et al 2018 [2].

**Table S4.** Relative abundances expressed as a percentage of the total **1–9** detected, and total concentrations of **1–9** (ng/mL), by LC–HRMS (method D) in stored extracts of SPATT discs deployed in Irish waters by Fux *et al*. [3, 4] in 2005–2006[a].

| Location | Date[b] | OA (**1**) | DTX2 (**2**) | OA-Glc (**4**) | Conc. |
|---|---|---|---|---|---|
| Bruckless | 25/08/05 | 89.3 | 7.1 | 3.6 | 2030 |
| Bruckless | 24/05/06 | 94.2 | 5.8 | ND | 380 |
| Bantry Bay | 12/10/05 | 84.2 | 15.8 | ND | 455 |
| Bantry Bay | 07/09/05 | 62.7 | 34.7 | 2.7 | 2948 |
| Bantry Bay | 12/10/05 | 79.4 | 20.6 | ND | 613 |

[a]Assuming identical relative responses. No DTX1 (**3**), glucosides of DTX1 and DTX2 (**5** and **6**), nor any 7-deoxyOA/DTXs (**7**–**9**) were detected in any of the samples. [b]Format dd/mm/yy.

**Table S5.** Concentrations of **1**–**9** (µg/g) in mussel samples (unhydrolyzed extracts), measured in negative mode full scan with LC–HRMS (method C, FS/AIF)[a]

| Sample | Country | **1** | **2** | **3** | **4** | **5** | **6** | **7** | **8** | **9** | Total |
|---|---|---|---|---|---|---|---|---|---|---|---|
| Hepatopancreas | NO | 0.022 | 0.014 | 0.028 | 0.000 | 0.004 | 0.000 | 0.000 | 0.000 | 0.000 | 0.070 |
| Whole mussel | NO | 0.028 | 0.028 | 0.316 | 0.003 | 0.000 | 0.018 | 0.000 | 0.000 | 0.000 | 0.392 |
| Indian Pt. broth | NS, CA | 0.001 | 0.000 | 0.374 | 0.000 | 0.000 | 0.008 | 0.000 | 0.000 | 0.000 | 0.384 |
| Bonavista Bay | NF, CA | 0.000 | 0.000 | 0.550 | 0.000 | 0.000 | 0.023 | 0.000 | 0.000 | 0.003 | 0.575 |
| Ship Harbour | NS, CA | 0.647 | 0.000 | 0.037 | 0.031 | 0.000 | 0.001 | 0.000 | 0.000 | 0.000 | 0.716 |
| CRM-DSP-Mus | NO/EI | 0.591 | 0.561 | 0.919 | 0.037 | 0.022 | 0.000 | 0.085 | 0.000 | 0.000 | 2.215 |
| Castlemaine Harbr | EI | 0.039 | 0.010 | 0.003 | 0.066 | 0.000 | 0.000 | 0.000 | 0.000 | 0.000 | 0.118 |
| Bantry Middle | EI | 0.496 | 0.058 | 0.008 | 0.106 | 0.000 | 0.000 | 0.023 | 0.001 | 0.001 | 0.693 |

[a]Obtained from extracted ions of the exact masses in Figure 1 (± 5 ppm). Results obtained with LC–HRMS (method B, SIM) on the same extracts are presented in Table 4.

**Table S6.** Tabulation of transitions used in MRM analyses with LC–MS/MS method E[a]

| | Negative mode | | | | | Positive mode | | |
|---|---|---|---|---|---|---|---|---|
| Analyte | Precursor | CE 1 | Product 1 | CE 2 | Product 2 | Precursor | CE | Product |
| **1** and **2** | 803.5 | 63 | 255.1 | 57 | 563.3 | 827.6 | 65 | 723.5 |
| **3** | 817.5 | 63 | 255.1 | 57 | 563.3 | 841.5 | 65 | 737.4 |
| **4** and **5** | 965.5 | 63 | 255.1 | 57 | 725.3 | 989.5 | 65 | 885.5 |
| **6** | 979.5 | 63 | 255.1 | 57 | 725.3 | 1003.5 | 65 | 899.4 |
| **7** and **8** | 787.5 | 63 | 239.1 | 57 | 547.2 | 811.6 | 65 | 707.5 |
| **9** | 801.5 | 63 | 239.1 | 57 | 547.2 | 825.5 | 65 | 721.4 |

[a]Precursor and product ion *m*/*z*, CE = collision energy (eV). For analyte identities, see Figure 1.

## Reference


1. Kilcoyne, J.; McCarron, P.; Twiner, M. J.; Rise, F.; Hess, P.; Wilkins, A. L.; Miles, C. O., Identification of 21,22-dehydroazaspiracids in mussels (*Mytilus edulis*) and in vitro toxicity of azaspiracid-26. *J. Nat. Prod.* **2018,** 81, (4), 885⊚893.
2. Hernández-Urcera, J.; Rial, P.; García-Portela, M.; Lourés, P.; Kilcoyne, J.; Rodríguez, F.; Fernández-Villamarín, A.; Reguera, B., Notes on the cultivation of two mixotrophic *Dinophysis* species and their ciliate prey *Mesodinium rubrum*. *Toxins* **2018,** 10, (12), 505.
3. Fux, E.; Marcaillou, C.; Mondeguer, F.; Bire, R.; Hess, P., Field and mesocosm trials on passive sampling for the study of adsorption and desorption behaviour of lipophilic toxins with a focus on OA and DTX1. *Harmful Algae* **2008,** 7, 574–583.
4. Fux, E.; Bire, R.; Hess, P., Comparative accumulation and composition of lipophilic marine biotoxins in passive samplers and in mussels (*M. edulis*) on the West Coast of Ireland. *Harmful Algae* **2009,** 8, (3), 523–537.
